# Between Elemental Match and Mismatch: From K_12_Ge_3.5_Sb_6_ to Salts of (Ge_2_Sb_2_)^2−^, (Ge_4_Sb_12_)^4−^, and (Ge_4_Sb_14_)^4−^

**DOI:** 10.1002/anie.202207232

**Published:** 2022-07-28

**Authors:** Katrin Beuthert, Fuxing Pan, Lukas Guggolz, Robert J. Wilson, Jan Hempelmann, Richard Dronskowski, Stefanie Dehnen

**Affiliations:** ^1^ Fachbereich Chemie und Wissenschaftliches Zentrum für Materialwissenschaften Philipps-Universität Marburg Hans-Meerwein-Straße 4 35043 Marburg Germany; ^2^ Chair of Solid-State and Quantum Chemistry Institute of Inorganic Chemistry RWTH Aachen University Landoltweg 1 52056 Aachen Germany; ^3^ Hoffmann Institute of Advanced Materials Shenzhen Polytechnic 7098 Liuxian Blvd Nanshan District, Shenzhen China

**Keywords:** Antimony, DFT Calculations, Germanium, Intermetallic Cluster Formation, Zintl Anions

## Abstract

The solid mixture “K_2_GeSb” was shown to comprise single‐crystalline K_12_Ge_3.5_Sb_6_ (**1**), a double salt of K_5_[GeSb_3_] with carbonate‐like [GeSb_3_]^5−^ anions, and the metallic Zintl phase K_2_Ge_1.5_. Extraction of **1** with ethane‐1,2‐diamine in the presence of crypt‐222 afforded [K(crypt‐222)]^+^ salts of several novel binary Zintl anions: (Ge_2_Sb_2_)^2−^ (in **2**), (Ge_4_Sb_12_)^4−^ (in **3**), and in the presence of [AuMePPh_3_] also (Ge_4_Sb_14_)^4−^ (in **4**). The anion in **2** represents a predicted, yet heretofore missing *pseudo‐*tetrahedral anion. **4** comprises a cluster analogous to (Ge_4_Bi_14_)^4−^ and (Ga_2_Bi_16_)^4−^, and thus one of the most Sb‐rich binary p‐block anions. The unprecedented cluster topology in **3** can be viewed as a defect‐version of the one in **4** upon following a “dead end” of cluster growth. The findings indicate that Ge and Sb atoms are at the border of a well‐matching and a mismatch elemental combination. We discuss the syntheses, the geometric structures, and the electronic structures of the new compounds.

## Graphical abstract

Extraction of the intermetallic phase “K_2_GeSb”, actually comprising the new single‐crystalline compound K_12_Ge_3.5_Sb_6_, yields the long sought‐for binary anion (Ge_2_Sb_2_)^2−^ and larger ones, (Ge_4_Sb_12_)^4−^ and (Ge_4_Sb_14_)^4−^, which place the Ge/Sb combination at the border of elemental match and mismatch. The yet unknown diversity of these binary species predicts a rich follow‐up chemistry.

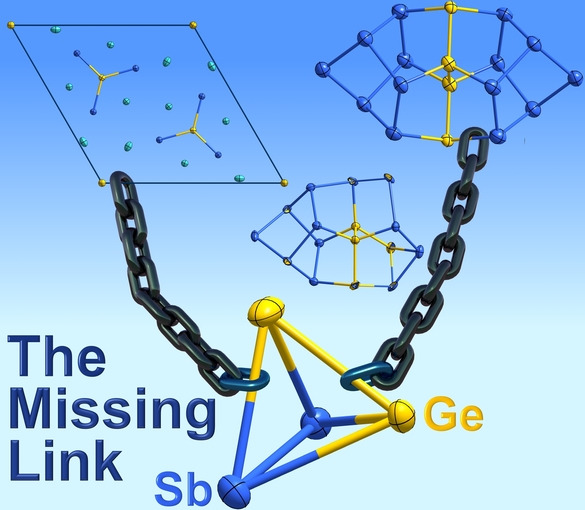

## Introduction

Investigating previously unexplored combinations of elements is always a journey into uncharted territories of the chemical landscape. In particular, new combinations of (semi)metals in cluster compounds provide insight into chemical bonds between atoms that have heretofore been unknown to interact, which in turn informs on a molecular scale about potential properties of novel intermetallic solids based on such elements. Zintl clusters are excellent objects for studying all kinds of intermetallic bonding in molecules.^[1]^

We are interested in exploring the interplay of atoms in clusters at the border of well‐matching and mismatching elemental combinations, as these can serve as interesting starting materials for large clusters of one of the involved elements on the one hand, or serve as the basis for uncommon and highly reactive binary structures on the other hand. As examples for elemental combinations that indicate a clear tendency for elemental segregation, we have reported about compounds that were obtained from ternary compounds based on the elements K, Ge, and Bi or K, Ga, and Bi, respectively. In the first case, both homoatomic and heteroatomic clusters were formed in reactions of a ternary solid of the nominal composition “K_5_Ga_2_Bi_4_” in en/crypt‐222 with d‐ or f‐block metal compounds (en=ethane‐1,2‐diamine; crypt‐222=4,7,13,16,21,24‐hexaoxa‐1,10‐diazabi‐cyclo[8.8.8]hexacosane) or reactions of the salt [K(crypt‐222)]_2_(GaBi_3_)⋅en^[2]^ obtained from the aforementioned solid by extraction in en/crypt‐222. Examples for clusters that emerged from such reactions are Bi_11_^3−^,^[3]^ (Ga_2_Bi_16_)^4−^,^[4]^ [{(cod)Ru}_4_Bi_18_]^4−^,^[4]^ [Sm@Ga_3−*x*_H_3−2*x*_Bi_10+*x*_]^3−^ (*x*=0, 1),^[5a]^ and [Th@Bi_12_]^4−^.^[5b]^ In the case of the elemental combination of K, Ge, and Bi, the only compound gained from a treatment of corresponding ternary solids, with the most efficient one being “K_2_GeBi”, has been [K(crypt‐222)]_4_(Ge_4_Bi_14_) so far.^[6]^ Notably, even the formation of a tetrahedral anion of the composition “(Ge_2_Bi_2_)^2−^” could not yet be secured—in contrast to the well‐known and extensively used anions of the homologous elemental combinations (Sn_2_Bi_2_)^2−^,^[7]^ (Sn_2_Sb_2_)^2−^,^[8]^ (Ge_2_As_2_)^2−^,^[9]^ or (Ge_2_P_2_)^2−^.^[10]^ The Ge/Bi combination was therefore described as a “mismatch combination” with poorly compatible atomic sizes, which is in line with the fact that Ge and Bi elements do not form solid mixtures at all, and that the two atom types in (Ge_4_Bi_14_)^4−^ anion are well separated.

Beyond the background that Ge/As represents a perfect match and Ge/Bi seems to be mostly incompatible, the elemental combination “in between”, Ge/Sb, remained to be explored. According to theoretical predictions, the tetrahedral anion with a 2 : 2 ratio of the two elements should exist (as for Ge/As but not for Ge/Bi, indeed),^[11]^ yet its isolation and characterization has been elusive to date. No further clusters comprising Ge besides Sb atoms were reported either to be obtained from a corresponding solid (while some molecular clusters based on (Ge_7_Sb_2_)^2–^ or (Ge_8_Sb)^3–^ anions were accessed on a different route).^[12a]^ Again, the miscibility of the two elements is poor, with a maximum solubility of 0.035 at% antimony in solid germanium (and a negligible solubility of Ge in solid Sb).^[12b]^ So, we decided to form a ternary solid with the nominal composition “K_2_GeSb” and study its chemical behavior.

**Scheme 1:**
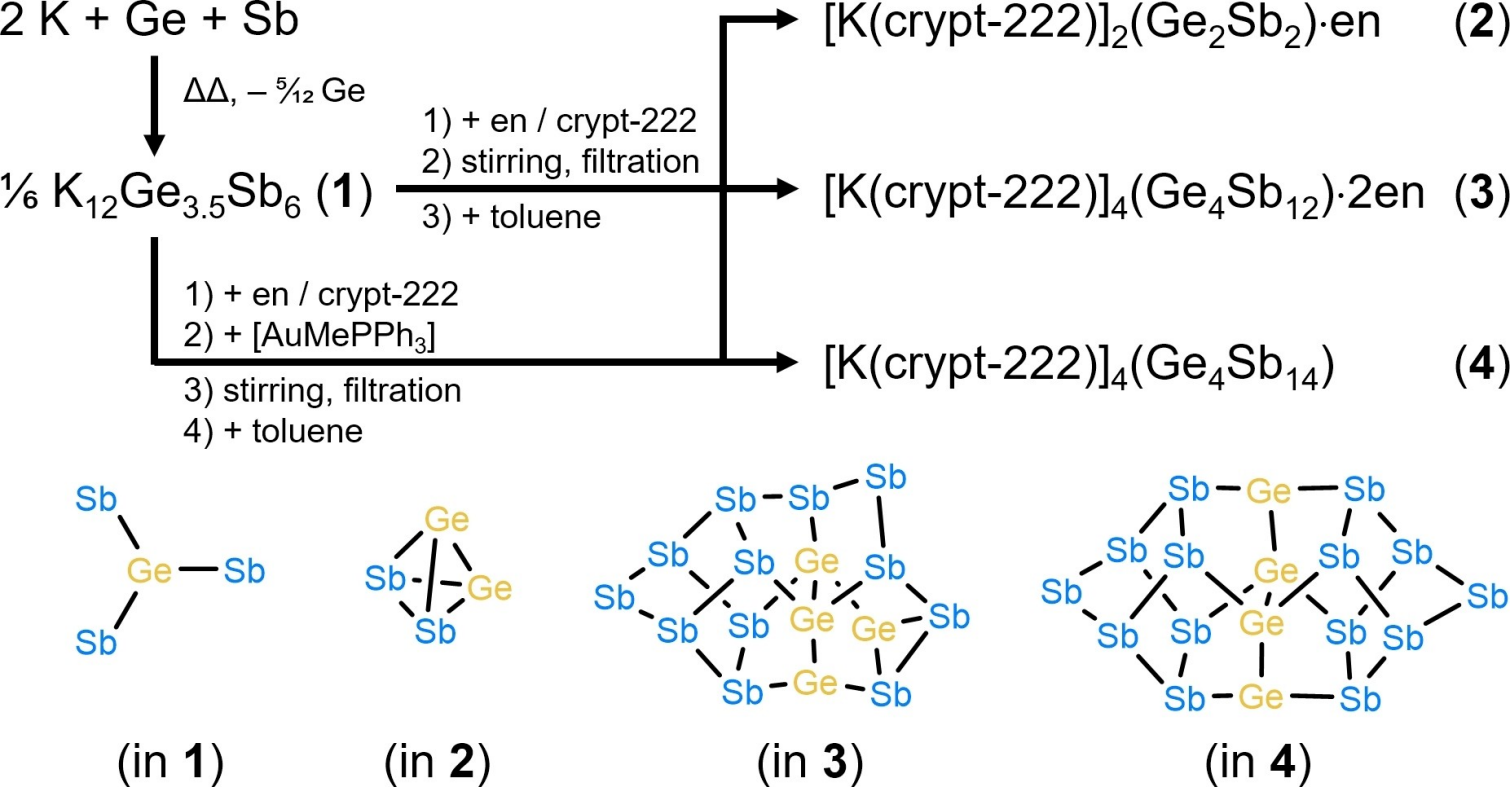
Formation of compounds **1**–**4** illustrated by non‐stoichiometric reaction schemes and structural diagrams of the molecular anions in **2**–**4** (atom assignment according to DFT calculations detailed below). Details on the synthesis procedures are given in the text and in the Experimental Section, the crystal structures and the computational results are shown and explained below.

Herein we describe our observations that indicate that the two elements represent a borderline case: they have a certain tendency for segregation, but also allow for the formation of anions with intense mixing of the different atoms. We obtained a new single‐crystalline ternary solid, K_12_Ge_3.5_Sb_6_ (**1**), which turned out to form upon fusion of 2 K+1 Ge+1 Sb. Its formation and that of the compounds obtained upon extracting compound **1** in en/crypt‐222 in the absence or presence of further metal compounds, [K(crypt‐222)]_2_(Ge_2_Sb_2_) (**2**), [K(crypt‐222)]_4_(Ge_4_Sb_12_) (**3**), and (in the presence of [AuMePPh_3_]) [K(crypt‐222)]_4_(Ge_4_Sb_14_) (**4**), are summarized in Scheme 1. The compounds were characterized by X‐ray diffraction,^[13]^ micro‐X‐ray fluorescence spectroscopy (μ‐XFS), electrospray ionization mass spectrometry (ESI‐MS), and density functional theory (DFT) studies (program system Turbomole,^[14]^ TPSS functional,^[15]^ dhf‐TZVP basis sets,^[16]^ COSMO,^[17]^ and program systems VASP^[18]^ and LOBSTER,^[19]^ using the PBEsol functional,^[20]^ for the solid state studies). Details of the syntheses, all analytical data, and the computational investigations are provided in the Supporting Information.

## Results and Discussion

**Figure 1:**
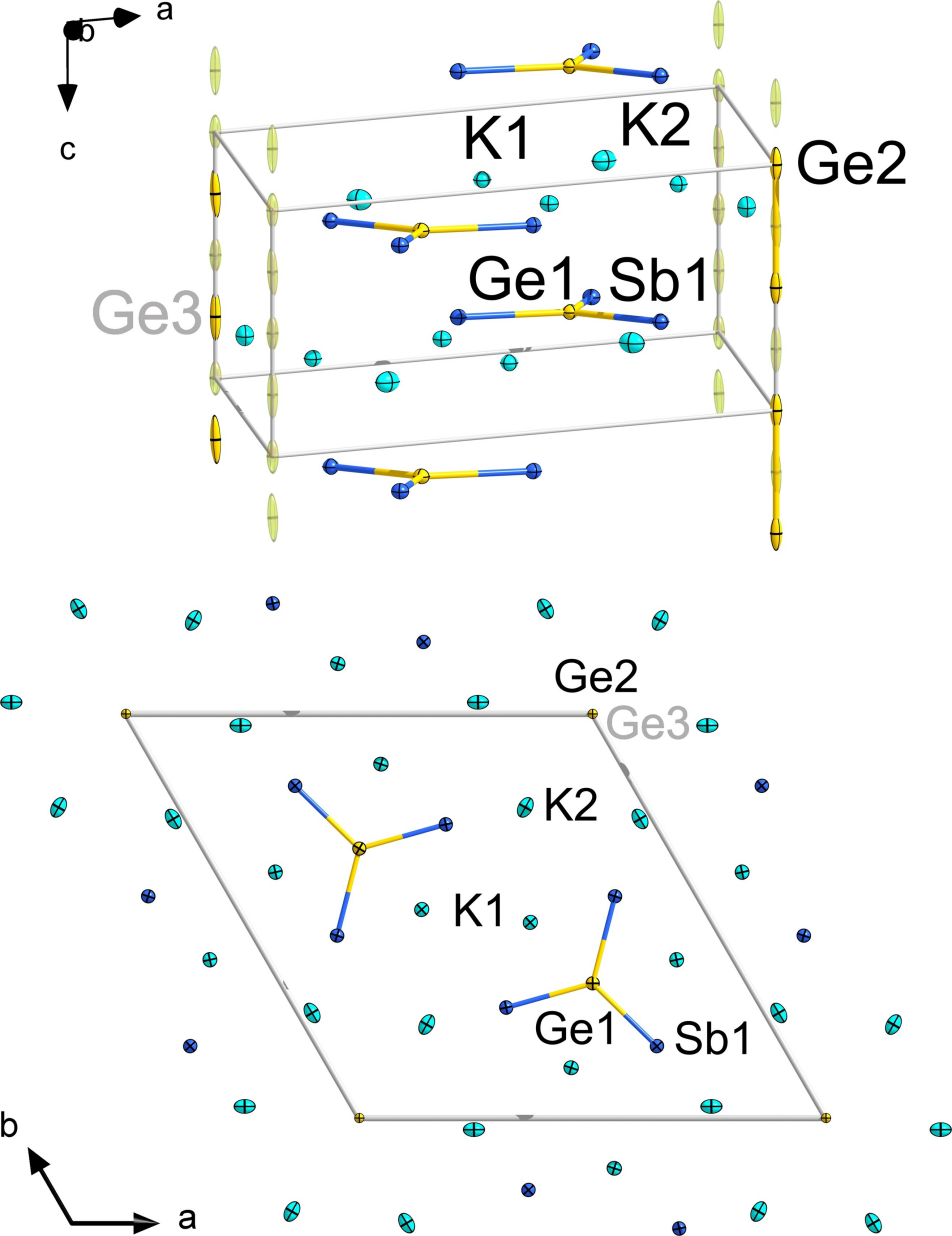
Two views of the crystal structure of compound **1** along two different crystallographic directions. Thermal ellipsoids are shown at the 30 % probability level. The partially occupied positions of Ge2 (1/2 occupation) and Ge3 (1/4 occupation) are shown in semi‐transparent mode, with one Ge−Ge dumbbell (Ge2) and one single Ge atom (Ge3) highlighted as solid ellipsoids. For clarity, only the K atoms that belong to the unit cell are drawn in the upper figure. Selected interatomic distances [Å] and angles [°]: Sb1−Ge1 2.5672(7), Ge2−Ge3 1.3398(3), Ge2(3)−Ge2(3) 2.6797(5), K⋅⋅⋅Sb 3.5464(17)–3.698(2), K⋅⋅⋅Ge 3.292(3)–3.6588(14); Ge1−Sb1−Ge1′ 120.0°. More structural details are provided in the Supporting Information.

According to powder X‐ray diffraction analyses (see Figure S1), the solid of the nominal composition “K_2_GeSb” is mostly equal to the single crystalline compound K_12_Ge_3.5_Sb_6_ (**1**), hence a solid in which ^5^/_12_ equivalents of Ge were not integrated relative to the stoichiometric ratio of the elements fused together. Indeed, small amounts of elemental Ge are detected beside the single‐crystalline reaction product. Single‐crystal X‐ray diffraction indicated that the latter represents a double salt of two new solids, two equivalents of K_5_GeSb_3_ and one equivalent of K_2_Ge_1.5_. The overall composition, K_12_Ge_3.5_Sb_6_, also represents the contents of the unit cell. The crystal structure of compound **1** is illustrated in Figure 1.

The K_5_GeSb_3_ part of **1** (K1, Ge1, Sb1 sites) comprises molecular, carbonate‐analogous, Ge‐centered [GeSb_3_]^5−^ anions which have been reported to exist in homologous elemental compositions only so far, [SiP_3_]^5−^, [SiAs_3_]^5−^, [GeP_3_]^5−^, [GeAs_3_]^5−^, [SnAs_3_]^5−^, and [SnBi_3_]^5−^.^[21]^ All of these anions represent perfectly planar triangular stars. Their relationship with the [CO_3_]^2−^ is not only suggested by *pseudo*‐element consideration, but can additionally be demonstrated by the corresponding canonical molecular orbitals (MOs, Figure S22).

In addition to this well‐comprehensible part, the solid comprises one equivalent of an unknown phase K_2_Ge_1.5_. This substructure, stoichiometrically refined by constrained isotropic displacement parameter for all Ge atoms, forms infinite strands along the crystallographic *c* axis, with equidistant Ge atoms in two subsets: Ge2 on 001/2 (refined occupation=0.50(1)≈1/2 ) and Ge3 on 001/4 (refined occupation=0.25(1)≈1/4 ), adding up to one Ge2 atom and half a Ge3 atom per unit cell (and thus, per formula unit). The Ge2−Ge2 distance is about 2.68(5) Å (that is, ≈0.2 Å longer than a typical Ge−Ge single bond, cf. 2.45 Å),^[22]^ and avoids an unphysical Ge2−Ge3 distance of 1.34(3) Å by suboccupancy. This composition is also refinable using anisotropic displacement parameters, yet leading to elongated displacement ellipsoids of atoms Ge2 and Ge3. Our interpretation, which is corroborated by the quantum chemical study described hereafter, is as follows: The formula unit comprises half an equivalent of a Ge_2_^2−^ dumbbell (Ge2 sites) and half a Ge^2−^ (Ge3 sites), hence 1.5 Ge atoms with two negative charges in total to compensate the charge of the two K^+^ ions (K2 sites). An alternative assignment of charges (1/2 ×“Ge_2_^4−^” and 1/2 ×Ge^0^)^[23]^ is in conflict with electroneutrality and solid‐state theory (see below). Furthermore, DFT calculations of Ge_2_^*q*−^ with *q*=2 and 4 indicate that the HOMO–LUMO energy gap of “Ge_2_^4−^” is not reasonable (0.9 eV), while that of Ge_2_^2−^ is fine (2.3 eV). Furthermore, the Ge−Ge distance of the dianion (2.24 Å; cf. 2.48 Å for the tetraanion) perfectly matches the value calculated for the supercell model (see below). Hence, the deviation from the (average) Ge−Ge distance observed for the (equidistant) Ge2/Ge3 sites in the crystal structure of **1** can be explained by the seemingly too simple crystallographic model for the statistically disordered Ge atoms along the *c* axis and, thus, the formation of a kind of “one‐dimensional solid solution”. This also explains the large thermal ellipsoids that indicate closer contacts of the atoms of the dumbbell and farther distances in all other cases.

Single‐stranded substructures were observed in other intermetallic solids, for instance in Pr_6_Ni_1.76_Si_3_,^[24a]^ Pr_5_Ni_1.9_Si_3_,^[24b]^ Ce_6_Ni_2_Si_3_,^[25]^ Nd_6_Co_1.67_Si_3_,^[26]^ Pr_6_Co_1.67_Si_3_,^[27]^ K_10_Ga_3_Sb_6.33_,^[28]^ K_10_Ga_3_As_6.33_,^[28]^ and K_10_Ga_3_Bi_6.65_,^[29]^ some of which were also interpreted as line‐up of disordered dumbbells.

**Figure 2:**
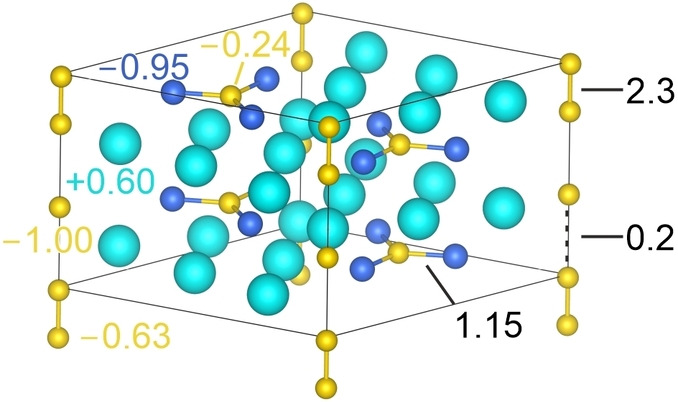
1×1×2 supercell for K_12_Ge_3.5_Sb_6_ with an alternating distribution of (Ge−Ge)^2−^ dumbbells (Ge2) and isolated Ge^2−^ ions (Ge3). While Löwdin charges are given on the left hand side in the corresponding atom colors (K, turquoise; Ge, yellow; Sb blue), bond orders expressed by the two‐center integrated COBI are shown on the right hand side.

To understand the situation within the strand of “Ge_2_^1.5−^” extending along the crystallographic *c* axis, solid state DFT calculations were undertaken under consideration of periodic boundary conditions. The computed structure model (Figure 2) is given as K_12_Ge_3.5_Sb_6_ in a supercell doubled along *c*, in which the Ge2 position is 50 % occupied and Ge3 is 25 % occupied, exactly like the free refinement of the atomic site occupations during X‐ray structure analysis; the supercell figure indicates wavefunction‐based Löwdin charges^[30]^ to the left and crystal orbital bond index (ICOBI; equivalent of the classical bond order)^[19d]^ for pairwise interactions, hence COBI^(2)^ and integral ICOBI^(2)^, to the right. The total bond energy amounts to −1159 kJ mol^−1^.

Bond analyses with Löwdin charges and COBI confirm that K_12_Ge_3.5_Sb_6_ exhibits Ge2−Ge2=2.27 Å dumbbells charged −0.63 atomwise with high bond order (2.3) besides an (unprecedented) isolated Ge^2−^ anion charged −1.00 with a low ICOBI of 0.2 reflecting the large distance of 3.98 Å to Ge2. Notably, the other parts of the structure are not affected by the situation in this substructure. We additionally observe some three‐center bonding within the Ge2−Ge2⋅⋅⋅Ge3 units, as demonstrated by the corresponding value of the three‐center ICOBI^(3)^ of −0.06, which is ≈20 % of the value calculated for XeF_2_ (−0.32).^[19d]^

**Figure 3:**
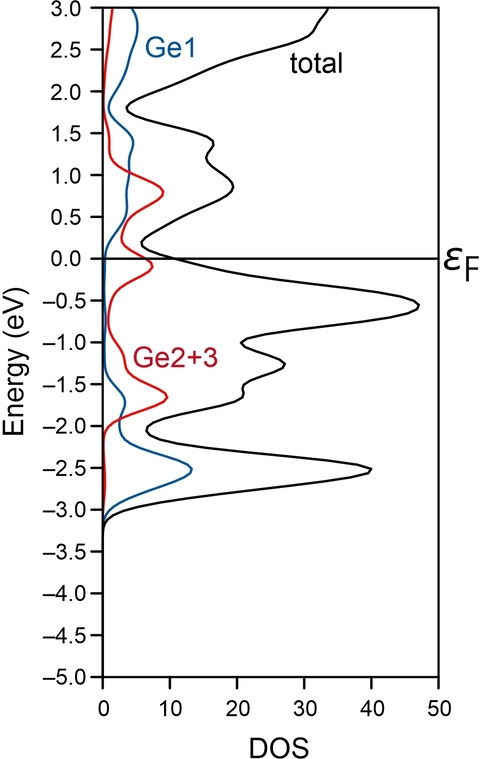
Total and local density of states of K_12_Ge_3.5_Sb_6_ near the Fermi energy level.

Figure 3 shows the total and local density of states of K_12_Ge_3.5_Sb_6_ close to the Fermi energy level. The DOS curve clearly demonstrates that this compound is an electric conductor (no semiconductor), which is mainly due to the contributions from Ge2 and Ge3—in agreement with the described bonding more within the strands along the *c* axis. Again, the other parts of the structure are almost entirely decoupled, also in terms of transport properties because they only depend on Ge2 and Ge3.

With the characterization of compound **1** we were thus able to unravel the heretofore unknown identity of solids with the nominal compositions “K_2_E^14^E^15^”. In related cases, in which the identity of this solid was not elucidated, extractions of “K_2_E^14^E^15^” with en/crypt‐222 led to the formation of [K(crypt‐222)]^+^ salts of the *pseudo*‐tetrahedral anions (E^14^_2_E^15^_2_)^2−^ in most cases, and to the salt of (Ge_4_Bi_14_)^4−^ for “K_2_GeBi”, all of which apparently have no structural relation with the anionic substructure of **1**. For this, we tested the extraction behavior of **1**, too. As discussed in the following, the carbonate‐like anions in **1**, together with the additional “Ge^2−^”, indeed undergo significant structural changes and also redox reactions to form the other species that finally crystallized as compounds **2**–**4**.

**Figure 4:**
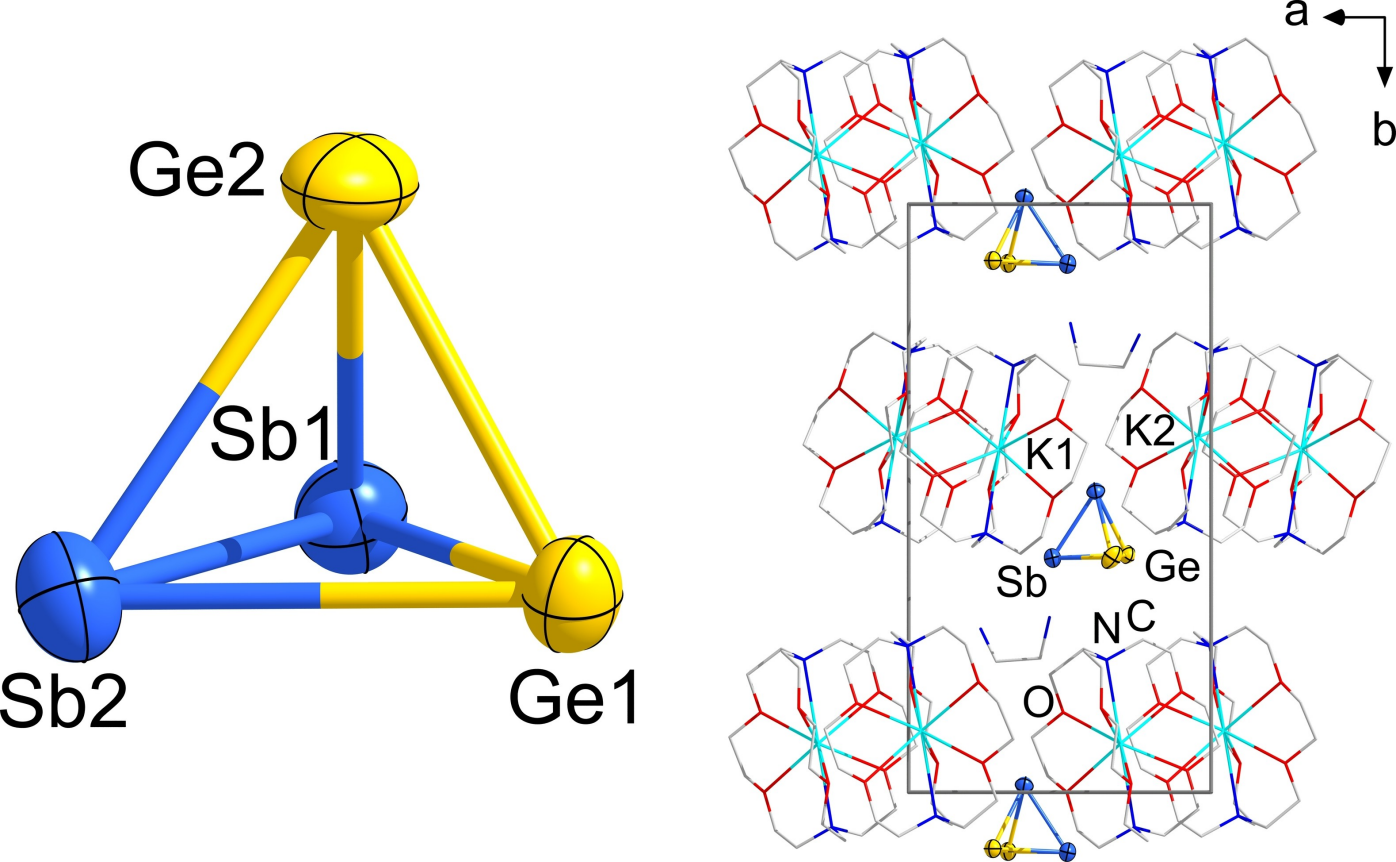
Molecular structure of the (Ge_2_Sb_2_)^2−^ anion in **2**. Thermal ellipsoids are shown at the 30 % probability level. Selected interatomic distances [Å] and angles [°]: Ge1−Ge2 2.5316(13), Sb1−Sb2 2.7889(8), Ge−Sb 2.6941(14) − 2.7057(10); Ge−Sb−Ge 55.84(3), 56.04(3), Ge−Sb−Sb 58.71(3)–59.10(3), Sb−Ge−Sb 62.19(3), 62.25(3), Sb−Ge−Ge 61.97(3)–62.17(3). More structural data is provided in the Supporting Information.

Treatment of 50 mg (0.183 mmol) of **1** in 5 mL of en/crypt‐222 for 24 hours at room temperature, filtration of the extraction solution and evaporation of the solvent until first crystals were visible afforded crystals of compounds **2** (orange sticks; ≈85 %), **3** (black blocks; ≈5 %), and **4** (dark brown ellipsoids; ≈10 %; in the presence of [AuMePPh_3_]) in approx. 70 % total yield based on Sb. While the three compounds co‐crystallize, it is clearly notable that the crystals of **2** form first. According to X‐ray diffraction, the anion in **2** represents the *pseudo*‐tetrahedral anion (Ge_2_Sb_2_)^2−^ (Figure 4) that was elusive so far although theoretically predicted.^[11]^

Different from other homologues (including (Sn_2_Bi_2_)^2−^), the assignment of Ge and Sb atoms is unambiguous and free of disorder for this anion, with well‐distinguishable Ge−Ge (2.5316(13) Å), Sb−Sb (2.7889(8) Å), and Ge−Sb (2.6941(14)–2.7057(10) Å) bonds. The reproducible formation of the tetrahedral anion in **2** with its formal Ge^−^ and Sb^0^ atoms (according to the *pseudo*‐element concept based on the number of adjacent bonds) from the carbonate‐like anion in **1** with (formally) Ge^0^, Sb^−^, and Sb^2−^ sites according to the *pseudo*‐element concept is not intuitive regarding structures and formal charges. We ascribe it to a combined intra‐ and intermolecular redox reaction as given in Equation 1.

(1) $$\left[\mathrm{Ge}{}^{0}\left(\mathrm{Sb}{}^{-}\right)\left(\mathrm{Sb}{}^{2-}\right){}_{2}\right]{}^{5-}\;\left(\mathrm{in}\;1\right)+\mathrm{Ge}{}^{2-}+2\;H{}_{2}N\text{-}\mathrm{CH}{}_{2}\mathrm{CH}{}_{2}\text{-}\mathrm{NH}{}_{2}\to \left[\left(\mathrm{Ge}{}^{-}\right){}_{2}\mathrm{Sb}{}^{0}{}_{2}\right]{}^{2-}\left(\mathrm{in}\;2\right)+\mathrm{Sb}{}^{3-}+2\;\left(H{}_{2}N\text{-}\mathrm{CH}{}_{2}\mathrm{CH}{}_{2}\text{-}\mathrm{NH}\right){}^{-}+H{}_{2}$$

If assuming that the carbonate‐like units in **1** serve as the basis for forming the *pseudo*‐tetrahedral anions in **2**, this process might comprise the following steps (under consideration of the formal charges of the atoms in **1**): (1) Intramolecular electron transfer from “Sb^−^” to “Ge^0^” to produce “Ge^−^” and “Sb^0^” sites. (2) Replacement of one “Sb^2−^” with “Ge^−^” and release of the first as “Sb^3−^” upon a single‐electron transfer from a free “Ge^2−^” from the phase. (3) The release of two remaining electrons from the second “Sb^2−^” site in the molecule to form “Sb^0^” and finally close the P_4_‐like *pseudo‐*tetrahedron; these electrons serve to reduce two protons from en molecules under formation of one equivalent of H_2_ as an important driving force. We cannot say whether these steps occur sequentially or together, but both would be generally conceivable given very short periods of time. The formation of Sb^3−^ and H_2_ were concluded from the fact that a ternary “K_2_SnSb” solid was proven to be a source for Sb^3−^ under similar conditions in a recent study, in which the reductive deprotonation and H_2_ formation from en has been proven to occur during extraction of Zintl phases in this solvent.^[31]^ Moreover, no other reduced species were observed nor proven to form in this reaction, and any other reaction scheme set up with known species fails to yield a proper charge balance. Hence, we conclude that the suggested reaction scheme provides a plausible explanation for the electron transfer processes.

**Figure 5:**
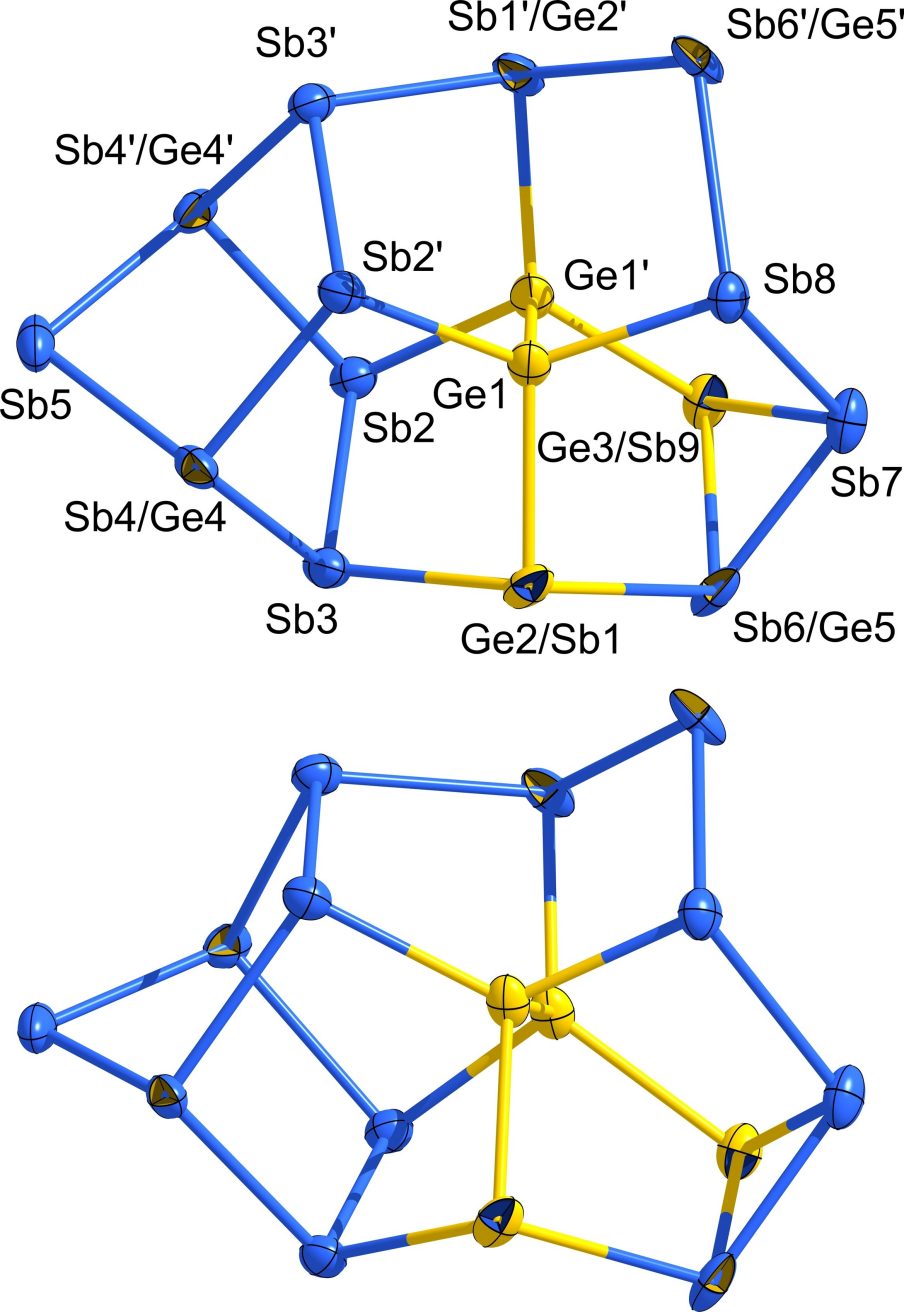
Molecular structure of the (Ge_4_Sb_12_)^4−^ anion in **3** in two slightly different views. Thermal ellipsoids are shown at the 30 % probability level in two different orientations. For clarity, the positional disorder of Sb7, Sb8, and Ge3/Sb9 is not shown here (for more details, see the Supporting Information). Atomic sites with mixed Ge/Sb occupancy are indicted by two‐colored octants (for details regarding the occupation factors, see the CIF). The isomer that accords with the energetic minimum structure according to DFT calculations (see below) is highlighted by choosing the corresponding dominant colors of the octants. Selected distances [Å] (given for the majority atom type if not specified otherwise): Ge1−Ge1′ 2.4538(17), Ge1−Sb 2.5214(14)–2.5827(11), Sb(2,3,4)−Sb 2.7873(9)–2.8103(8), Sb5−Sb 2.7613(8), Sb6−Sb(7,9) 2.5758(17), 2.6790(13), Sb7−Sb(8,9) 2.7613(18)–2.8732(18), Sb8−Sb6′ 2.8929(14). More structural data is provided in the Supporting Information.

Having shown that intense mixing of Ge and Sb atoms (2 : 2) can be realized on the molecular scale in contrast to the very poor mutual solubility of the elements in each other, the observation of compound **3** indicates the variability of this elemental combination. Here we find a tendency towards element segregation, with well‐defined {Ge_*x*_} and {Sb_*y*_} subunits, but also some mixing of both atom types in the molecular architecture, which is illustrated in Figure 5. Therefore, the {Ge_*x*_Sb_*y*_} elements combination can be regarded as the most tolerant one described so far.

The anion in compound **3** exhibits a yet unknown molecular structure, which is related, yet not identical to those reported for (Ge_4_Bi_14_)^4−^ or (Ga_2_Bi_16_)^4−^.[^4^, ^6^] The molecular architecture and the bonding situation of the anion in **3** will be discussed in comparison with the corresponding properties of the structurally related anion in **4** below.

**Figure 6:**
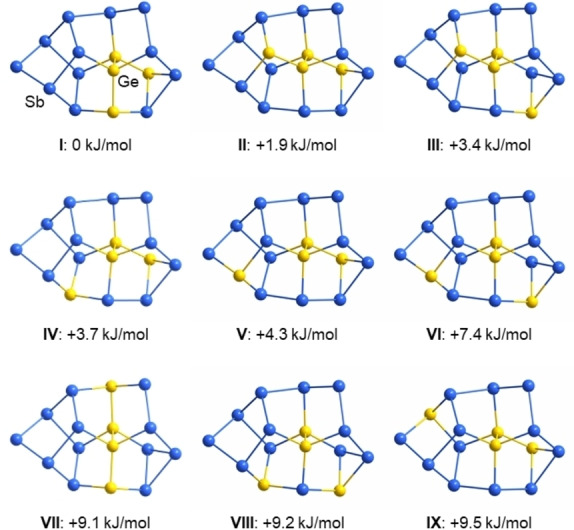
Illustration of the nine nearly isoenergetic isomers of the (Ge_4_Sb_12_)^4−^ anion according to DFT calculations. Distances [Å]: Ge−Ge 2.47–2.50, Ge−Sb 2.62–2.75, Sb−Sb 2.79–2.90. Relative energies Δ*E* are given with respect to the total energy calculated for the global minimum structure (isomer **I**).

The determination of the molecular structure of the anion in **3** by means of X‐ray diffraction was not trivial, as in the unsymmetrical cluster half, atoms Sb7–Sb9 are disordered over two positions that are symmetry‐generated by the *C*_2_ axis running through Sb5 and the center of the Ge1−Ge1′ bond. As a consequence, the two symmetry‐equivalent sites of Sb6 act as either two‐bonded or three‐bonded atoms (50 : 50 each), which is visible in larger thermal ellipsoids for the slightly different coordination environment in the two cases. As the rotational disorder is statistical, a symmetry reduction (down to space group *P*1) did not serve to resolve the two positions. In addition, four of the atomic sites in the asymmetric unit show a mixed occupation (10 % of Ge on the Sb1 site, 20 % of Ge on the Sb4 and Sb9 sites, 40 % of Sb on the Ge5 site) according to the structure refinement. In Figure 5, this is accounted for by two‐colored atoms, the dominant color of which represents the atom type that occupies this site in the global minimum structure according to the DFT studies (see Figure 6). As discussed below, eight other isomers are very close in energy, hence the site occupation factors most likely represent a superposition of all of them.

Owing to the rotational disorder and mixed‐site occupancies, the assignment of two of the Ge atoms required the use of comprehensive DFT calculations. For determining the stabilities of different isomers of (Ge_4_Sb_12_)^4−^, the two Ge atoms on positions Ge1 and Ge1′ as well as the Sb atoms on positions Sb5 and Sb6′/Ge5′ were fixed, as these sites are clearly occupied with said atom types given their fourfold (Ge) and twofold (Sb) connections (isomers where either one or both of the “inner” Ge positions are occupied with an Sb atom are by at least 105 kJ mol^−1^ disfavored in energy). The remaining two Ge atoms were thus placed on all positions except Sb5 or Sb6′/Ge5′, leading to 20 isomers in total. The isomers’ structures and selected interatomic distances are depicted in Figure S25. Notably, nine of the isomers (**I**–**IX**, Figure 6) are nearly isoenergetic (Δ*E*=2–10 kJ mol^−1^).

**Figure 7:**
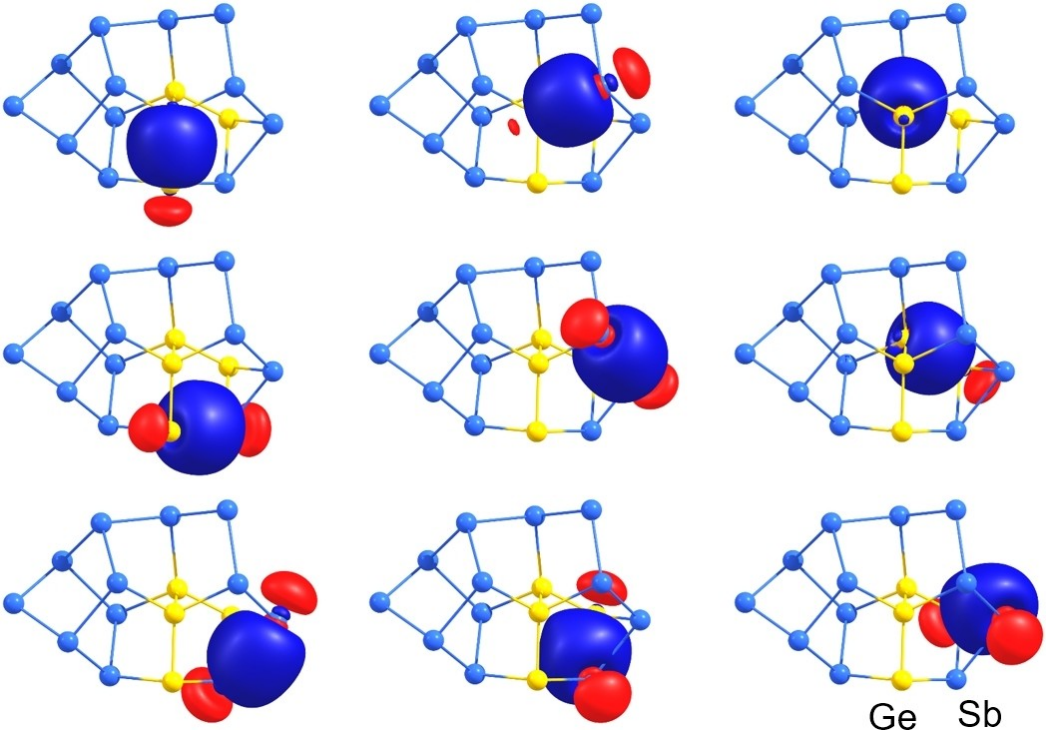
LMOs representing the nine two‐center bonds within the nortricyclane‐type {Ge_4_Sb_3_} subunit of the energetically most favorable isomer of the anion of **3**: Ge−Ge and Ge−Sb bonds including the apical Ge atoms (top row), Ge−Ge, Ge−Sb, and Sb−Sb bonds between the apical Ge atom and the basal {GeSb_2_} triangle (middle row), Ge−Sb and Sb−Sb bonds in the basal triangle (bottom row). Contour values are drawn at ±0.05 a.u.

These isomers need to be considered as co‐existent—consistent with the observed disorder of the two Ge atoms over most of the remaining atomic sites and the elongated thermal ellipsoids for mixed atomic sites, in which the assignment of split positions failed (note that mixed occupancies of less than 1 : 9 were not modelled in the X‐ray structure refinement, but are suggested by the calculations to be generally possible). Seven further isomers are by 14 (**X**), 16 (**XI**), 22 (**XII**–**XIV**) or 23 (**XV**, **XVI**) kJ mol^−1^ higher in energy than the global minimum isomer **I**, and thus less likely to contribute significantly to the crystal structure. Four remaining isomers are by 39 (**XVII**), 40 (**XVIII**), 50 (**XIX**), or 53 kJ mol^−1^ (**XX**) less favorable than the minimum isomer and will therefore not be probable alternatives to the other variants. Inspection of localized molecular orbitals (LMOs, Figure 7 and Figure S26)^[32]^ show exclusively regular two‐electron two‐center (2e2c) bonds. This is also the case for the triangular face Ge3/Sb9−Sb6/Ge5−Sb7 and rationalizes the electron‐precise nature of this anion.

**Figure 8:**
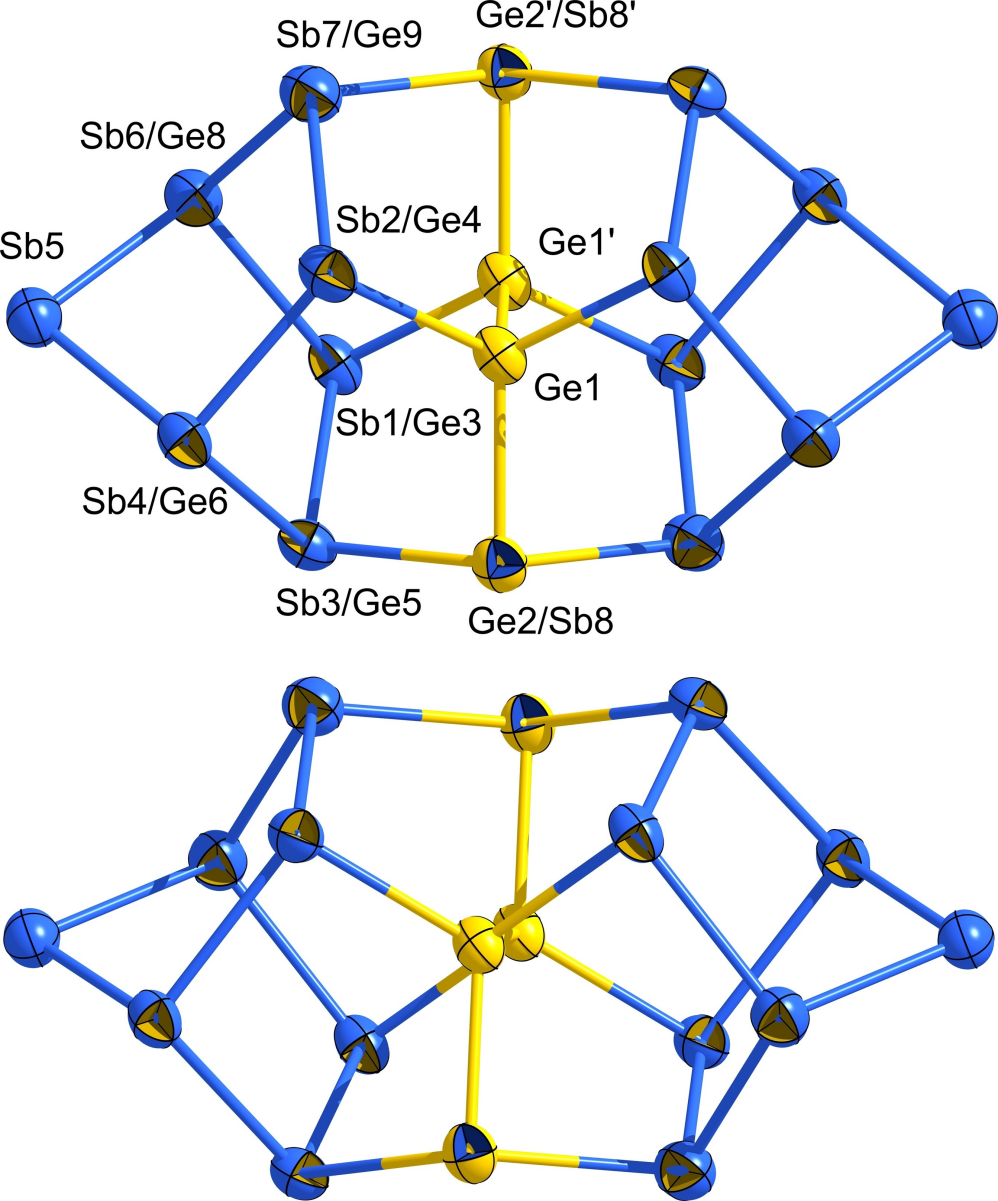
Molecular structure of the (Ge_4_Sb_14_)^4−^ anion in **4** in two slightly different views. Thermal ellipsoids are shown at the 30 % probability level. Atomic sites with mixed Ge/Sb occupancy are indicted by two‐colored octants (for details regarding the occupation factors, see the CIF). The isomer that accords with the energetic minimum structure according to DFT calculations (see below) is highlighted by choosing the corresponding dominant colors of the octants. Selected distances [Å] (given for the majority atom type): Ge1−Ge1′ 2.4302(25), Ge1−Ge2 2.5322(24), Ge(1,2)−Sb 2.5633(23)–2.7769(20), Sb(1,2)−Sb 2.7611(21)–2.7998(18), Sb(3,7)−Sb 2.7611(21)–2.8125(19), Sb5−Sb 2.7588(20)–2.7809(22). More structural data is provided in the Supporting Information.

The diverse nature of the K/Ge/Sb system was finally proven with the elemental segregation observed at (a) the co‐crystallization of compounds **2** and **3** with crystals of (fully element‐segregated) [K(crypt‐222)]_3_Sb_7_,^[33]^ and (b) the formation of compound **4**. Addition of [AuMePPh_3_] during extraction under the same conditions as used for the synthesis of **2** and **3** afforded a few, dark red crystals of compound **4** in one of our reactions, comprising an anion that is highly related to the one observed in compound **3** (Figure 8). This anion represents the lighter homologue of (Ge_4_Bi_14_)^4−^, which has been the only binary Zintl anion comprising Ge and Bi atoms at all to date, and which was obtained from a ternary solid with the nominal composition “K_2_GeBi”. It is also isoelectronic and isostructural to (Ga_2_Bi_16_)^4−^, one of the anions gained from the ternary solid “K_5_Ga_2_Bi_4_” in the presence of Lewis‐acidic metal complexes. Besides the high versatility of the K/Ge/Sb elemental combination, the isolation of **4** once again underscores the usefulness of Lewis acids in the assembly of large p‐block anions.

Like its homologue, the cluster can be described as consisting of two norbornadiene‐like {Sb_7_} units that are connected via a central substructure representing a {Ge_4_} chain. Another description is based on the “ufosane”‐type 11‐atom topology formed by one of the {Sb_7_} units and the {Ge_4_} unit, to which the second {Sb_7_} moiety is connected.

**Figure 9:**
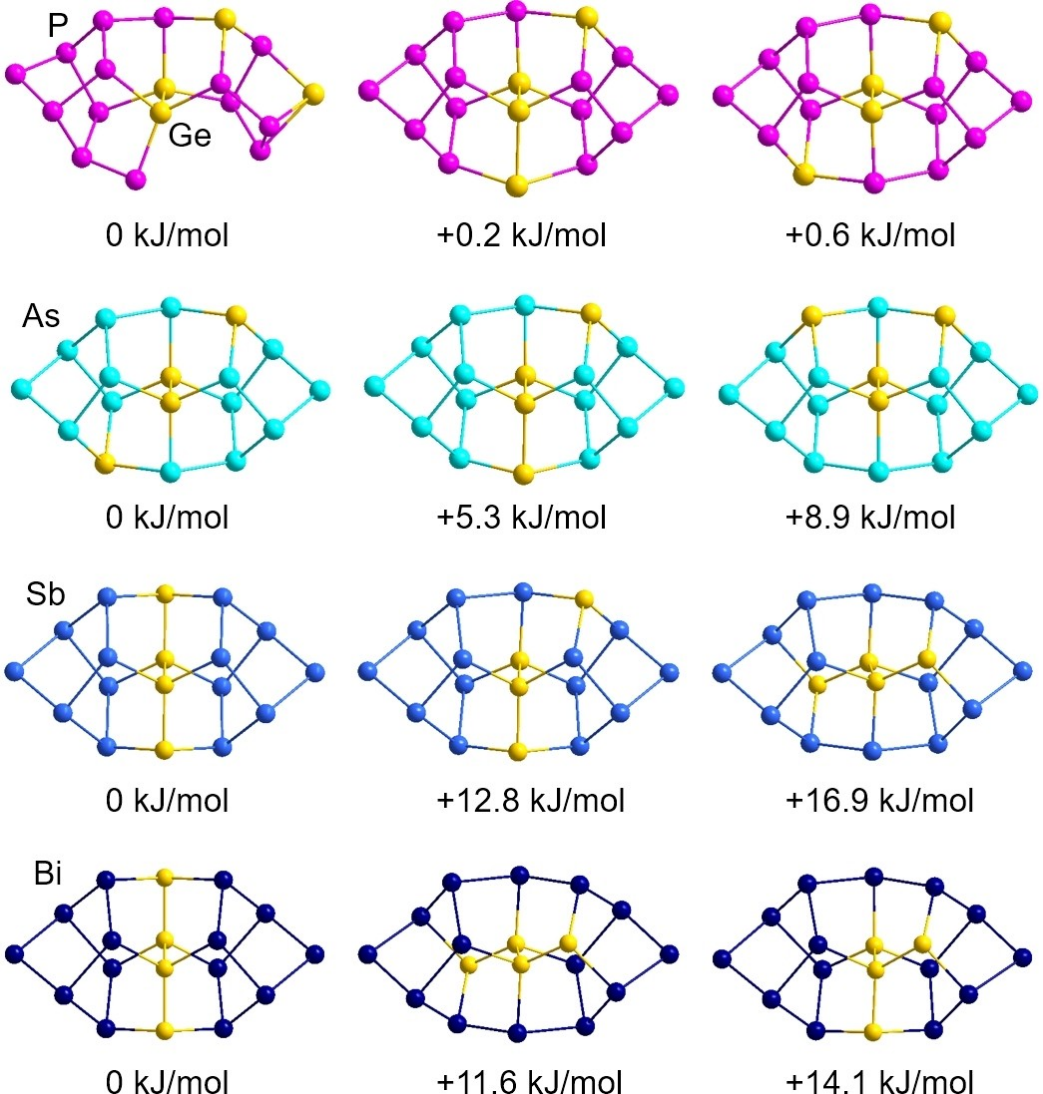
Comparison of the three isomers of lowest energy for the homologous anions (Ge_4_Pn_14_)^4−^ for Pn=P (top), As (second from top), Sb (second from bottom), and Bi (bottom) according to DFT calculations. Relative energies Δ*E* are given with respect to the total energy calculated for the global minimum structure in each series. A summary of all isomers of these species are provided in the Supporting Information (Figures S30–S33).

As indicated by two‐colored atoms in Figure 8, 14 of the 18 atoms within the anion again show partial replacement with the other element type—40 % of Sb on the Ge2 and Ge2′ sites, 10 % of Ge on six of the Sb sites (Sb1−Sb4, Sb6, Sb7 and symmetry equivalents). Such mixed occupations were also observed for (Ge_4_Bi_14_)^4−^ and are even more distinct here owing to the closer similarity of atomic radii. In accordance with this finding, the calculations showed a slightly different order of the respective isomers, although the relative energies Δ*E* were roughly in the same range. To get a deeper insight into the differences that come along with different elemental combinations, we computed all isomers of the anions (Ge_4_Pn_14_)^4−^ for Pn=P, As, Sb, Bi. As expected, the changes in radii match/mismatch is reflected in (a) the energetic order of the isomers and (b) their relative energies Δ*E*. This holds prominently for Ge/P and Ge/As. Due to the better miscibility and thus more degrees of freedom, the range of the relative energies Δ*E* is smaller for the energetically favored isomers and larger for the less favored ones. Figure 9 summarizes the three isomers of lowest energies for said elemental combinations along with the respective values of Δ*E*. The maximum energy difference between that of the global minimum structure and that of the isomer of highest energy amounts to 106 kJ mol^−1^ for Ge/P, 168 kJ mol^−1^ for Ge/As, 152 kJ mol^−1^ for Ge/Sn, and 135 kJ mol^−1^ for Ge/Bi—hence well reflecting the match versus mismatch of atomic radii. This trend can be attributed to the respective electron densities at the two‐bonded sites.

The anions in **3** and **4** both possess a total charge of 4−, which is an ideal charge for an anion of this size for crystallization along with (four) [K(crypt‐222)]^+^ counterions. Although one might formally attribute the total charge to the presence of 4 Ge atoms (“Ge^−^” in the reactant), this is actually not correct: Both structures include two‐connected Sb atoms, which formally carry a charge, while the two inner Ge atoms are four‐connected in both anions, and thus formally neutral. In both clusters, however, two two‐connected “Sb^−^” and two three‐connected “Ge^−^” can be attributed the four charges according to the *pseudo*‐element concept. This is in good agreement with the presence of two (“Sb^−^”) or one (“Ge^−^”) lone pair(s) at these atomic sites according to an analysis of the molecular orbitals (MOs) by Boys’ localization procedures. As expected, we again only found 2e2c bonds for the anion in **4** (see Figure S28).

At first glance, one may get the impression that the (Ge_4_Sb_12_)^4−^ cluster in **3** is an incomplete version of the (Ge_4_Sb_14_)^4−^ anion in **4**. Indeed, both species exhibit four Ge atoms, two of which are located in the center of the anion. However, the two remaining Ge atoms occupy different positions in **3** and **4**, which leads to different connection modes of the atoms that are located at the outer parts of the anion. Note that the position of the third Ge atom at one end of the central chain of the (Ge_4_Sb_12_)^4−^ anion in **3** (see Figure 5) was drawn to illustrate the computed minimum structure, but other positions can be occupied with similar possibilities according to both the isomeric structures and the structure refinements. Thus, the arrangement of the four Ge atoms relative to the Sb atoms in the two cluster halves defines (a) the overall shape of the cluster anion and (b) also its final composition that seems to stop as soon as four negative charges are reached. Therefore, the initial states of the cluster formation reaction determine whether the anion grows to the 18‐atom cage or whether its growth stops at the 16‐atom alternative.

To date, we cannot tell how the anions form, as they do not provide us with a spectroscopic handle for NMR, and as the larger anions tend to fragment under ESI‐MS conditions, which prohibits a time‐dependent analysis of their formation by means of mass spectrometry. So we can only speculate about a synthetic relationship between them. However, all of the anions form from compound **1** in a combination of consecutive redox steps that are yet to be elucidated. This is a very difficult task as electron‐transfer reactions between anions cannot be reasonably modeled by means of quantum chemistry.

However, we note that common structural motifs occur in all of the anions, especially 7‐atom cages of nortricyclane (**3**) or norbornadiene (**3** and **4**) topologies, indicating similar formation pathways of such anions in general. While the norbornadiene cages are (predominantly) homoatomic, the nortricyclane‐type cage in **3** is a rare heteroatomic one with the nominal composition “(Ge_4_Sb_3_)^7−^” in the most energetically favorable isomer. Such kind of a species has not been isolated so far for the group 14/15 elemental combination, but a related one, (Sn_3_Sb_4_)^6−^ was reported to form upon extraction of K_8_SnSb_4_ ^[34]^ in liquid ammonia. Upon addition of ZnPh_2_ to such extractions, a complex of a corresponding 2 : 5 anion was obtained, [(ZnPh)_2_(Sn_2_Sb_5_)]^3−^.^[8]^ A partially oxidized version of this binary anion, “(Sn_2_Sb_5_)^3−^”, was captured recently in transition metal complexes like [{Mo(CO)_3_}_2_(Sn_2_Sb_5_)]^3−^.^[35]^

## Conclusion

This study showed that a combination of K, Ge, and Sb atoms in Zintl compounds leads to an unexpected variability of accessible molecular anions. Owing to their particular relative values of atomic sizes and redox potentials, which goes hand in hand with the strength of the Ge−Sb bond versus the Ge−Ge and Sb−Sb bond strengths, Ge and Sb atoms were proven to be at the boundary of match and mismatch combinations, thus situated between those that prefer to mix with preferably heteroatomic bonds in follow‐up reactions and those that prefer to segregate. Hence, the new Zintl phase K_12_Ge_3.5_Sb_6_ (**1**) and the salts of anions gained from its extractions in en/crypt‐222, (Ge_2_Sb_2_)^2−^ (in **2**), (Ge_4_Sb_12_)^4−^ (in **3**) and (Ge_4_Sb_14_)^4−^ (in **4**), form a perfect starting‐point for a rich follow‐up chemistry. The tendency for an interplay of Ge and Sb atoms on the one hand, and a beginning element segregation tendency on the other hand, will enable access of both ternary (semi)metal clusters and antimony‐rich clusters when treating the new compounds with d‐ or f‐block metal compounds. This flexibility has been unprecedented in Zintl ion chemistry and will be the studied intensely from now on.

## Conflict of interest

The authors declare no conflict of interest.

1

## Supporting information

As a service to our authors and readers, this journal provides supporting information supplied by the authors. Such materials are peer reviewed and may be re‐organized for online delivery, but are not copy‐edited or typeset. Technical support issues arising from supporting information (other than missing files) should be addressed to the authors.

Supporting Information

Supporting Information

Supporting Information

Supporting Information

Supporting Information

Supporting Information

Supporting Information

Supporting Information

Supporting Information

Supporting Information

Supporting Information

## Data Availability

The data that support the findings of this study are available in the Supporting Information of this article.
