# Supplementary material for: Between Elemental Match and Mismatch: From K12Ge3.5Sb6 to Salts of (Ge2Sb2)2−, (Ge4Sb12)4−, and (Ge4Sb14)4−
Source: Angew Chem Int Ed Engl. 2022 Jul 28;61(41):e202207232. doi: 10.1002/anie.202207232 (PMC9796001; doi:10.1002/anie.202207232)

checkCIF/PLATON report

Structure factors have been supplied for datablock(s) 2

THIS REPORT IS FOR GUIDANCE ONLY. IF USED AS PART OF A REVIEW PROCEDURE FOR PUBLICATION, IT SHOULD NOT REPLACE THE EXPERTISE OF AN EXPERIENCED CRYSTALLOGRAPHIC REFEREE.

No syntax errors found. CIF dictionary Interpreting this report

Datablock: 2

Bond precision:	C-C = 0.0128 A		Wavelength=0.71073
Cell:	a=11.9235(5)	b=20.4010(6)	c=12.4643(5)
	alpha=90	beta=118.271(3)	gamma=90
Temperature:	100 K		

	Calculated	Reported
Volume	2670.30(19)	2670.30(19)
Space group	P 21	P 1 21 1
Hall group	P 2yb	P 2yb
Moiety formula	2(C18 H36 K N2 O6), Ge2 Sb2, C2 H8 N2	Ge2 Sb2, 2(C18 H36 K N2 O6), C2 H8 N2
Sum formula	C38 H80 Ge2 K2 N6 O12 Sb2	C38 H80 Ge2 K2 N6 O12 Sb2
Mr	1280.02	1279.96
Dx,g cm-3	1.592	1.592
Z	2	2
Mu (mm-1)	2.329	2.329
F000	1300.0	1300.0
F000’	1299.49	
h,k,lmax	16,28,17	16,28,17
Nref	14485[7437]	14393
Tmin,Tmax	0.756,0.890	0.756,0.890
Tmin’	0.628	

Correction method= # Reported T Limits: Tmin=0.756 Tmax=0.890
AbsCorr = NUMERICAL

Data completeness= 1.94/0.99 Theta(max)= 29.220

R(reflections)= 0.0440(9317) wR2(reflections)= 0.1030(14393)

S = 0.885 Npar= 559

The following ALERTS were generated. Each ALERT has the format
test-name_ALERT_alert-type_alert-level.
Click on the hyperlinks for more details of the test.

Alert level C

```
ABSTY02_ALERT_1_C  An _exptl_absorpt_correction_type has been given without
            a literature citation. This should be contained in the
            _exptl_absorpt_process_details field.
            Absorption correction given as numerical
PLAT243_ALERT_4_C High   'Solvent' Ueq as Compared to Neighbors of        C38 Check
PLAT244_ALERT_4_C Low    'Solvent' Ueq as Compared to Neighbors of        C37 Check
PLAT260_ALERT_2_C Large Average Ueq of Residue Including        N5      0.104 Check
PLAT342_ALERT_3_C Low Bond Precision on  C-C Bonds ...............    0.01279 Ang.
PLAT410_ALERT_2_C Short Intra H...H Contact  H2A       ..H12A     .       1.99 Ang.
                                                       x,y,z  =      1_555 Check
PLAT420_ALERT_2_C D-H Bond Without Acceptor  N5       --H5C      .     Please Check
PLAT420_ALERT_2_C D-H Bond Without Acceptor  N5       --H5D      .     Please Check
PLAT420_ALERT_2_C D-H Bond Without Acceptor  N6       --H6C      .     Please Check
PLAT420_ALERT_2_C D-H Bond Without Acceptor  N6       --H6D      .     Please Check
PLAT976_ALERT_2_C Check Calcd Resid. Dens.  0.47Ang From N6      .      -0.70 eA-3
```

Alert level G

```
PLAT007_ALERT_5_G Number of Unrefined Donor-H Atoms ..............          4 Report
PLAT042_ALERT_1_G Calc. and Reported Moiety Formula Strings Differ     Please Check
PLAT066_ALERT_1_G Predicted and Reported Tmin&Tmax Range Identical          ? Check
PLAT912_ALERT_4_G Missing # of FCF Reflections Above STh/L=  0.600         32 Note
PLAT978_ALERT_2_G Number C-C Bonds with Positive Residual Density.          0 Info
```

0 **ALERT level A** = Most likely a serious problem - resolve or explain
0 **ALERT level B** = A potentially serious problem, consider carefully
11 **ALERT level C** = Check. Ensure it is not caused by an omission or oversight
5 **ALERT level G** = General information/check it is not something unexpected

3 ALERT type 1 CIF construction/syntax error, inconsistent or missing data
8 ALERT type 2 Indicator that the structure model may be wrong or deficient
1 ALERT type 3 Indicator that the structure quality may be low
3 ALERT type 4 Improvement, methodology, query or suggestion
1 ALERT type 5 Informative message, check

It is advisable to attempt to resolve as many as possible of the alerts in all categories. Often the minor alerts point to easily fixed oversights, errors and omissions in your CIF or refinement strategy, so attention to these fine details can be worthwhile. In order to resolve some of the more serious problems it may be necessary to carry out additional measurements or structure refinements. However, the purpose of your study may justify the reported deviations and the more serious of these should normally be commented upon in the discussion or experimental section of a paper or in the "special_details" fields of the CIF. checkCIF was carefully designed to identify outliers and unusual parameters, but every test has its limitations and alerts that are not important in a particular case may appear. Conversely, the absence of alerts does not guarantee there are no aspects of the results needing attention. It is up to the individual to critically assess their own results and, if necessary, seek expert advice.

Publication of your CIF in IUCr journals

A basic structural check has been run on your CIF. These basic checks will be run on all CIFs submitted for publication in IUCr journals (*Acta Crystallographica*, *Journal of Applied Crystallography*, *Journal of Synchrotron Radiation*); however, if you intend to submit to *Acta Crystallographica Section C* or *E* or *IUCrData*, you should make sure that full publication checks are run on the final version of your CIF prior to submission.

Publication of your CIF in other journals

Please refer to the *Notes for Authors* of the relevant journal for any special instructions relating to CIF submission.

PLATON version of 19/02/2022; check.def file version of 19/02/2022

Datablock 2 - ellipsoid plot

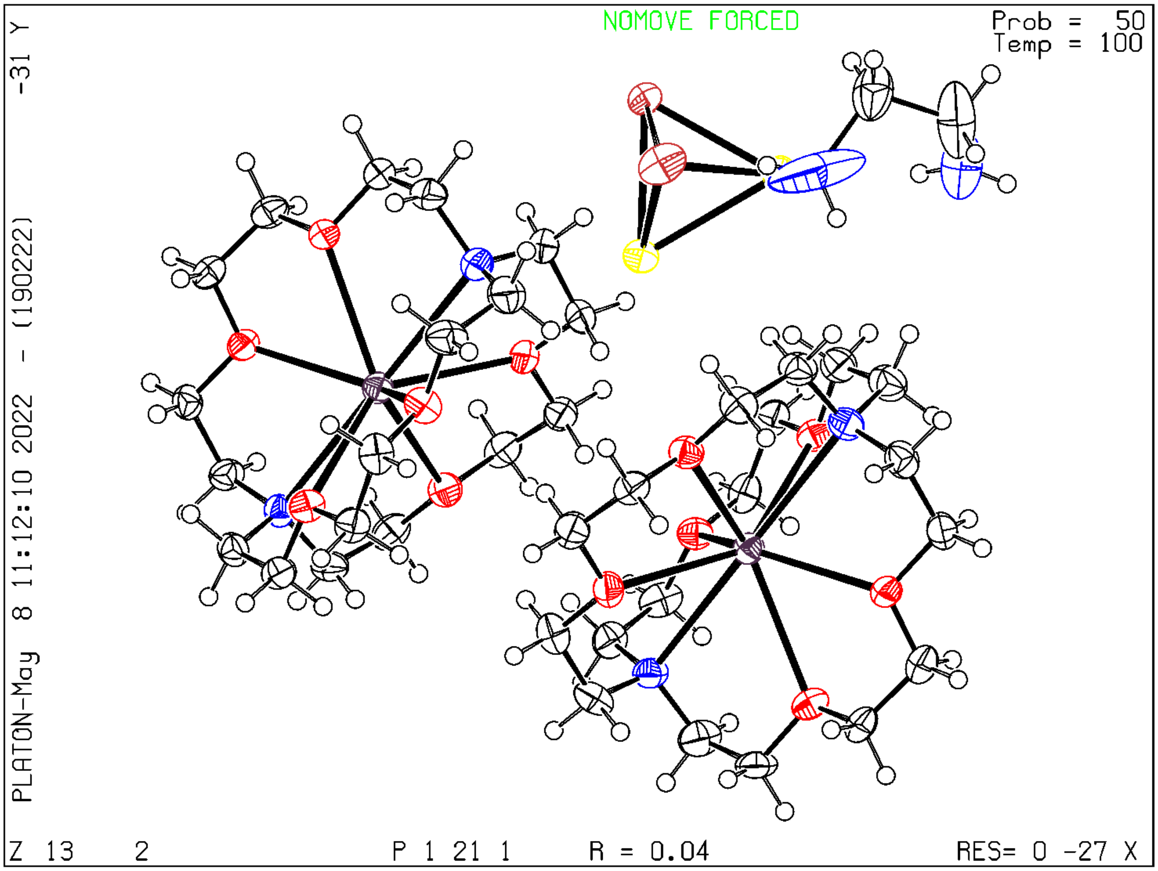

Supplement: Supplementary file 8 — Supporting Information [file ANIE-61-0-s009.pdf]
