# Supplementary material for: Between Elemental Match and Mismatch: From K12Ge3.5Sb6 to Salts of (Ge2Sb2)2−, (Ge4Sb12)4−, and (Ge4Sb14)4−
Source: Angew Chem Int Ed Engl. 2022 Jul 28;61(41):e202207232. doi: 10.1002/anie.202207232 (PMC9796001; doi:10.1002/anie.202207232)

checkCIF/PLATON report

Structure factors have been supplied for datablock(s) 16-2neu

THIS REPORT IS FOR GUIDANCE ONLY. IF USED AS PART OF A REVIEW PROCEDURE FOR PUBLICATION, IT SHOULD NOT REPLACE THE EXPERTISE OF AN EXPERIENCED CRYSTALLOGRAPHIC REFEREE.

No syntax errors found. CIF dictionary Interpreting this report

Datablock: 16-2neu

Bond precision:	C-C = 0.0143 A		Wavelength=1.34143
Cell:	a=26.4406(6)	b=16.3553(5)	c=30.4115(7)
	alpha=90	beta=114.632(2)	gamma=90
Temperature:	150 K		

	Calculated	Reported
Volume	11954.6(6)	11954.5(6)
Space group	C 2/c	C 1 2/c 1
Hall group	-C 2yc	-C 2yc
Moiety formula	2(C18 H36 K N2 O6), 0.5(Ge2.80 Sb12), C2 H8 N2, 0.6(Ge)	Ge4 Sb8.6, 3.4(Sb), 4(C18 H36 K N2 O6), 2(C2 H8 N2)
Sum formula	C38 H80 Ge2 K2 N6 O12 Sb6	C76 H160 Ge4 K4 N12 O24 Sb12
Mr	1767.06	3533.91
Dx,g cm-3	1.964	1.964
Z	8	4
Mu (mm-1)	16.368	16.185
F000	6832.0	6832.0
F000'	6831.27	
h,k,lmax	34,21,40	34,21,40
Nref	14495	13877
Tmin,Tmax	0.483,0.615	0.005,0.051
Tmin'	0.307	

Correction method= # Reported T Limits: Tmin=0.005 Tmax=0.051
AbsCorr = MULTI-SCAN

Data completeness= 0.957 Theta(max)= 62.497

R(reflections)= 0.0600(9244) wR2(reflections)= 0.1892(13877)

S = 1.048 Npar= 609

The following ALERTS were generated. Each ALERT has the format
test-name_ALERT_alert-type_alert-level.
Click on the hyperlinks for more details of the test.

Alert level A

PLAT308_ALERT_2_A Single Bonded Metal Atom in Structure (Unusual) Sb7 Check

Author Response: This alert is an error of the checkCIF software, which may have been caused by using PART commands to model disorder. Sb7 binds to its neigboring atoms at normal distances (2.575, 2.761, and 2.873 A, respectively).

PLAT308_ALERT_2_A Single Bonded Metal Atom in Structure (Unusual) Sb6 Check

Author Response: This alert is an error of the checkCIF software, which may have been caused by using PART commands to model disorder. Sb7 binds to its neigboring atoms at normal distances (2.575, 2.761, and 2.873 A, respectively).

Alert level C

```
PLAT029_ALERT_3_C _diffrn_measured_fraction_theta_full value Low .      0.973 Why?
PLAT051_ALERT_1_C Mu(calc) and Mu(CIF) Ratio Differs from 1.0 by .       1.13 %
PLAT230_ALERT_2_C Hirshfeld Test Diff for    C33      --C34      .        7.0 s.u.
PLAT234_ALERT_4_C Large Hirshfeld Difference O9       --C35      .       0.18 Ang.
PLAT234_ALERT_4_C Large Hirshfeld Difference N4       --C24      .       0.20 Ang.
PLAT234_ALERT_4_C Large Hirshfeld Difference C27      --C28      .       0.16 Ang.
PLAT234_ALERT_4_C Large Hirshfeld Difference C35      --C36      .       0.20 Ang.
PLAT241_ALERT_2_C High   'MainMol' Ueq as Compared to Neighbors of        C36 Check
PLAT241_ALERT_2_C High   'MainMol' Ueq as Compared to Neighbors of        Sb5 Check
PLAT342_ALERT_3_C Low Bond Precision on  C-C Bonds ...............    0.01426 Ang.
PLAT420_ALERT_2_C D-H Bond Without Acceptor  N5       --H5C      .     Please Check
PLAT420_ALERT_2_C D-H Bond Without Acceptor  N5       --H5D      .     Please Check
PLAT420_ALERT_2_C D-H Bond Without Acceptor  N6       --H6C      .     Please Check
PLAT420_ALERT_2_C D-H Bond Without Acceptor  N6       --H6D      .     Please Check
PLAT906_ALERT_3_C Large K Value in the Analysis of Variance ......      3.520 Check
PLAT911_ALERT_3_C Missing FCF Refl Between Thmin & STh/L=    0.600        290 Report
PLAT918_ALERT_3_C Reflection(s) with I(obs) much Smaller I(calc) .          2 Check
PLAT934_ALERT_3_C Number of (Iobs-Icalc)/Sigma(W) > 10 Outliers ..          1 Check
PLAT971_ALERT_2_C Check Calcd Resid. Dens.  0.64Ang From Sb6             2.42 eA-3
PLAT971_ALERT_2_C Check Calcd Resid. Dens.  0.92Ang From Ge4             2.21 eA-3
PLAT971_ALERT_2_C Check Calcd Resid. Dens.  0.60Ang From Ge5             2.20 eA-3
PLAT971_ALERT_2_C Check Calcd Resid. Dens.  0.42Ang From Sb6             1.66 eA-3
PLAT971_ALERT_2_C Check Calcd Resid. Dens.  0.87Ang From Sb1             1.54 eA-3
PLAT972_ALERT_2_C Check Calcd Resid. Dens.  0.21Ang From Sb2            -1.74 eA-3
```

Alert level G

```
ABSMU01_ALERT_1_G  Calculation of _exptl_absorpt_correction_mu
                not performed for this radiation type.
PLAT007_ALERT_5_G Number of Unrefined Donor-H Atoms .............          4 Report
PLAT042_ALERT_1_G Calc. and Reported Moiety Formula Strings Differ     Please Check
PLAT045_ALERT_1_G Calculated and Reported Z Differ by a Factor ...          2 Check
PLAT072_ALERT_2_G SHELXL First  Parameter in WGHT  Unusually Large       0.12 Report
PLAT168_ALERT_4_G The CIF-Embedded .res File Contains EXYZ Records          4 Report
PLAT171_ALERT_4_G The CIF-Embedded .res File Contains EADP Records          4 Report
PLAT230_ALERT_2_G Hirshfeld Test Diff for    Ge1      --Ge2      .       11.5 s.u.
PLAT230_ALERT_2_G Hirshfeld Test Diff for    Ge1      --Ge3_a    .       12.5 s.u.
PLAT230_ALERT_2_G Hirshfeld Test Diff for    Ge3      --Ge1_a    .       12.5 s.u.
PLAT232_ALERT_2_G Hirshfeld Test Diff (M-X)  Sb1      --Ge1      .       11.5 s.u.
PLAT232_ALERT_2_G Hirshfeld Test Diff (M-X)  Sb2      --Ge1_a    .        9.7 s.u.
PLAT232_ALERT_2_G Hirshfeld Test Diff (M-X)  Sb2      --Ge4_a    .       15.2 s.u.
PLAT232_ALERT_2_G Hirshfeld Test Diff (M-X)  Sb3      --Ge2      .       14.0 s.u.
PLAT232_ALERT_2_G Hirshfeld Test Diff (M-X)  Sb3      --Ge4      .       15.0 s.u.
PLAT232_ALERT_2_G Hirshfeld Test Diff (M-X)  Sb5      --Ge4      .        9.3 s.u.
PLAT232_ALERT_2_G Hirshfeld Test Diff (M-X)  Sb8      --Ge1      .       10.5 s.u.
PLAT232_ALERT_2_G Hirshfeld Test Diff (M-X)  Sb9      --Ge1_a    .       12.5 s.u.
PLAT300_ALERT_4_G Atom Site Occupancy of Sb1        Constrained at        0.9 Check
PLAT300_ALERT_4_G Atom Site Occupancy of Sb4        Constrained at        0.8 Check
PLAT300_ALERT_4_G Atom Site Occupancy of Sb7        Constrained at        0.5 Check
PLAT300_ALERT_4_G Atom Site Occupancy of Sb8        Constrained at        0.5 Check
PLAT300_ALERT_4_G Atom Site Occupancy of Sb6        Constrained at        0.4 Check
PLAT300_ALERT_4_G Atom Site Occupancy of Sb9        Constrained at        0.4 Check
PLAT300_ALERT_4_G Atom Site Occupancy of Ge2        Constrained at        0.1 Check
PLAT300_ALERT_4_G Atom Site Occupancy of Ge3        Constrained at        0.1 Check
PLAT300_ALERT_4_G Atom Site Occupancy of Ge4        Constrained at        0.2 Check
PLAT300_ALERT_4_G Atom Site Occupancy of Ge5        Constrained at        0.6 Check
PLAT302_ALERT_4_G Anion/Solvent/Minor-Residue Disorder (Resd  3  )        53% Note
PLAT302_ALERT_4_G Anion/Solvent/Minor-Residue Disorder (Resd  5  )       100% Note
PLAT304_ALERT_4_G Non-Integer Number of Atoms in ..... (Resd  3  )      14.80 Check
PLAT304_ALERT_4_G Non-Integer Number of Atoms in ..... (Resd  5  )       0.60 Check
PLAT720_ALERT_4_G Number of Unusual/Non-Standard Labels ..........          1 Note
PLAT779_ALERT_4_G Suspect or Irrelevant (Bond) Angle(s) in CIF ...      21.31 Deg.
              SB8  -GE1  -GE3     1_555   1_555   2_656 ......     #   10 Check
PLAT779_ALERT_4_G Suspect or Irrelevant (Bond) Angle(s) in CIF ...      19.57 Deg.
              GE3  -GE5  -SB8     1_555   1_555   2_656 ......     #   29 Check
PLAT779_ALERT_4_G Suspect or Irrelevant (Bond) Angle(s) in CIF ...      41.13 Deg.
              SB8  -GE3  -SB7     2_656   1_555   1_555 ......     #   55 Check
PLAT780_ALERT_1_G Coordinates do not Form a Properly Connected Set     Please Do !
PLAT790_ALERT_4_G Centre of Gravity not Within Unit Cell: Resd.  #          3 Note
              Ge2.80 Sb12
PLAT790_ALERT_4_G Centre of Gravity not Within Unit Cell: Resd.  #          4 Note
              C2 H8 N2
PLAT883_ALERT_1_G No Info/Value for _atom_sites_solution_primary .     Please Do !
PLAT912_ALERT_4_G Missing # of FCF Reflections Above STh/L=  0.600        328 Note
PLAT913_ALERT_3_G Missing # of Very Strong Reflections in FCF ....          1 Note
PLAT933_ALERT_2_G Number of HKL-OMIT Records in Embedded .res File         11 Note
PLAT941_ALERT_3_G Average HKL Measurement Multiplicity ...........        2.6 Low
PLAT978_ALERT_2_G Number C-C Bonds with Positive Residual Density.          0 Info
PLAT984_ALERT_1_G The  C-f’=    0.0148 Deviates from the B&C-Value     0.0137 Check
PLAT984_ALERT_1_G The Ge-f’=   -1.5486 Deviates from the B&C-Value    -1.5187 Check
PLAT984_ALERT_1_G The  K-f’=    0.3891 Deviates from the B&C-Value     0.3778 Check
PLAT984_ALERT_1_G The  N-f’=    0.0253 Deviates from the B&C-Value     0.0241 Check
PLAT984_ALERT_1_G The  O-f’=    0.0412 Deviates from the B&C-Value     0.0389 Check
```

```
PLAT984_ALERT_1_G The Sb-f'=    0.0887 Deviates from the B&C-Value     0.1003 Check
PLAT985_ALERT_1_G The Ge-f"=    0.7176 Deviates from the B&C-Value     0.6907 Check
PLAT985_ALERT_1_G The  K-f"=    0.8535 Deviates from the B&C-Value     0.8310 Check
PLAT985_ALERT_1_G The Sb-f"=    4.4827 Deviates from the B&C-Value     4.6759 Check
```

```
   2 ALERT level A = Most likely a serious problem - resolve or explain
   0 ALERT level B = A potentially serious problem, consider carefully
  24 ALERT level C = Check. Ensure it is not caused by an omission or oversight
  54 ALERT level G = General information/check it is not something unexpected

  15 ALERT type 1 CIF construction/syntax error, inconsistent or missing data
  29 ALERT type 2 Indicator that the structure model may be wrong or deficient
   8 ALERT type 3 Indicator that the structure quality may be low
  27 ALERT type 4 Improvement, methodology, query or suggestion
   1 ALERT type 5 Informative message, check
```

It is advisable to attempt to resolve as many as possible of the alerts in all categories. Often the minor alerts point to easily fixed oversights, errors and omissions in your CIF or refinement strategy, so attention to these fine details can be worthwhile. In order to resolve some of the more serious problems it may be necessary to carry out additional measurements or structure refinements. However, the purpose of your study may justify the reported deviations and the more serious of these should normally be commented upon in the discussion or experimental section of a paper or in the "special_details" fields of the CIF. checkCIF was carefully designed to identify outliers and unusual parameters, but every test has its limitations and alerts that are not important in a particular case may appear. Conversely, the absence of alerts does not guarantee there are no aspects of the results needing attention. It is up to the individual to critically assess their own results and, if necessary, seek expert advice.

Publication of your CIF in IUCr journals

A basic structural check has been run on your CIF. These basic checks will be run on all CIFs submitted for publication in IUCr journals (*Acta Crystallographica*, *Journal of Applied Crystallography*, *Journal of Synchrotron Radiation*); however, if you intend to submit to *Acta Crystallographica Section C* or *E* or *IUCrData*, you should make sure that full publication checks are run on the final version of your CIF prior to submission.

Publication of your CIF in other journals

Please refer to the *Notes for Authors* of the relevant journal for any special instructions relating to CIF submission.

PLATON version of 19/02/2022; check.def file version of 19/02/2022

Datablock 16-2neu - ellipsoid plot

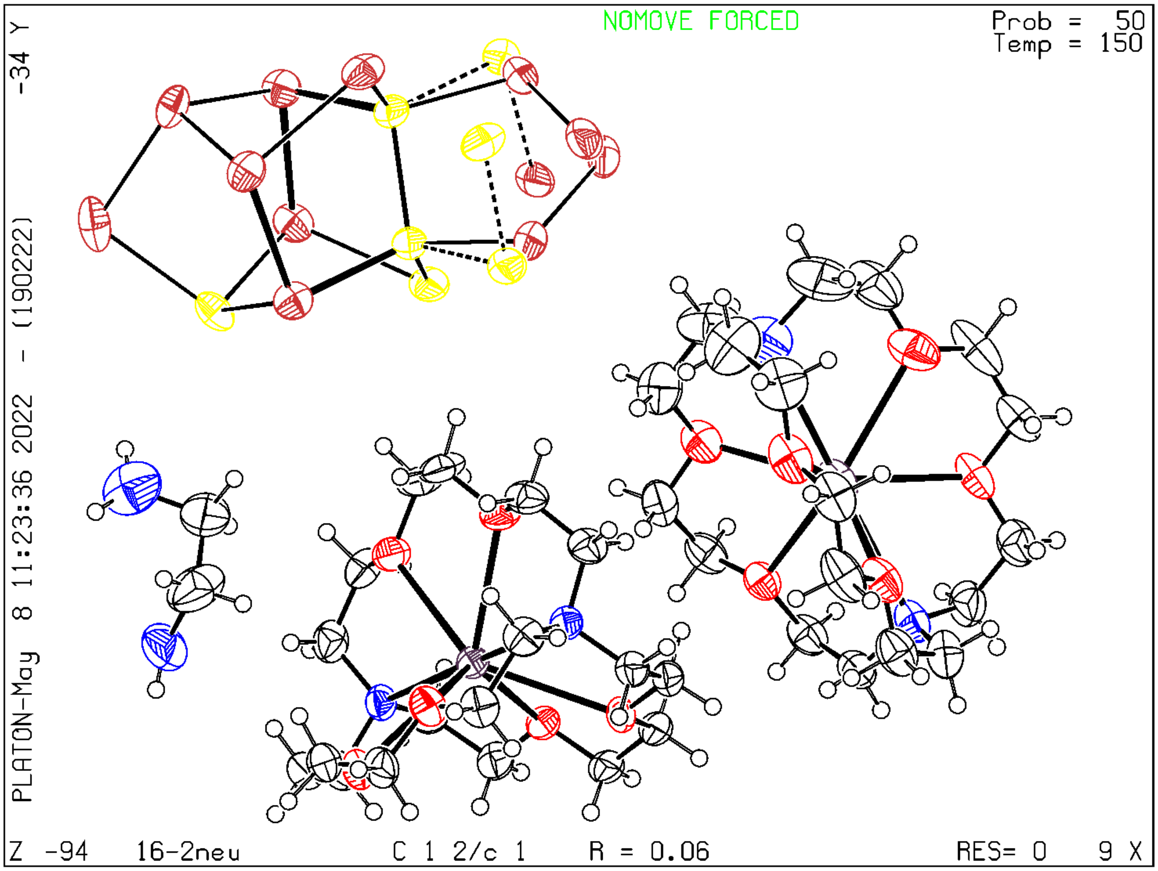

Supplement: Supplementary file 9 — Supporting Information [file ANIE-61-0-s008.pdf]
