# Supplementary material for: Between Elemental Match and Mismatch: From K12Ge3.5Sb6 to Salts of (Ge2Sb2)2−, (Ge4Sb12)4−, and (Ge4Sb14)4−
Source: Angew Chem Int Ed Engl. 2022 Jul 28;61(41):e202207232. doi: 10.1002/anie.202207232 (PMC9796001; doi:10.1002/anie.202207232)

checkCIF/PLATON report

Structure factors have been supplied for datablock(s) 2

THIS REPORT IS FOR GUIDANCE ONLY. IF USED AS PART OF A REVIEW PROCEDURE FOR PUBLICATION, IT SHOULD NOT REPLACE THE EXPERTISE OF AN EXPERIENCED CRYSTALLOGRAPHIC REFEREE.

No syntax errors found. CIF dictionary Interpreting this report

Datablock: 2

Bond precision: C-C = 0.0254 A Wavelength=0.71073

Cell:	a=25.2845(6)	b=17.6555(6)	c=28.4760(7)
	alpha=90	beta=90	gamma=90
Temperature:	100 K		

	Calculated	Reported
Volume	12712.0(6)	12712.0(6)
Space group	P b c a	P b c a
Hall group	-P 2ac 2ab	-P 2ac 2ab
Moiety formula	Ge4 Sb14, 4(C18 H36 K N2 O6) [+ solvent]	Ge4 Sb14, 4(C18 H36 K N2 O6)
Sum formula	C72 H144 Ge4 K4 N8 O24 Sb14 [+ solvent]	C72 H144 Ge4 K4 N8 O24 Sb14
Mr	3657.43	3657.20
Dx,g cm-3	1.911	1.911
Z	4	4
Mu (mm-1)	4.036	4.036
F000	6968.0	6968.0
F000'	6943.07	
h,k,lmax	30,21,34	30,21,34
Nref	11837	11809
Tmin,Tmax	0.157,0.298	0.157,0.298
Tmin'	0.122	

Correction method= # Reported T Limits: Tmin=0.157 Tmax=0.298
AbsCorr = NUMERICAL

Data completeness= 0.998 Theta(max)= 25.499

R(reflections)= 0.0795(5245)
wR2(reflections)= 0.2473(11809)

S = 0.906 Npar= 568

The following ALERTS were generated. Each ALERT has the format
test-name_ALERT_alert-type_alert-level.
Click on the hyperlinks for more details of the test.

Alert level B

PLAT242_ALERT_2_B Low 'MainMol' Ueq as Compared to Neighbors of K2 Check

Author Response: This is due to inherently low crystal quality.

PLAT342_ALERT_3_B Low Bond Precision on C-C Bonds .... 0.02539 Ang.

Author Response: This is due to inherently low crystal quality.

Alert level C

ABSTY02_ALERT_1_C An _exptl_absorpt_correction_type has been given without
a literature citation. This should be contained in the
_exptl_absorpt_process_details field.
Absorption correction given as numerical
RINTA01_ALERT_3_C The value of Rint is greater than 0.12
Rint given 0.123
PLAT020_ALERT_3_C The Value of Rint is Greater Than 0.12 .... 0.123 Report
PLAT026_ALERT_3_C Ratio Observed / Unique Reflections (too) Low .. 44% Check
PLAT241_ALERT_2_C High 'MainMol' Ueq as Compared to Neighbors of O9 Check
PLAT241_ALERT_2_C High 'MainMol' Ueq as Compared to Neighbors of O10 Check
PLAT241_ALERT_2_C High 'MainMol' Ueq as Compared to Neighbors of C1A Check
PLAT241_ALERT_2_C High 'MainMol' Ueq as Compared to Neighbors of C1B Check
PLAT241_ALERT_2_C High 'MainMol' Ueq as Compared to Neighbors of C1D Check
PLAT241_ALERT_2_C High 'MainMol' Ueq as Compared to Neighbors of C1F Check
PLAT241_ALERT_2_C High 'MainMol' Ueq as Compared to Neighbors of C1H Check
PLAT241_ALERT_2_C High 'MainMol' Ueq as Compared to Neighbors of C5 Check
PLAT241_ALERT_2_C High 'MainMol' Ueq as Compared to Neighbors of C7 Check
PLAT241_ALERT_2_C High 'MainMol' Ueq as Compared to Neighbors of C13 Check
PLAT241_ALERT_2_C High 'MainMol' Ueq as Compared to Neighbors of C14 Check
PLAT241_ALERT_2_C High 'MainMol' Ueq as Compared to Neighbors of C24 Check
PLAT241_ALERT_2_C High 'MainMol' Ueq as Compared to Neighbors of C27 Check
PLAT242_ALERT_2_C Low 'MainMol' Ueq as Compared to Neighbors of C9 Check

Author Response: This is due to inherently low crystal quality.

PLAT242_ALERT_2_C Low 'MainMol' Ueq as Compared to Neighbors of K1 Check

Author Response: This is due to inherently low crystal quality.

```
PLAT242_ALERT_2_C Low    'MainMol' Ueq as Compared to Neighbors of         N8 Check
```

Author Response: This is due to inherently low crystal quality.

```
PLAT260_ALERT_2_C Large Average Ueq of Residue Including        K2      0.166 Check
PLAT260_ALERT_2_C Large Average Ueq of Residue Including        K1      0.111 Check
PLAT410_ALERT_2_C Short Intra H...H Contact  H1CB     ..H6A      .       1.98 Ang.
                                                  x,y,z  =     1_555 Check
PLAT410_ALERT_2_C Short Intra H...H Contact  H10A     ..H23A     .       1.99 Ang.
                                                  x,y,z  =     1_555 Check
PLAT906_ALERT_3_C Large K Value in the Analysis of Variance ......     10.273 Check
PLAT906_ALERT_3_C Large K Value in the Analysis of Variance ......      3.399 Check
PLAT911_ALERT_3_C Missing FCF Refl Between Thmin & STh/L=    0.600         28 Report
PLAT918_ALERT_3_C Reflection(s) with I(obs) much Smaller I(calc) .          3 Check
PLAT973_ALERT_2_C Check Calcd Positive Resid. Density on       Sb1       1.15 eA-3
PLAT977_ALERT_2_C Check Negative Difference Density on H10B      .      -0.33 eA-3
```

Alert level G

```
PLAT002_ALERT_2_G Number of Distance or Angle Restraints on AtSite         61 Note
PLAT003_ALERT_2_G Number of Uiso or Uij Restrained non-H Atoms ...         52 Report
PLAT066_ALERT_1_G Predicted and Reported Tmin&Tmax Range Identical          ? Check
PLAT072_ALERT_2_G SHELXL First  Parameter in WGHT  Unusually Large       0.15 Report
PLAT168_ALERT_4_G The CIF-Embedded .res File Contains EXYZ Records          7 Report
PLAT171_ALERT_4_G The CIF-Embedded .res File Contains EADP Records          7 Report
PLAT172_ALERT_4_G The CIF-Embedded .res File Contains DFIX Records         25 Report
PLAT173_ALERT_4_G The CIF-Embedded .res File Contains DANG Records         12 Report
PLAT176_ALERT_4_G The CIF-Embedded .res File Contains SADI Records         12 Report
PLAT178_ALERT_4_G The CIF-Embedded .res File Contains SIMU Records          2 Report
PLAT187_ALERT_4_G The CIF-Embedded .res File Contains RIGU Records          2 Report
PLAT300_ALERT_4_G Atom Site Occupancy of Sb1       Constrained at         0.9 Check
PLAT300_ALERT_4_G Atom Site Occupancy of Sb2       Constrained at         0.9 Check
PLAT300_ALERT_4_G Atom Site Occupancy of Sb3       Constrained at         0.9 Check
PLAT300_ALERT_4_G Atom Site Occupancy of Sb4       Constrained at         0.9 Check
PLAT300_ALERT_4_G Atom Site Occupancy of Sb6       Constrained at         0.9 Check
PLAT300_ALERT_4_G Atom Site Occupancy of Sb7       Constrained at         0.9 Check
PLAT300_ALERT_4_G Atom Site Occupancy of Sb8       Constrained at         0.6 Check
PLAT300_ALERT_4_G Atom Site Occupancy of Ge2       Constrained at         0.4 Check
PLAT300_ALERT_4_G Atom Site Occupancy of Ge3       Constrained at         0.1 Check
PLAT300_ALERT_4_G Atom Site Occupancy of Ge4       Constrained at         0.1 Check
PLAT300_ALERT_4_G Atom Site Occupancy of Ge5       Constrained at         0.1 Check
PLAT300_ALERT_4_G Atom Site Occupancy of Ge6       Constrained at         0.1 Check
PLAT300_ALERT_4_G Atom Site Occupancy of Ge8       Constrained at         0.1 Check
PLAT300_ALERT_4_G Atom Site Occupancy of Ge9       Constrained at         0.1 Check
PLAT301_ALERT_3_G Main Residue  Disorder .............(Resd  1  )        78% Note
PLAT605_ALERT_4_G Largest Solvent Accessible VOID in the Structure        197 A**3
PLAT720_ALERT_4_G Number of Unusual/Non-Standard Labels ..........         18 Note
PLAT790_ALERT_4_G Centre of Gravity not Within Unit Cell: Resd.  #          2 Note
              C18 H36 K N2 O6
PLAT860_ALERT_3_G Number of Least-Squares Restraints ............        1189 Note
PLAT933_ALERT_2_G Number of HKL-OMIT Records in Embedded .res File         28 Note
PLAT961_ALERT_5_G Dataset Contains no Negative Intensities .......     Please Check
PLAT967_ALERT_5_G Note: Two-Theta Cutoff Value in Embedded .res ..       51.0 Degree
PLAT978_ALERT_2_G Number C-C Bonds with Positive Residual Density.          0 Info
```

```
 0 ALERT level A = Most likely a serious problem - resolve or explain
 2 ALERT level B = A potentially serious problem, consider carefully
30 ALERT level C = Check. Ensure it is not caused by an omission or oversight
34 ALERT level G = General information/check it is not something unexpected

 2 ALERT type 1 CIF construction/syntax error, inconsistent or missing data
28 ALERT type 2 Indicator that the structure model may be wrong or deficient
10 ALERT type 3 Indicator that the structure quality may be low
24 ALERT type 4 Improvement, methodology, query or suggestion
 2 ALERT type 5 Informative message, check
```

It is advisable to attempt to resolve as many as possible of the alerts in all categories. Often the minor alerts point to easily fixed oversights, errors and omissions in your CIF or refinement strategy, so attention to these fine details can be worthwhile. In order to resolve some of the more serious problems it may be necessary to carry out additional measurements or structure refinements. However, the purpose of your study may justify the reported deviations and the more serious of these should normally be commented upon in the discussion or experimental section of a paper or in the "special_details" fields of the CIF. checkCIF was carefully designed to identify outliers and unusual parameters, but every test has its limitations and alerts that are not important in a particular case may appear. Conversely, the absence of alerts does not guarantee there are no aspects of the results needing attention. It is up to the individual to critically assess their own results and, if necessary, seek expert advice.

Publication of your CIF in IUCr journals

A basic structural check has been run on your CIF. These basic checks will be run on all CIFs submitted for publication in IUCr journals (*Acta Crystallographica*, *Journal of Applied Crystallography*, *Journal of Synchrotron Radiation*); however, if you intend to submit to *Acta Crystallographica Section C* or *E* or *IUCrData*, you should make sure that full publication checks are run on the final version of your CIF prior to submission.

Publication of your CIF in other journals

Please refer to the *Notes for Authors* of the relevant journal for any special instructions relating to CIF submission.

PLATON version of 19/02/2022; check.def file version of 19/02/2022

Datablock 2 - ellipsoid plot

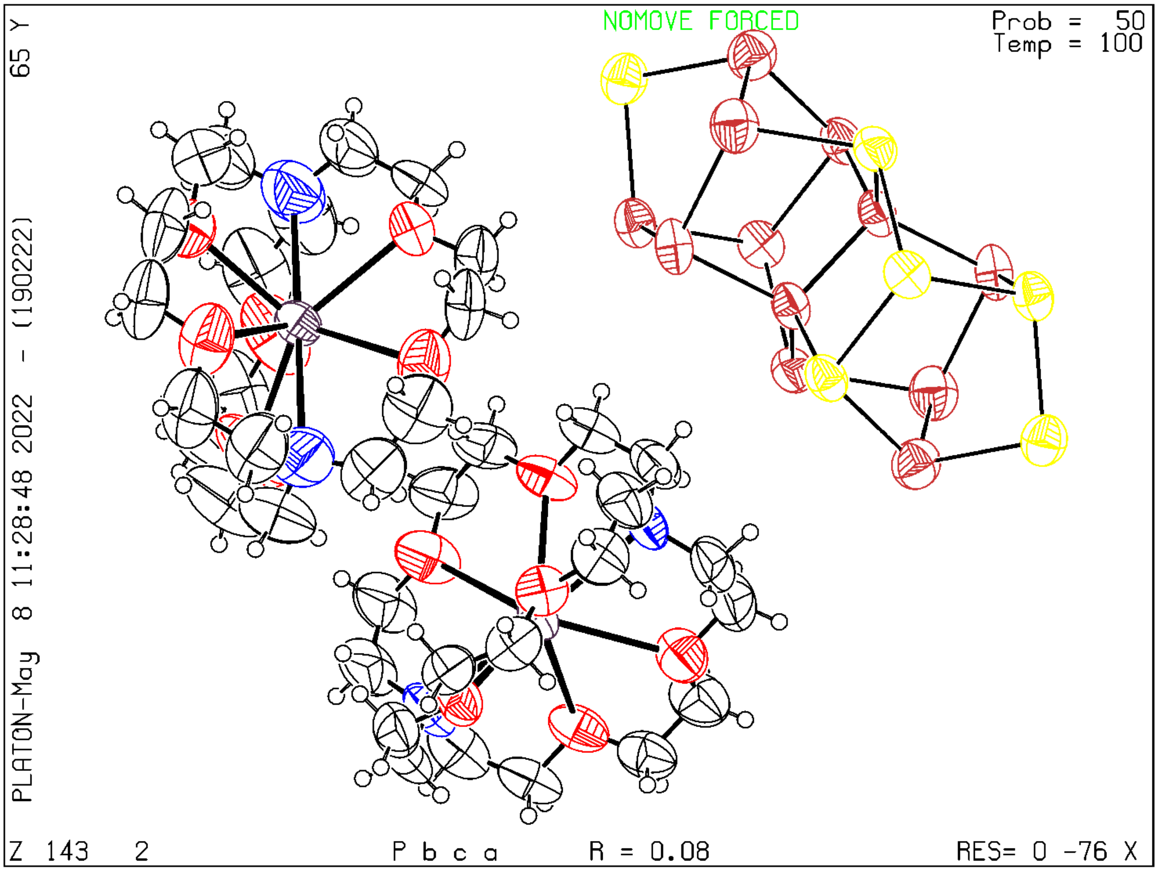

Supplement: Supplementary file 10 — Supporting Information [file ANIE-61-0-s004.pdf]
