# Supplementary material for: Between Elemental Match and Mismatch: From K12Ge3.5Sb6 to Salts of (Ge2Sb2)2−, (Ge4Sb12)4−, and (Ge4Sb14)4−
Source: Angew Chem Int Ed Engl. 2022 Jul 28;61(41):e202207232. doi: 10.1002/anie.202207232 (PMC9796001; doi:10.1002/anie.202207232)
Supplement: Supplementary file 11 — Supporting Information [file ANIE-61-0-s010.pdf]

Angewandte Chemie
Eine Zeitschrift der Gesellschaft Deutscher Chemiker

Supporting Information

Between Elemental Match and Mismatch: From $K_{12}Ge_{3.5}Sb_6$ to Salts of $(Ge_2Sb_2)^{2-}$, $(Ge_4Sb_{12})^{4-}$, and $(Ge_4Sb_{14})^{4-}$

*K. Beuthert, F. Pan, L. Guggolz, R. J. Wilson, J. Hempelmann, R. Dronskowski, S. Dehnen**

Contents:

1. Synthesis details 3

1.1 General 3

1.2 Synthesis of compounds 3

1.2.1 Synthesis of $K_{12}Ge_{3.5}Sb_6$ (1) 3

1.2.2 Synthesis of $[K(crypt\text{-}222)]_2(Ge_2Sb_2)\cdot en$ (2) and $[K(crypt\text{-}222)]_4(Ge_4Sb_{12})\cdot 2en$ (3) 3

1.2.3 Synthesis of $[K(crypt\text{-}222)]_4(Ge_4Sb_{14})$ (4) 3

2. Powder X-ray diffraction (PXRD) of $K_{12}Ge_{3.5}Sb_6$ (1) 4

3. Single crystal X-ray crystallography (SCXRD) 5

3.1 General considerations 5

3.2 Structure details 8

3.2.1 Structure details of $K_{12}Ge_4Sb_6$ (1) 8

3.2.2 Structure details of $[K(crypt\text{-}222)]_2(Ge_2Sb_2)\cdot en$ (2) 9

3.2.4 Structure details of $[K(crypt\text{-}222)]_4(Ge_4Sb_{12})\cdot 2en$ (3) 10

3.2.5 Structure details of $[K(crypt\text{-}222)]_4(Ge_4Sb_{14})$ (4) 13

4. Micro X-ray fluorescence spectroscopy (μ-XFS) analysis 16

4.2 Micro-X-ray fluorescence spectroscopy (μ-XFS) analysis of 1, 2 and 3 16

5. Electrospray ionization mass spectrometry (ESI-MS) investigations 19

5.1 Methods 19

5.1.1 Mass spectra of the reaction solution yielding 2 and 3 (5 min) 19

5.1.2 Mass spectra of the reaction solution yielding 2 and 3 (30 min) 21

5.1.3Mass spectra of the reaction solution yielding 2 and 3 (3 h) 22

5.1.4 Mass spectra of the reaction solution yielding 2 and 3 (1 d) 23

5.1.5 Mass spectra of the reaction solution yielding 2 and 3 (1w and 2w) 24

6. Quantum Chemical Investigations 25

6.1 Methods of the DFT Studies of the Solid State Compound 1 25

6.2 Methods of the DFT Studies of Molecular Anions 25

6.3 Details of the Quantum Chemical Investigations of Compound 1 25

6.4 Details of the Quantum Chemical Investigations of Compound 2 27

6.5 Details of the Quantum Chemical Investigations of Compound 3 28

6.6 Details of the Quantum Chemical Investigations of Compound 4 31

7. References for the Supporting Information 37

1. Synthesis details

1.1 General

All manipulations and reactions were performed in dry argon atmosphere using standard Schlenk or glovebox techniques. Ethane-1,2-diamine (en) was distilled from CaH_2 and stored over 3 Å molecular sieves. Toluene (Acros Organics, 99 %) was distilled from sodium-potassium alloy and stored over 3 Å molecular sieves. 4,7,13,16,21,24-hexaoxa-1,10-diazabicyclo[8.8.8]hexacosane (crypt-222,[1] Merck) was dried *in vacuo* for at least 18 hours. [$AuMePPh_3$] was commercially available from Aldrich.

1.2 Synthesis of compounds

1.2.1 Synthesis of $K_{12}Ge_{3.5}Sb_6$ (1)

$K_{12}Ge_{3.5}Sb_6$ was prepared by fusion of the elements (ratio: 2:1:1) at 950 °C for 48 hours in a niobium tube, sealed within an evacuated silica ampoule. The single-crystalline solid formed alongside some elemental Ge.

1.2.2 Synthesis of [K(crypt-222)]$_2$(Ge_2Sb_2)·en (2) and [K(crypt-222)]$_4$(Ge_4Sb_{12})·2en (3)

50 mg (0.183 mmol) of **1** and 139.49 mg (0.371 mmol) of crypt-222 were combined in a Schlenk tube and dissolved in en (5 mL). The reaction mixture was allowed to stir for 24 hours. The resulting intense orange solution was filtered through a standard glass frit. Because carefully layering with toluene (15 mL) and storing for crystallization was not successful, the solvent was evaporated until first crystals were visible. The Schlenk tube was then stored for further crystallization at 5 °C. After 1 day, crystals of compound **2** (orange sticks; >85 % of the crystals) and **3** (dark brown ellipsoids; >5 % of the crystals) formed in the Schlenk tube in approx. 70 % yield overall.

1.2.3 Synthesis of [K(crypt-222)]$_4$(Ge_4Sb_{14}) (4)

80 mg (0.293 mmol) of **1**, 221 mg (0.578 mmol) of crypt-222 and 37 mg (0.077 mmol) of [$AuMePPh_3$] were combined in a Schlenk tube and dissolved in en (3 mL). The reaction mixture was allowed to stir for 3 hours. The resulting dark brown solution was filtered through a standard glass frit, carefully layered with toluene (3 mL), and stored for crystallization at 5 °C. After 40 days, block-like metallic black crystals of compound **4** formed at the wall of the Schlenk tube in approx. 20 % yield.

2. Powder X-ray diffraction (PXRD) of $K_{12}Ge_{3.5}Sb_6$ (1)

Powder X-ray diffraction (PXRD) data were collected on a Stoe StadiMP diffractometer system equipped with a Mythen 1 K silicon strip detector and Cu-K_α-radiation (λ = 1.54056 Å). The sample of **1** was filled into a glass capillary (0.3 mm diameter), which was sealed air-tightly with soft wax. The tube was then mounted onto the goniometer head using wax (horizontal setup) and rotated throughout the measurement. The diffraction pattern is shown in Figure S1.

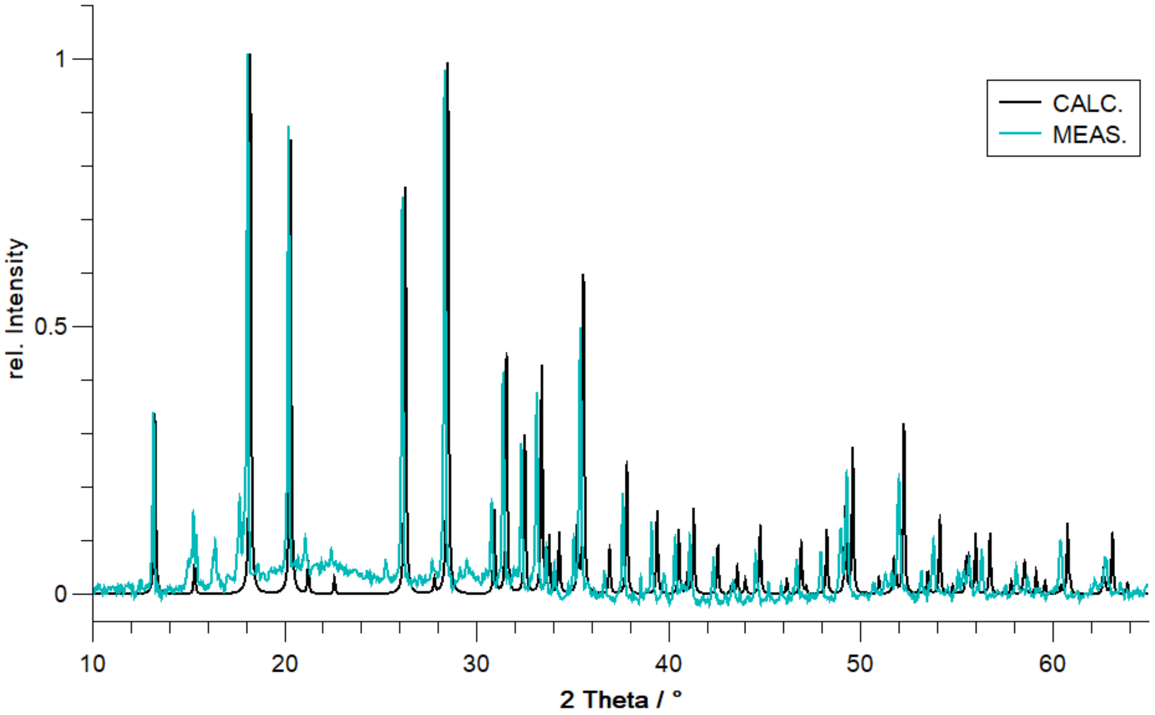

Figure S1. PXRD pattern of **1** for comparison as measured (green line) and calculated (black line) from the single-crystal CIF deposited as CCDC 2171370. The slight shift of the reflection positions is due to texture effects. With the aim to explain some additional (weak) reflections (e.g., at 16, 17.5 and 25.5, 29, 29.5, 38.5, 39.5, and 45.5°) we compared the PXRD pattern with all known diffraction patterns of solids comprising K, Nb, Ge, or Sb, or any combination of these elements (~170 compounds in the Pearson data base). However, there is no match; we thus assume that the additional reflections stem from at least one so far unidentified minor binary or ternary by-product that seems to be tightly attached to the single crystals we investigated.

3. Single crystal X-ray crystallography (SCXRD)

3.1 General considerations

The data for the X-ray structural analyses were collected at T = 100.0 K with Mo-Kα-radiation (λ = 0.71073 Å) on area detector systems Stoe IPDS/2T for **1**, **2** and **4**, Cu-Kα-radiation (λ = 1.54186 Å) on an area detector system Stoe StadiVari and T = 150.0 K with Ga-Kα-radiation (λ = 1.34143 Å) on a Stoe StadiVari diffractometer for **3**. The structures were solved by dual space methods of SHELXT from SHELXL-2018/136,[2] and refined by full matrix least-squares methods against F^2 with the SHELXL program.[3] All hydrogen atoms were kept riding on calculated positions with isotropic displacement parameters U = 1.2 Ueq of the bonding partners. The crystal quality of compound **4** was comparably poor. Zintl compounds, especially ones with highly symmetric clusters in them, tend to suffer from some inherent rotational disorder of the clusters, which can result in diffraction data such as the ones observed here. As we could not reproduce this compound so far, we could not collect a better data set. However, we were able to unambiguously determine the crystal structure from it. Crystallographic data for the structures of **1** – **4** (Table S1 – S6) reported in this paper have been deposited with the Cambridge Crystallographic Data Centre as supplementary publication no. CCDC-2171370 – CCDC-2171373. Copies of the data can be obtained free of charge on application to CCDC, 12 Union Road, Cambridge CB2 1EZ, UK [fax.: (internat.) + 44 1223/336-033; e-mail: deposit@ccdc.cam.ac.uk]. Supplementary structural figures (Figures S3 – S7) were created with Diamond 4.[4]

Table S1. Crystal data and details of the structure determinations of compound 1 and 2.

**Compound**	**1**	**2**
Empirical formula	$Ge_{0.29}KSb_{0.5}$	$C_{38}H_{80}Ge_2K_2N_6O_{12}Sb_2$
Formula weight [g mol^{-1}]	121.15	1279.96
Temperature [K]	100	100
Crystal system	hexagonal	monoclinic
Space group	$P6_3/m$	$P2_1$
a [Å]	13.3366(19)	11.9235(5)
b [Å]	13.3366(19)	20.4010(6)
c [Å]	5.3594(11)	12.4643(5)
α [°]	90	90
β [°]	90	118.271(3)
γ [°]	120	90
Volume [Å^3]	825.5(3)	2670.30(19)
Z	12	2
ρ_{calc} [g cm^{-3}]	2.924	1.592
μ [mm^{-1}]	9.467	2.329
F(000)	662	1300
Radiation	Mo–Kα (λ = 0.71073 Å)	Mo–Kα (λ = 0.71073 Å)
2Θ range for data collection [°]	3.526 to 53.406	3.71 to 58.44
Index ranges	–16 ≤ h ≤ 16, –16 ≤ k ≤ 15, –6 ≤ l ≤ 6	–15 ≤ h ≤ 16, –27 ≤ k ≤ 28, –17 ≤ l ≤ 17
Reflections collected	7288	47254
Independent reflections	650 [R_{int} = 0.1336, R_{sigma} = 0.0458]	14393 [R_{int} = 0.1151, R_{sigma} = 0.1067]
Data/restraints/parameters	650/0/25	14393/1/559
Goodness–of–fit on F^2	1.073	0.885
Final R indexes [$I \geq 2\sigma(I)$]	R_1 = 0.0355, wR_2 = 0.0738	R_1 = 0.0440, wR_2 = 0.0959
Final R indexes [all data]	R_1 = 0.0464, wR_2 = 0.0767	R_1 = 0.0757, wR_2 = 0.1030
Largest diff. peak/hole [e Å^{-3}]	1.4/–1.4	1.17/–0.93
Flack parameter [5]	–	0.012(14)
CCDC number	2171370	2171371

Table S2. Crystal data and details of the structure determinations of compound 3 and 4.

**Compound**	**3**	**4**
Empirical formula	$C_{76}H_{160}Ge_4K_4N_{12}O_{24}Sb_{12}$	$C_{72}H_{144}Ge_4K_4N_8O_{24}Sb_{14}$
Formula weight [g mol^{-1}]	3533.91	3657.2
Temperature [K]	293(2)	100
Crystal system	monoclinic	orthorhombic
Space group	*C*2/c	*P*bca
a [Å]	26.4406(6)	25.2845(6)
b [Å]	16.3553(5)	17.6555(6)
c [Å]	30.4115(7)	28.4760(7)
α [°]	90	90
β [°]	114.632(2)	90
γ [°]	90	90
Volume [Å^3]	11954.5(6)	12712.0(6)
Z	4	4
ρ_{calc} [g cm^{-}3]	1.964	1.911
μ [mm^{-1}]	16.185	4.036
F(000)	6832	6968
Radiation	Ga–Ka–Kα (λ = 1.34143 Å)	Mo–Kα (λ = 0.71073 Å)
2Θ range for data collection [°]	5.562 to 124.994	2.86 to 49.998
Index ranges	–31 ≤ h ≤ 16, –21 ≤ k ≤ 19, –31 ≤ l ≤ 40	–29 ≤ h ≤ 30, –20 ≤ k ≤ 20, –33 ≤ l ≤ 31
Reflections collected	36521	72060
Independent reflections	13877 [R_{int} = 0.0409, R_{sigma} = 0.0495]	11165 [R_{int} = 0.1165, R_{sigma} = 0.0734]
Data/restraints/parameters	13877/0/609	11165/1188/568
Goodness–of–fit on F^2	1.048	0.944
Final *R* indexes [$I \geq 2\sigma(I)$]	R_1 = 0.0600, wR_2 = 0.1793	R_1 = 0.0933, wR_2 = 0.2668
Final *R* indexes [all data]	R_1 = 0.0868, wR_2 = 0.1892	R_1 = 0.1598, wR_2 = 0.2978
Largest diff. peak/hole [e Å^{-3}]	1.4/–1.4	1.17/–0.93
CCDC number	2171372	2171373

3.2 Structure details

3.2.1 Structure details of $K_{12}Ge_4Sb_6$ (1)

Table S3. Selected interatomic distances (in Å) and bond angles (in degrees) of the experimental and optimized structures of the anion in 1. Geometry optimization of the anion in 1 was performed in point group *D*$_{3h}$.

Atom1	Atom2		Distance (exp)	Distance (calc)
Sb1	Ge1		2.5676(6)	2.56
**Atom1**	**Atom2**	**Atom3**	**Angles (exp)**	**Angles (calc)**
Sb1	Ge1	Sb1'	120.0	120.0
Sb1	Ge1	Sb1"	120.0	120.0
Sb1'	Ge1	Sb1"	120.0	120.0

'1-X,1-Y,1-Z; "+X,+Y,1+Z

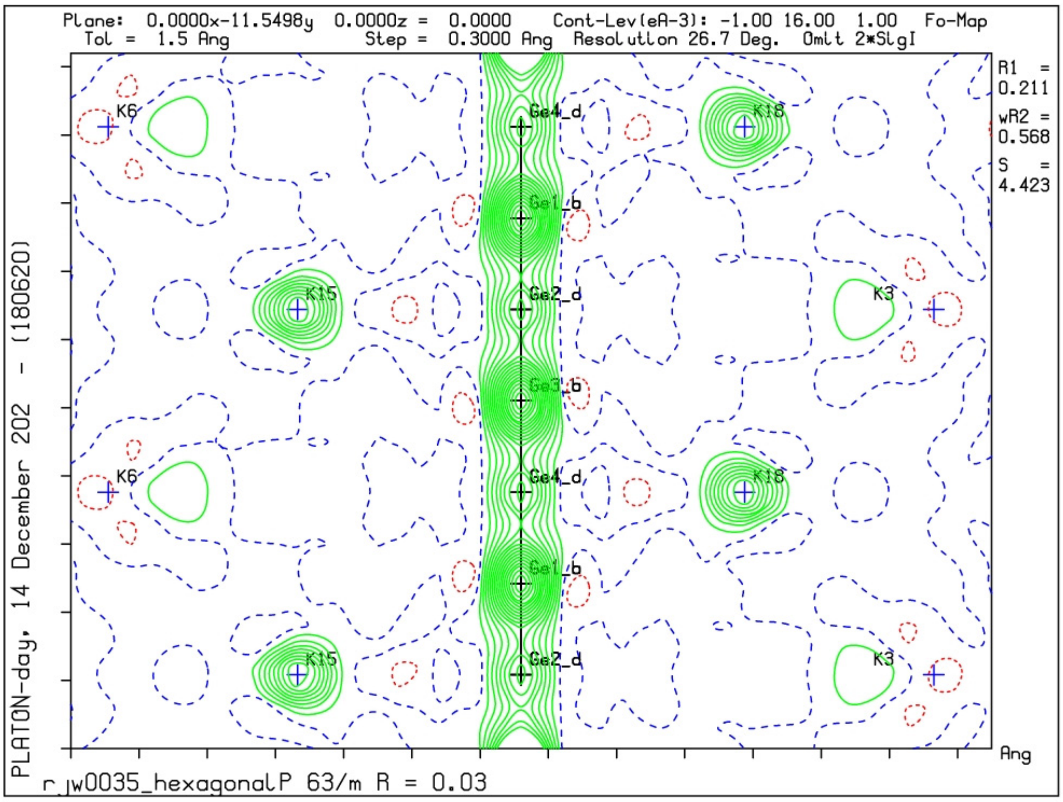

Figure S2. Fourier-Map of the Ge–Ge unit of compound **1**.

3.2.2 Structure details of $[K(crypt-222)]_2(Ge_2Sb_2)\cdot en$ (2)

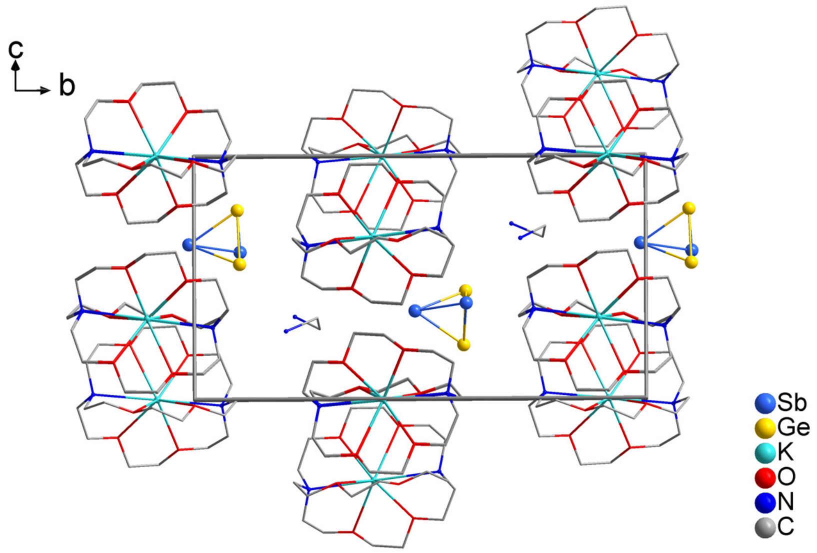

Figure S3. Unit cell of compound **2** viewed along the crystallographic *a* axis. H atoms have been omitted for clarity.

Table S4. Selected interatomic distances (in Å) and bond angles (in degrees) of the experimental and optimized structures of the anion in 2. Geometry optimization of the anion in 2 was performed in point group C_{2v}.

Atom1	Atom2		Distance (exp)	Distance (calc)
Sb1	Sb2		2.7889(8)	2.83
Sb1	Ge1		2.7057(10)	2.73
Sb1	Ge2		2.7013(12)	2.73
Sb2	Ge1		2.6945(9)	2.73
Sb2	Ge2		2.6943(9)	2.73
Ge1	Ge2		2.5317(11)	2.55
**Atom1**	**Atom2**	**Atom3**	**Angles (exp)**	**Angles (calc)**
Ge1	Sb1	Sb2	58.71(2)	58.82
Ge2	Sb1	Sb2	58.75(3)	58.82
Ge2	Sb1	Ge1	55.84(3)	55.56
Ge1	Sb2	Sb1	59.10(2)	58.82
Ge2	Sb2	Sb1	59.00(3)	58.82
Ge2	Sb2	Ge1	56.04(3)	55.56
Sb2	Ge1	Sb1	62.19(2)	62.36
Ge2	Ge1	Sb1	61.99(3)	62.22
Ge2	Ge1	Sb2	61.97(3)	62.22
Sb2	Ge2	Sb1	62.25(2)	62.36
Ge1	Ge2	Sb1	62.17(3)	62.22
Ge1	Ge2	Sb2	61.98(3)	62.22

3.2.4 Structure details of $[K(crypt\text{-}222)]_4(Ge_4Sb_{12})\cdot 2en$ (3)

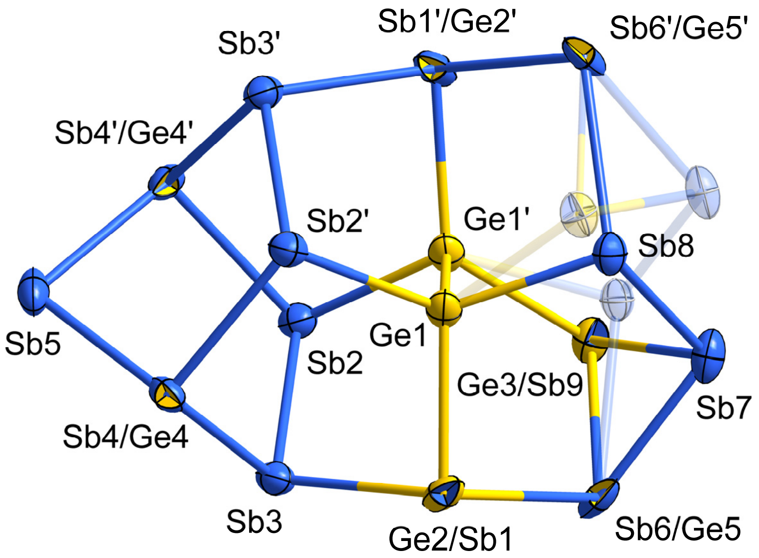

Figure S4. Molecular structure of the $(Ge_4Sb_{12})^{4-}$ anion in **3** with full labelling scheme and indication of the 50%:50% positional disorder of Sb7, Sb8, and Ge3/Sb9 (semi-transparent atoms and bonds). Thermal ellipsoids are shown at 30% probability.

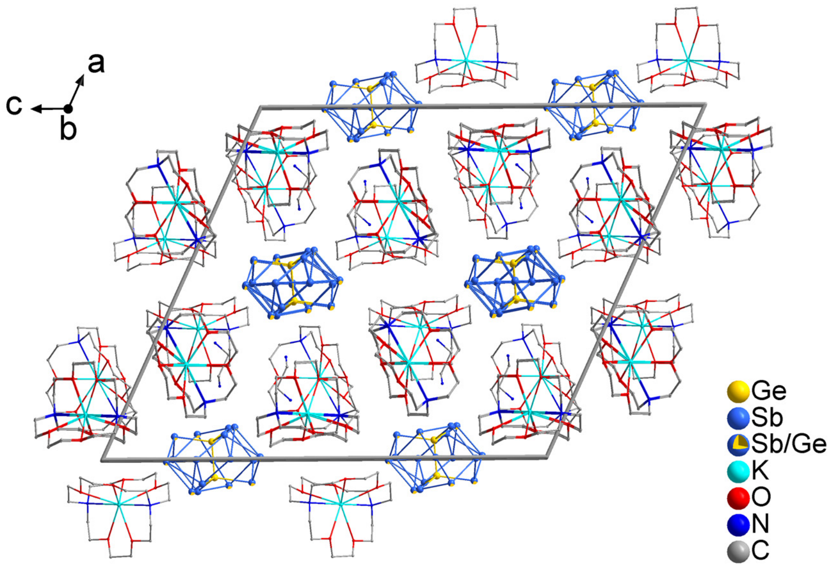

Figure S5. Unit cell of compound **3** viewed along the crystallographic *b* axis. H atoms have been omitted for clarity.

Table S5. Selected interatomic distances (in Å) and bond angles (in degrees) of the experimental and optimized structures of the anion in 3. The calculated bond lengths and angles are given for the energetically favored isomer I. Geometry optimization of the anion in 3 was performed without any symmetry restrictions.

Atom1	Atom2	Distance (exp)	Distance (calc)
Ge1	Ge1[1]	2.4538(17)	2.47
Ge1	Sb2[1]	2.5827(11)	2.64
Ge1	Ge2/Sb1	2.5822(11)	2.47
Ge1	Sb8	2.5214(14)	2.65
Ge1’	Ge3/Sb9	2.6682(16)	2.50
Sb5	Sb4’/Ge4’	2.7613(8)	2.79
Sb5	Sb4/Ge4	2.7612(8)	2.79
Sb2	Sb3	2.7908(7)	2.88
Sb2	Sb4’/Ge4’	2.7952(7)	2.88
Sb3	Sb4/Ge4	2.7874(8)	2.84
Sb3	Ge2/Sb1	2.8103(8)	2.73
Ge2/Sb1	Sb6/Ge5	2.7872(9)	2.75
Sb6/Ge5	Sb7	2.5758(17)	2.88
Sb6/Ge5	Ge3/Sb9	2.6789(14)	2.73
Sb7	Sb8	2.8732(18)	2.89
Sb7	Ge3/Sb9	2.7613(17)	2.73

Atom1	Atom2	Atom3	Angles (exp)	Angles (calc)
Ge1’	Ge1	Sb2’	109.29(3)	109.5
Ge1[1]	Ge1	Ge2/Sb1	101.72(6)	107.0
Ge1[1]	Ge1	Sb8	98.79(4)	97.1
Ge1	Ge1’	Ge3/Sb9	108.85(5)	109.9
Sb2	Ge1’	Ge3/Sb9	101.57(5)	107.2
Ge2/Sb1	Ge1	Sb2’	111.59(4)	114.9
Sb1’/Ge2’	Ge1’	Ge3/Sb9	123.50(5)	121.5
Sb8	Ge1	Sb2’	122.87(5)	110.4
Sb8	Ge1	Ge2/Sb1	109.75(5)	114.9
Sb4/Ge4	Sb5	Sb4’/Ge4’	95.89(3)	96.9
Ge1’	Sb2	Sb3	94.56(3)	92.5
Ge1[1]	Sb2	Sb4’/Ge4’	96.63(3)	100.7
Sb3	Sb2	Sb4’/Ge4’	105.86(2)	104.5
Sb2	Sb3	Ge2/Sb1	107.92(2)	110.3
Sb4/Ge4	Sb3	Sb2	100.25(2)	100.2
Sb4/Ge4	Sb3	Ge2/Sb1	94.04(2)	93.3

Table S5 (continued)

Atom1	Atom2	Atom3	Angles (exp)	Angles (calc)
Sb5	Sb4/Ge4	Sb2'	102.42(2)	102.9
Sb5	Sb4/Ge4	Sb3	100.88(2)	101.8
Sb3	Sb4/Ge4	Sb2'	103.49(2)	102.3
Ge1	Ge2/Sb1	Sb3	89.60(3)	88.7
Ge1	Ge2/Sb1	Sb6/Ge5	95.35(3)	90.9
Sb6/Ge5	Ge2/Sb1	Sb3	106.73(3)	101.9
Sb1'/Ge2'	Sb6'/Ge5'	Sb8	103.43(3)	104.5
Sb7	Sb6/Ge5	Ge2/Sb1	105.76(4)	105.0
Sb7	Sb6/Ge5	Ge3/Sb9	63.37(5)	58.3
Ge3/Sb9	Sb6/Ge5	Ge2/Sb1	108.36(4)	114.5
Sb6/Ge5	Sb7	Sb8	106.90(5)	104.6
Sb6/Ge5	Sb7	Ge3/Sb9	60.14(4)	58.2
Ge3/Sb9	Sb7	Sb8	105.36(5)	106.9
Ge1	Sb8	Sb6'/Ge5'	95.37(4)	98.8
Ge1	Sb8	Sb7	94.64(5)	89.5
Sb7	Sb8	Sb6'/Ge5'	108.22(5)	104.8
Ge1'	Ge3/Sb9	Sb6/Ge5	97.22(4)	92.3
Ge1'	Ge3/Sb9	Sb7	95.25(5)	95.6
Sb6/Ge5	Ge3/Sb9	Sb7	56.50(4)	63.6

'1-X,+Y,3/2-Z

3.2.5 Structure details of [K(crypt-222)]$_4$(Ge$_4$Sb$_{14}$) (4)

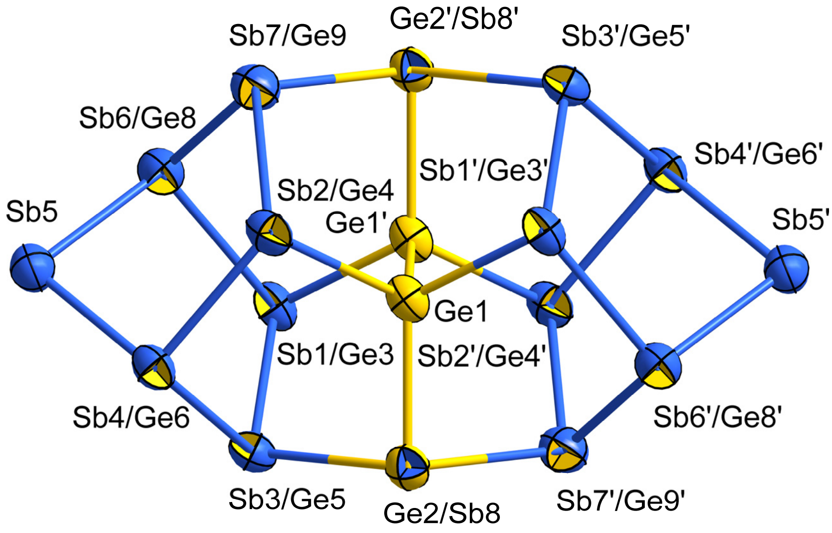

Figure S6. Molecular structure of the $(Ge_4Sb_{14})^{4-}$ anion in **4** with full labelling scheme. Thermal ellipsoids are shown at 30% probability.

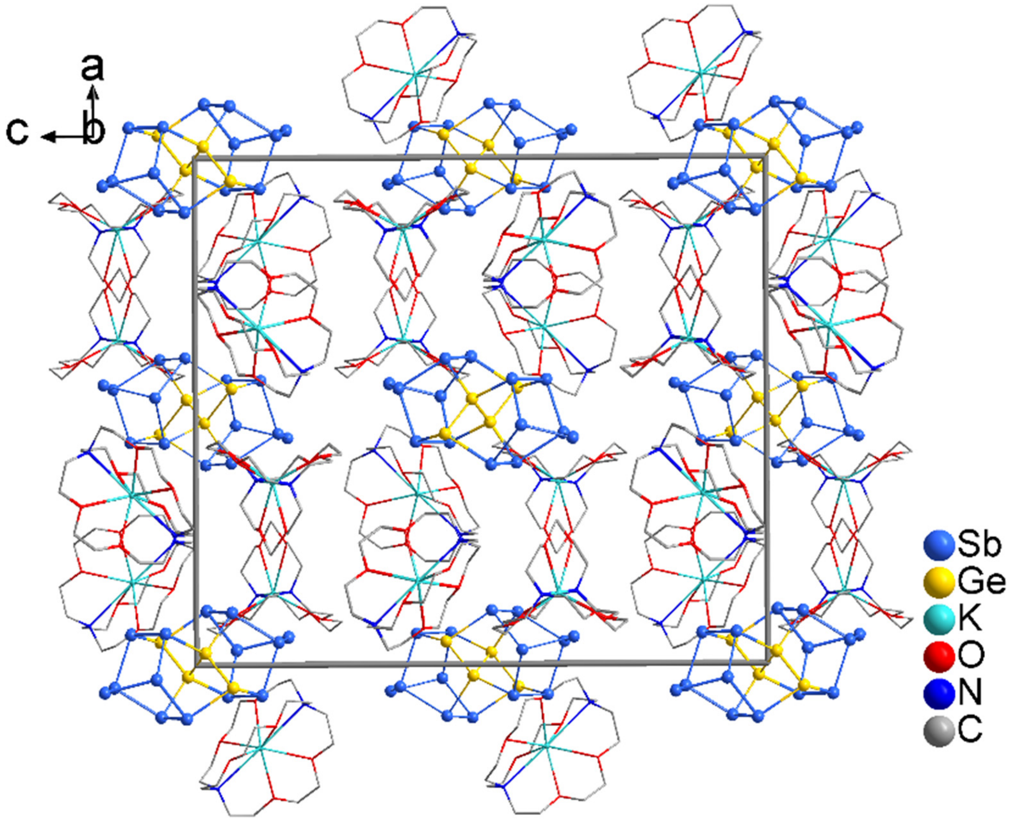

Figure S7. Unit cell of compound **4** viewed along the crystallographic *b* axis. H atoms have been omitted for clarity.

Table S6. Selected interatomic distances (in Å) and bond angles (in degrees) of the experimental and optimized structures of the anion in 4. Geometry optimization of the anion in 4 was performed in point group C_{2h}.

Atom1	Atom2		Distance (exp)	Distance (calc)
Sb1/Ge3	Sb3/Ge5		2.7785(19)	2.89
Sb1/Ge3	Sb6/Ge8		2.778(2)	2.86
Sb1/Ge3	Ge1'		2.582(2)	2.65
Sb3/Ge5	Sb4/Ge6		2.8129(19)	2.88
Sb3/Ge5	Ge2/Sb8		2.777(2)	2.71
Sb2/Ge4	Sb4/Ge6		2.8012(19)	2.86
Sb2/Ge4	Sb7/Ge9		2.761(2)	2.89
Sb2/Ge4	Ge1		2.563(2)	2.65
Sb4/Ge6	Sb5		2.758(2)	2.79
Sb5	Sb6/Ge8		2.784(2)	2.79
Ge2/Sb8	Sb7'/Ge9'		2.748(2)	2.71
Ge2/Sb8	Ge1		2.534(3)	2.46
Sb6/Ge8	Sb7/Ge9		2.795(2)	2.88
Ge1	Ge1'		2.425(4)	2.54
**Atom1**	**Atom2**	**Atom3**	**Angles (exp)**	**Angles (calc)**
Ge1	Ge2/Sb8	Sb3/Ge5	88.58(6)	89.4
Ge1	Ge2/Sb8	Sb7'/Ge9'	87.68(6)	89.4
Ge1	Sb2/Ge4	Sb4/Ge6	94.77(5)	92.1
Ge1	Sb2/Ge4	Sb7/Ge9	95.06(6)	91.9
Ge1'	Sb1/Ge3	Sb3/Ge5	96.55(5)	91.9
Ge1'	Sb1/Ge3	Sb6/Ge8	93.33(6)	92.1
Ge1'	Ge1	Ge2/Sb8	105.57(9)	108.7
Ge1'	Ge1	Sb1'/Ge3'	106.06(9)	106.0
Ge1'	Ge1	Sb2/Ge4	106.85(10)	106.0
Ge2/Sb8	Sb3/Ge5	Sb1/Ge3	107.20(5)	113.2
Ge2/Sb8	Sb3/Ge5	Sb4/Ge6	94.93(5)	93.7
Ge2'/Sb8'	Sb7/Ge9	Sb2/Ge4	109.25(6)	93.7
Ge2"/Sb8"	Sb7/Ge9	Sb6/Ge8	95.36(6)	113.2
Ge2/Sb8	Ge1	Sb1'/Ge3'	116.05(7)	114.3
Ge2/Sb8	Ge1	Sb2/Ge4	115.37(7)	114.3
Sb1/Ge3	Sb3/Ge5	Sb4/Ge6	98.75(5)	95.5
Sb1/Ge3	Sb6/Ge8	Sb7/Ge9	103.89(5)	102.7
Sb1/Ge3	Sb6/Ge8	Sb5	101.85(5)	98.9

Table S6 (continued)

Atom1	Atom2	Atom3	Angles (exp)	Angles (calc)
Sb2/Ge4	Sb4/Ge6	Sb3/Ge5	102.99(5)	102.7
Sb2/Ge4	Sb7/Ge9	Sb6/Ge8	97.92(5)	95.5
Sb2/Ge4	Ge1	Sb1'/Ge3'	106.22(6)	106.8
Sb3/Ge5	Sb1/Ge3	Sb6/Ge8	107.80(5)	110.7
Sb4/Ge6	Sb5	Sb6/Ge8	95.30(5)	97.3
Sb5	Sb4/Ge6	Sb3/Ge5	101.93(5)	105.7
Sb5	Sb4/Ge6	Sb2/Ge4	100.77(5)	98.9
Sb5	Sb6/Ge8	Sb7/Ge9	102.20(6)	105.7
Sb7/Ge9	Ge2'/Sb8'	Sb3'/Ge5'	114.89(6)	106.7
Sb7/Ge9	Sb2/Ge4	Sb4/Ge6	108.83(6)	110.7

'1-X,1-Y,-Z

4. Micro X-ray fluorescence spectroscopy (μ-XFS) analysis

4.2 Micro-X-ray fluorescence spectroscopy (μ-XFS) analysis of 1, 2 and 3

All μ-XFS measurements were performed on single crystal samples with a Bruker M4 Tornado, equipped with an Rh-target X-ray tube, poly capillary optics and a Si drift detector. The emitted fluorescence photons are detected with an acquisition time of 180 s. Quantification of the elements is achieved through deconvolution of the spectra. Results are summarized in Table S7. Figures S8–S10 show the spectra for **1** – **3** along with the results of the deconvolution algorithm. Several measurements produced unreasonably large values for the % K. Removal of K from the calculations afforded excellent agreement with the expected atomic ratio of close to $Ge_{2.00}Sb_{2.00}$ in **2**, and $Ge_{4.00}Sb_{12.00}$, respectively. It is a typical finding though for such compounds, the reasons for which could not be clarified so far. Measurements of compound **4** were not possible owing to the failure to reproduce it to date.

Table S7. μ-XFS analysis of 1, 2 and 3 (K, Ge, Sb).

		**1**		
Element	Element wt %	Atom %	Element ratio observed	Element ratio calc
K-K	33.78	56.78	13.8	12.00
Ge-K	20.47	18.53	4.5	3.50
Sb-L	45.75	24.69	6	6.00
Total	100.00	100.00		
		**2**		
Element	Element wt %	Atom %	Element ratio observed	Element ratio calc
K-K	24.20	44.64	3.45	2.00
Ge-K	26.07	25.90	2.00	2.00
Sb-L	49.47	29.46	2.27	2.00
Total	100.00	100.00		
		**3**		
Element	Element wt %	Atom %	Element ratio observed	Element ratio calc
K-K	11.27	26.27	5.67	4.00
Ge-L	14.42	18.10	3.90	4.00
Sb-L	74.31	55.63	12.00	12.00
Total	100.00	100.00		

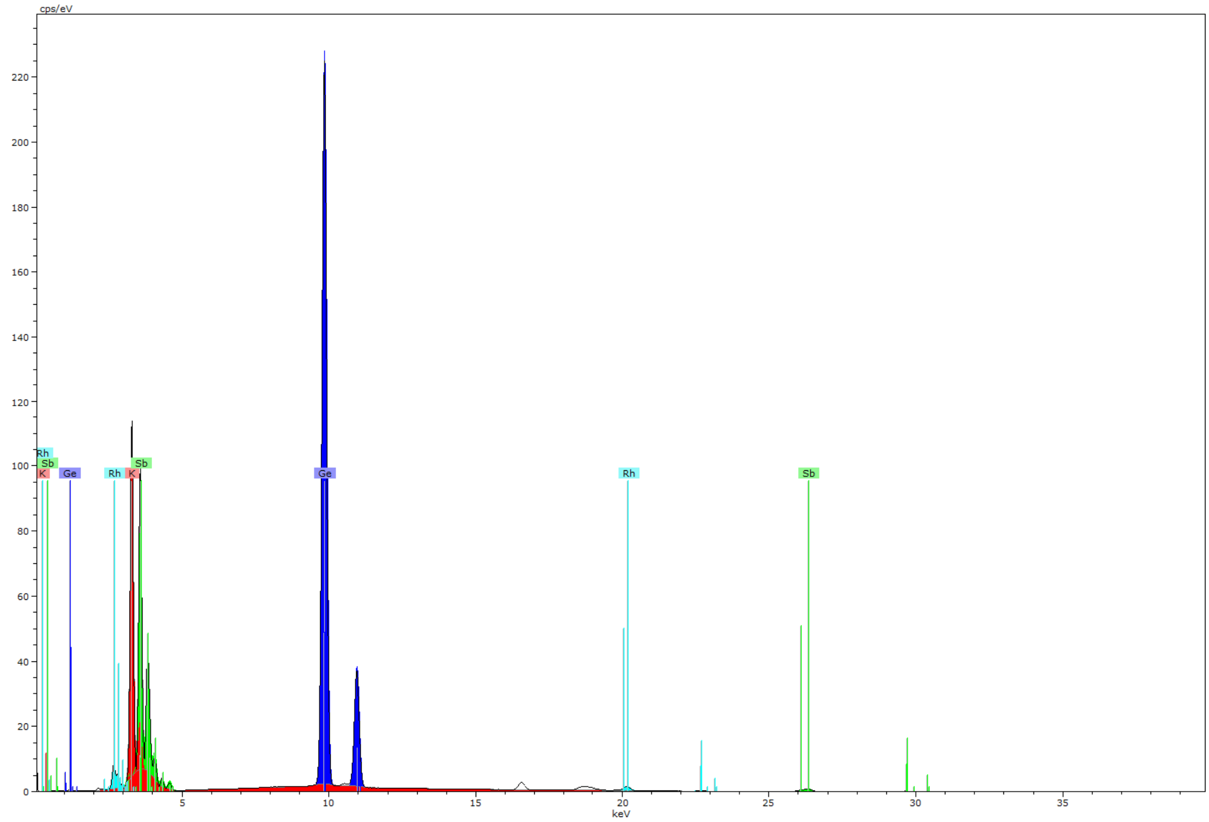

Figure S8. Micro X-ray fluorescence spectrum of **1** with the results of the deconvolution algorithm.

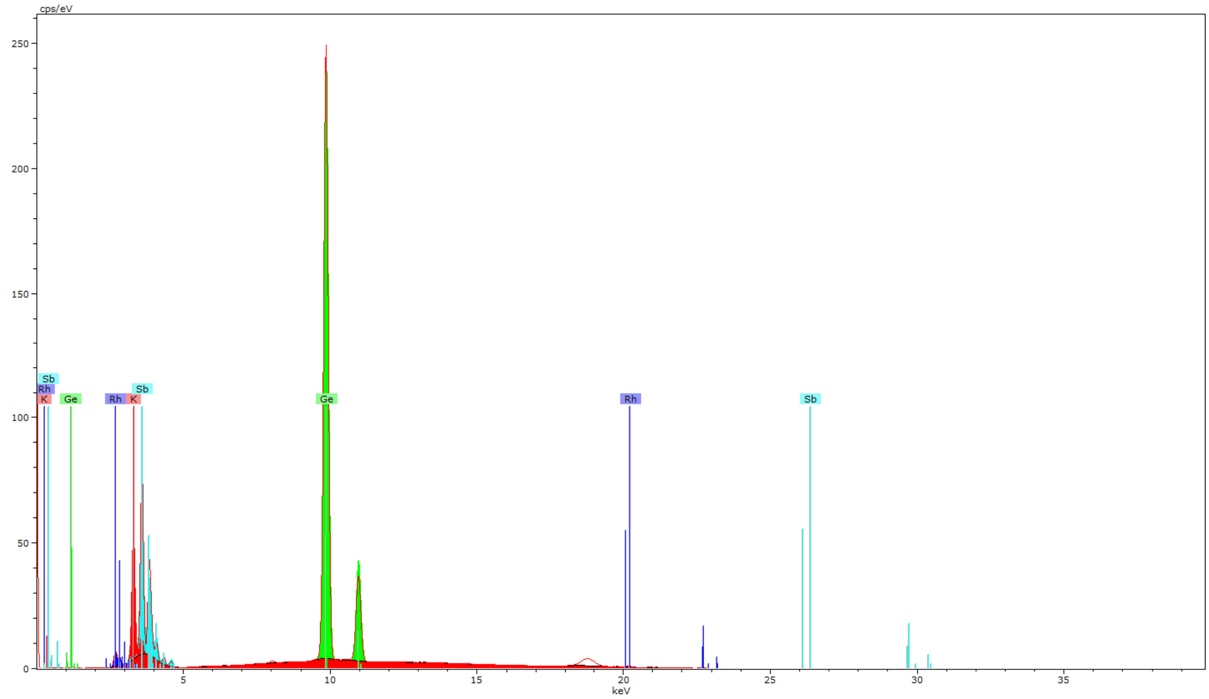

Figure S9. Micro X-ray fluorescence spectrum of **2** with the results of the deconvolution algorithm.

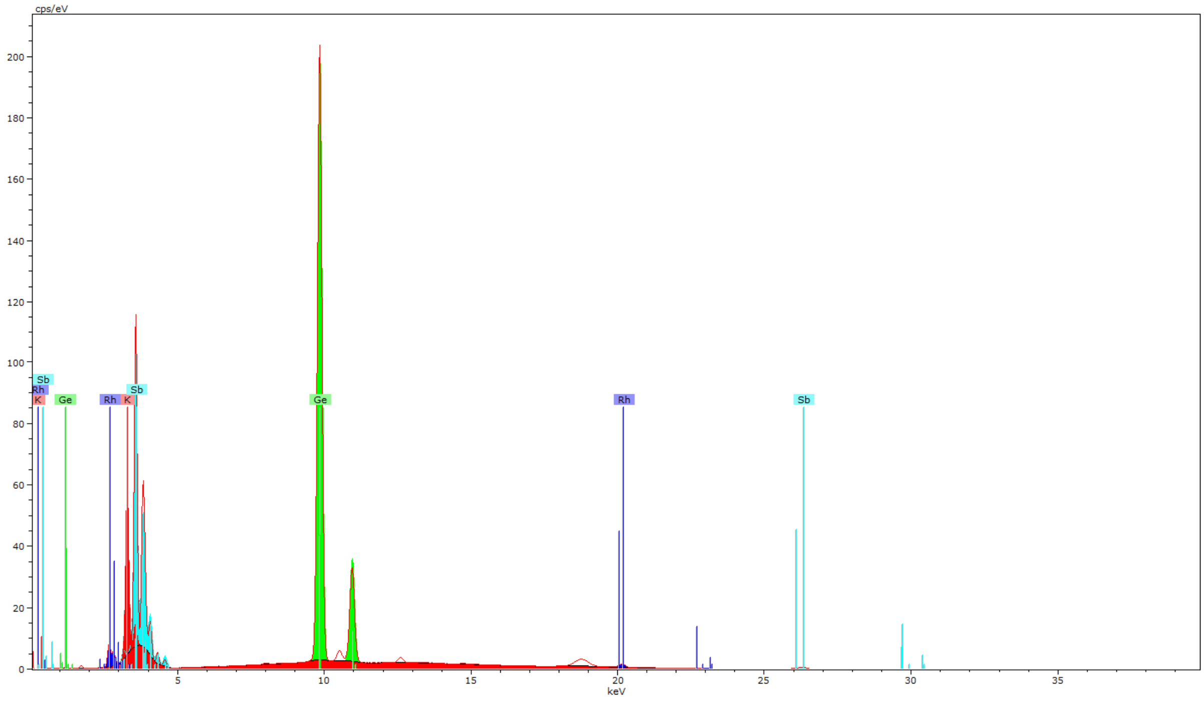

Figure S10. Micro X-ray fluorescence spectrum of compound **3** with the results of the deconvolution algorithm.

5. Electrospray ionization mass spectrometry (ESI-MS) investigations of the extraction process of

5.1 Methods

In order to explore the possible formation pathway, we prepared a series of reactive extraction solutions of $K_{12}Ge_{3.5}Sb_6$ (**1**) in en, like those from which compounds **2** and **3** were crystallized. The reaction solutions were allowed to stir for 5 minutes, 30 minutes, 3 hours, 1 day, 1 week and 2 weeks, before the measurements.

All mass spectra were recorded with a Thermo Fischer Scientific Finnigan LTQ-FT spectrometer in negative ion mode. The solutions were injected into the spectrometer with gastight 250 µL Hamilton syringes by syringe pump infusion. All capillaries within the system were washed with dry en/toluene mixture (1:1) 2 hours before and at least 10 min in between measurements to avoid decomposition reactions and consequent clogging.

The following ESI parameters were used: Spray Voltage: 3.6 kV, Capillary Temp: 290 °C, Capillary Voltage: −20 kV, Tube lens Voltage: −121.75 kV, Sheath Gas: 45, Sweep Gas: 0, Auxiliary Gas: 40. Assignable high-resolution mass peaks are shown in Figures S11–S21.

5.1.1 Mass spectra of the reaction solution yielding 2 and 3 (5 min)

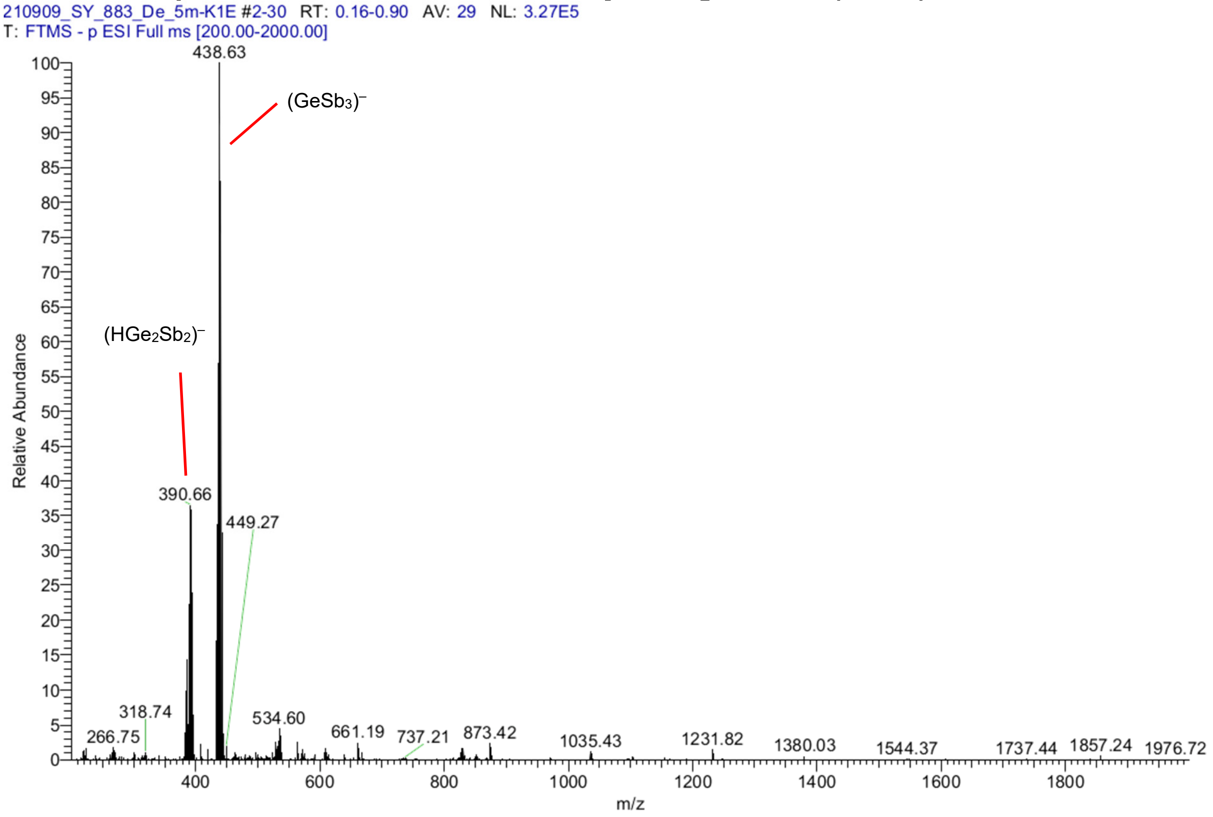

Figure S11. Overview of ESI(−) mass spectrum recorded immediately upon injection of a fresh reaction solution yielding **2** and **3** in en (5 min).

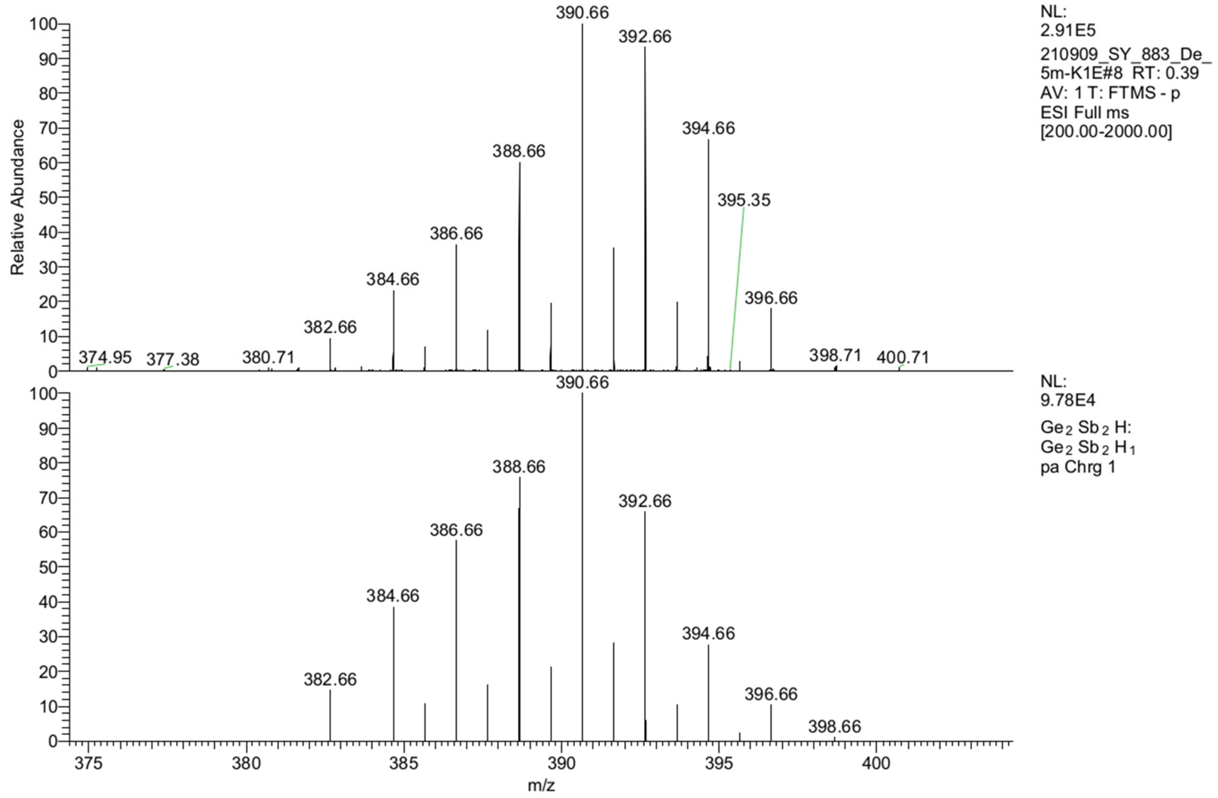

Figure S12. High-resolution ESI mass spectrum in negative ion mode recorded immediately upon injection of a fresh reaction solution yielding **2** and **3** in en (5 min), indicating the existence of $(HGe_2Sb_2)^-$. Topmost: measured, below: simulated.

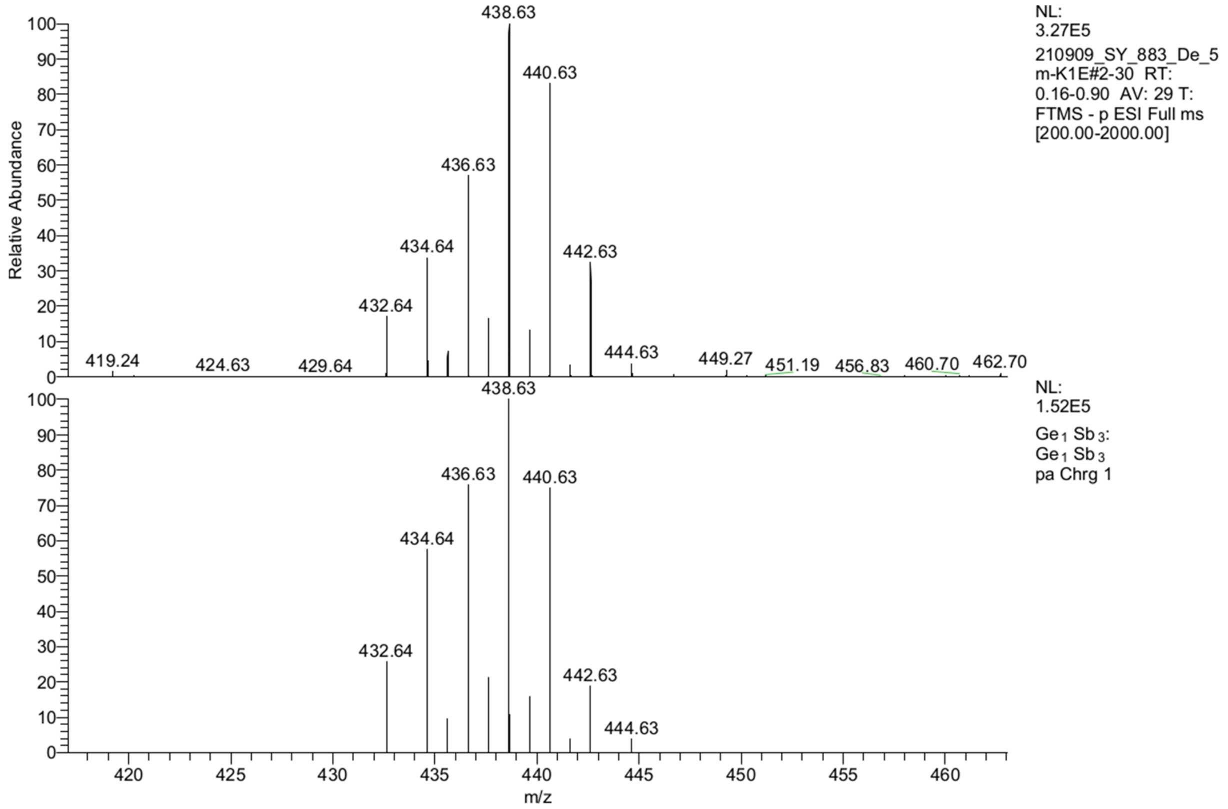

Figure S13. High-resolution ESI mass spectrum in negative ion mode recorded immediately upon injection of a fresh reaction solution yielding **2** and **3** in en (5 min), indicating the existence of $(GeSb_3)^-$. Topmost: measured, below: simulated.

5.1.2 Mass spectra of the reaction solution yielding 2 and 3 (30 min)

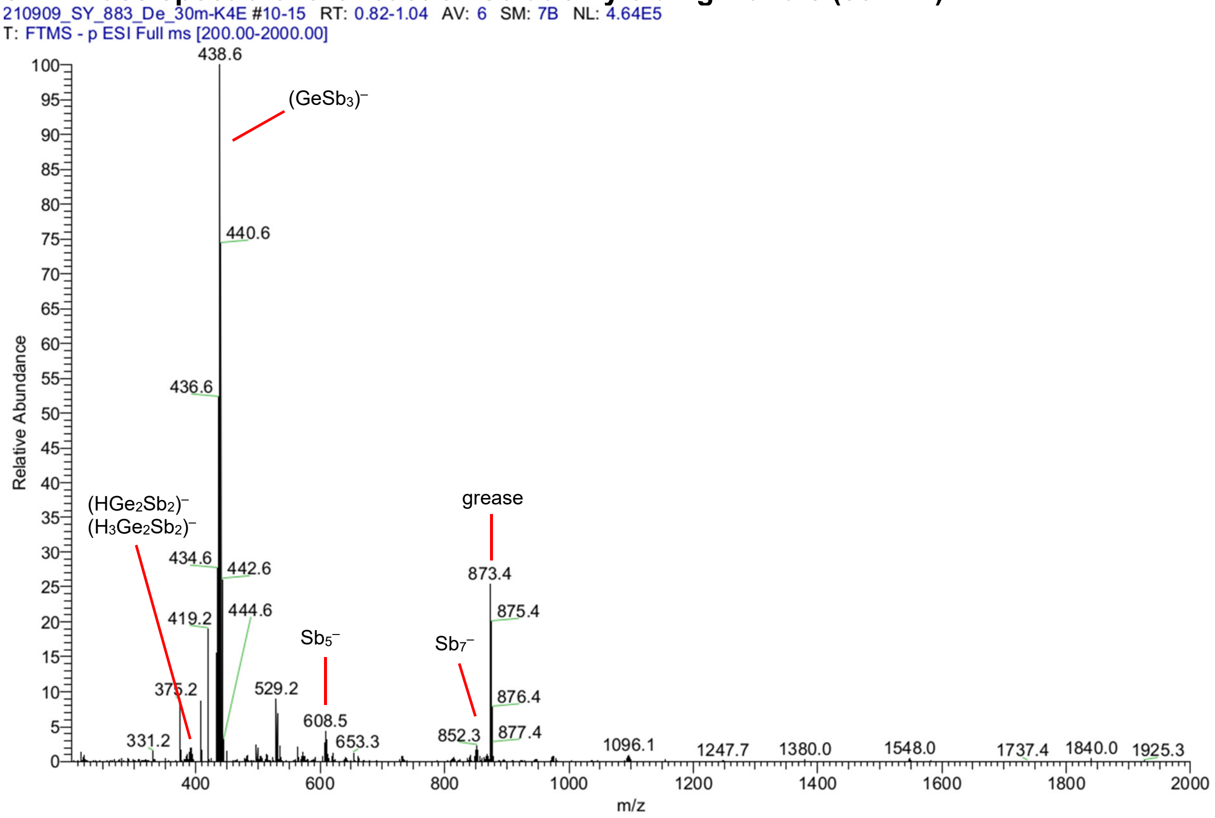

Figure S14. Overview of ESI(−) mass spectrum recorded immediately upon injection of a fresh reaction solution yielding **2** and **3** in en (30 min).

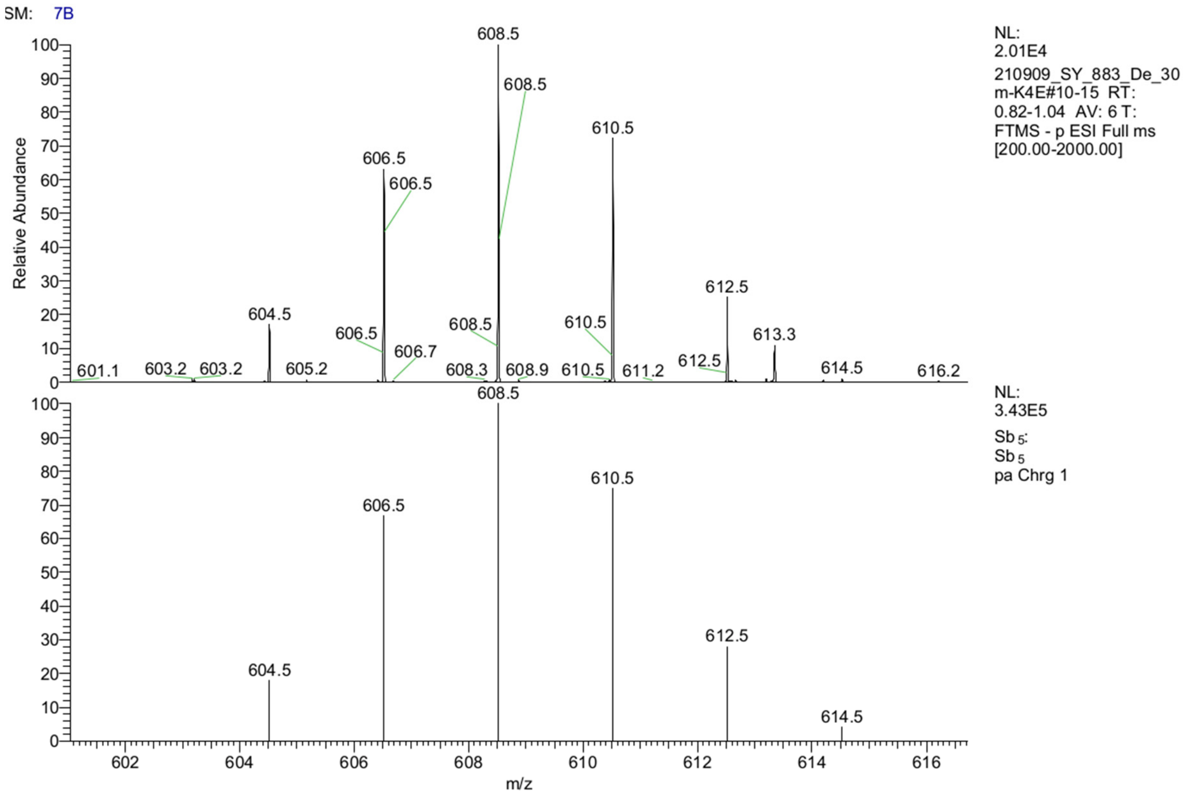

Figure S15. High-resolution ESI mass spectrum in negative ion mode recorded immediately upon injection of a fresh reaction solution yielding **2** and **3** in en (30 min), indicating the existence of Sb_5^-. Topmost: measured, below: simulated.

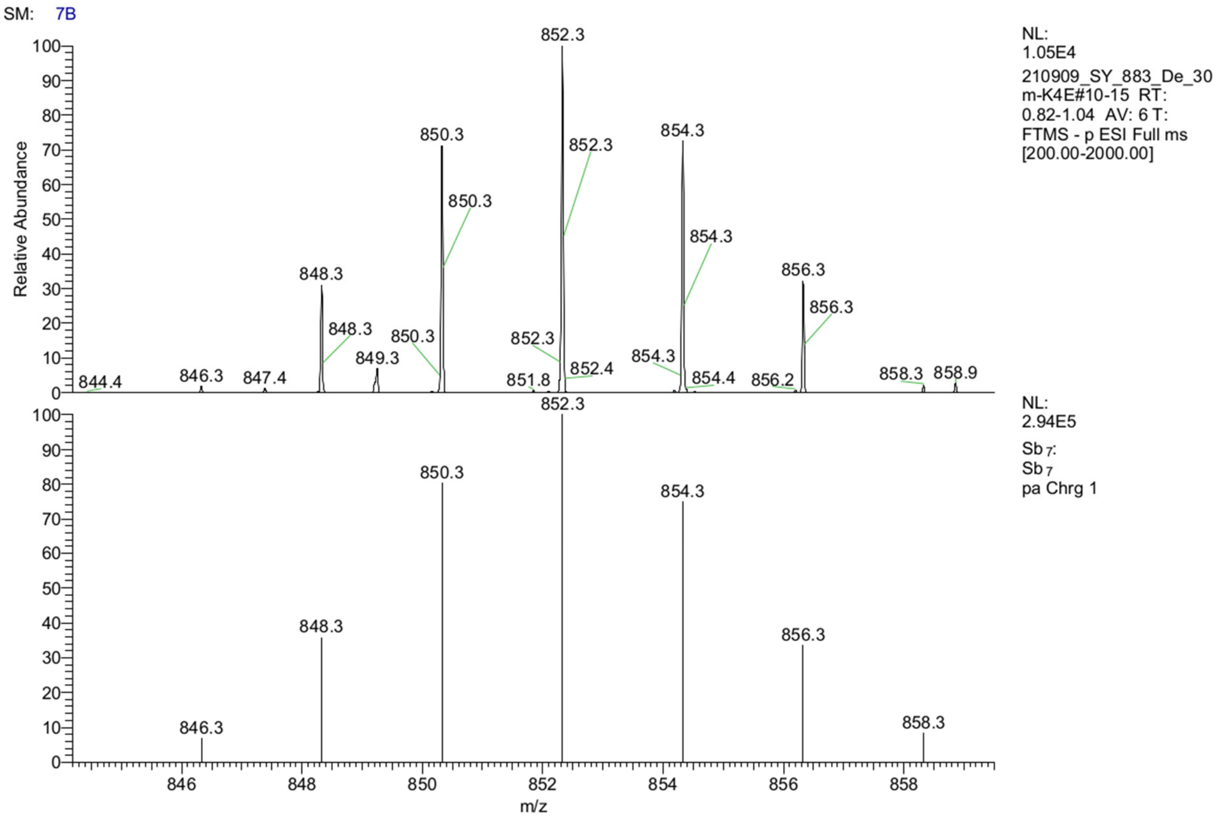

Figure S16. High-resolution ESI mass spectrum in negative ion mode recorded immediately upon injection of a fresh reaction solution yielding **2** and **3** in en (30 min), indicating the existence of Sb_7^-. Topmost: measured, below: simulated.

5.1.3 Mass spectra of the reaction solution yielding 2 and 3 (3 h)

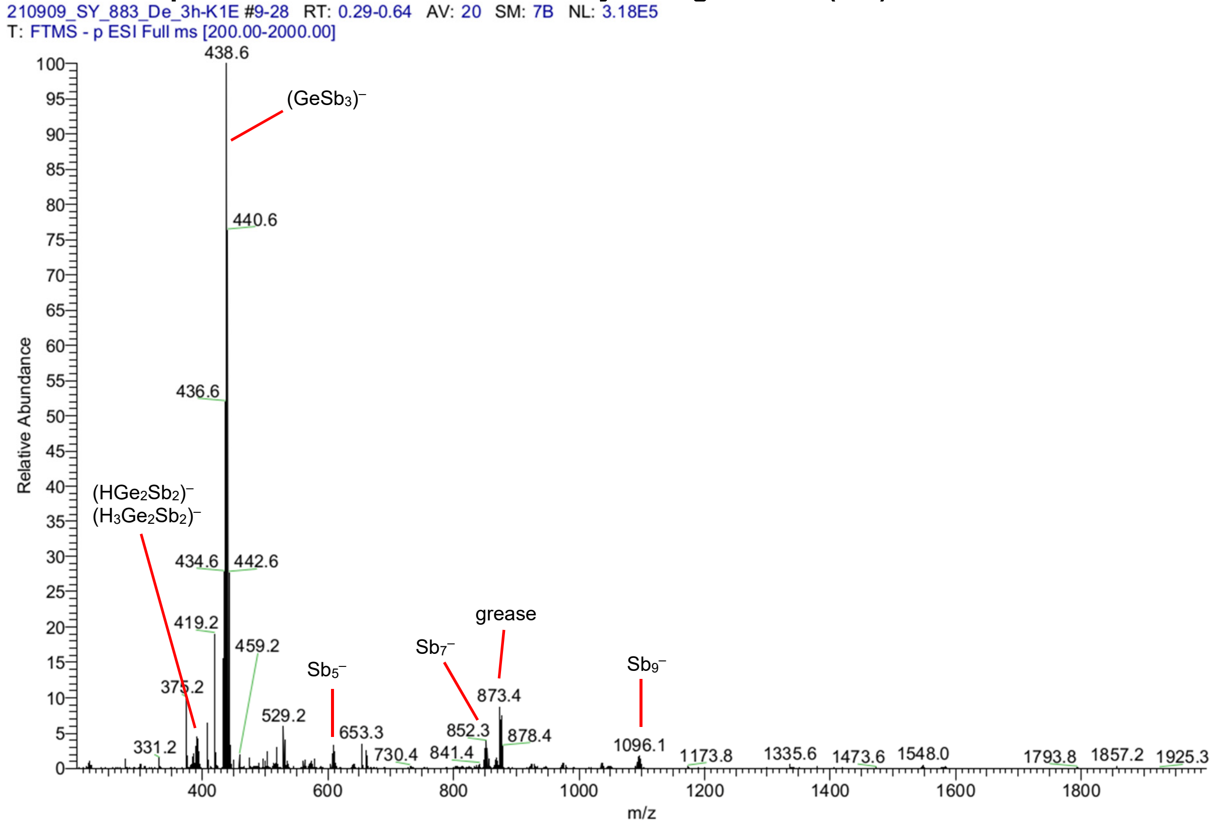

Figure S17. Overview of ESI(−) mass spectrum recorded immediately upon injection of a fresh reaction solution yielding **2** and **3** in en (3 h).

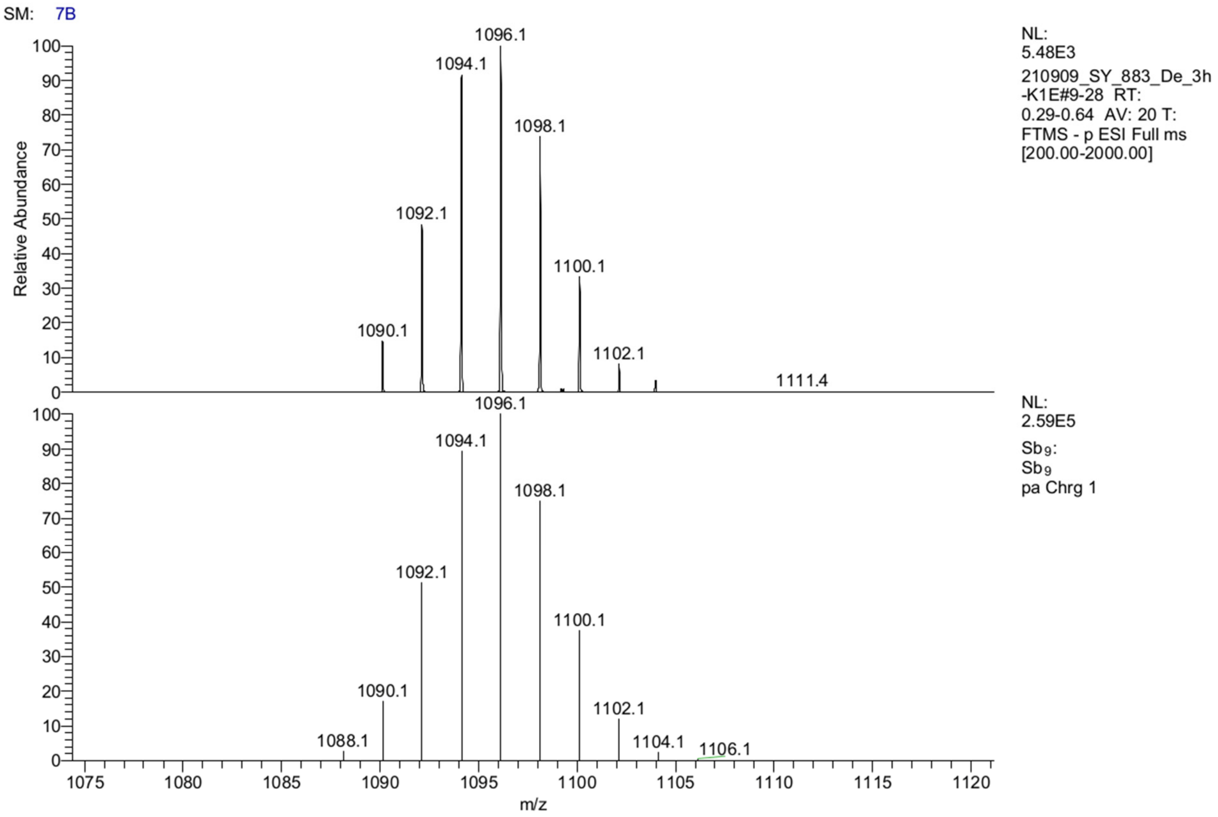

Figure S18. High-resolution ESI mass spectrum in negative ion mode recorded immediately upon injection of a fresh reaction solution yielding **2** and **3** in en (30 min), indicating the existence of Sb_9^-. Topmost: measured, below: simulated.

5.1.4 Mass spectra of the reaction solution yielding 2 and 3 (1 d)

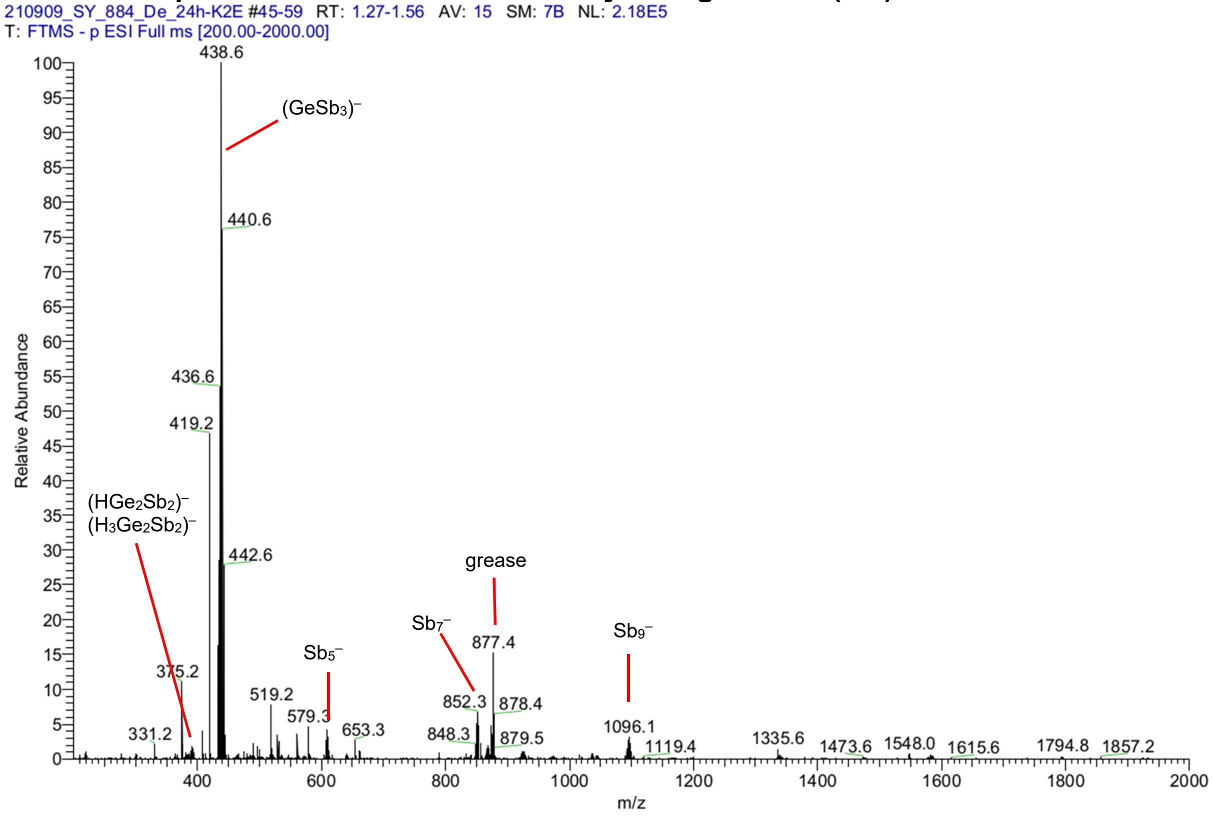

Figure S19. Overview of ESI(−) mass spectrum recorded immediately upon injection of a fresh reaction solution yielding **2** and **3** in en (1 d).

5.1.5 Mass spectra of the reaction solution yielding 2 and 3 (1w and 2w)

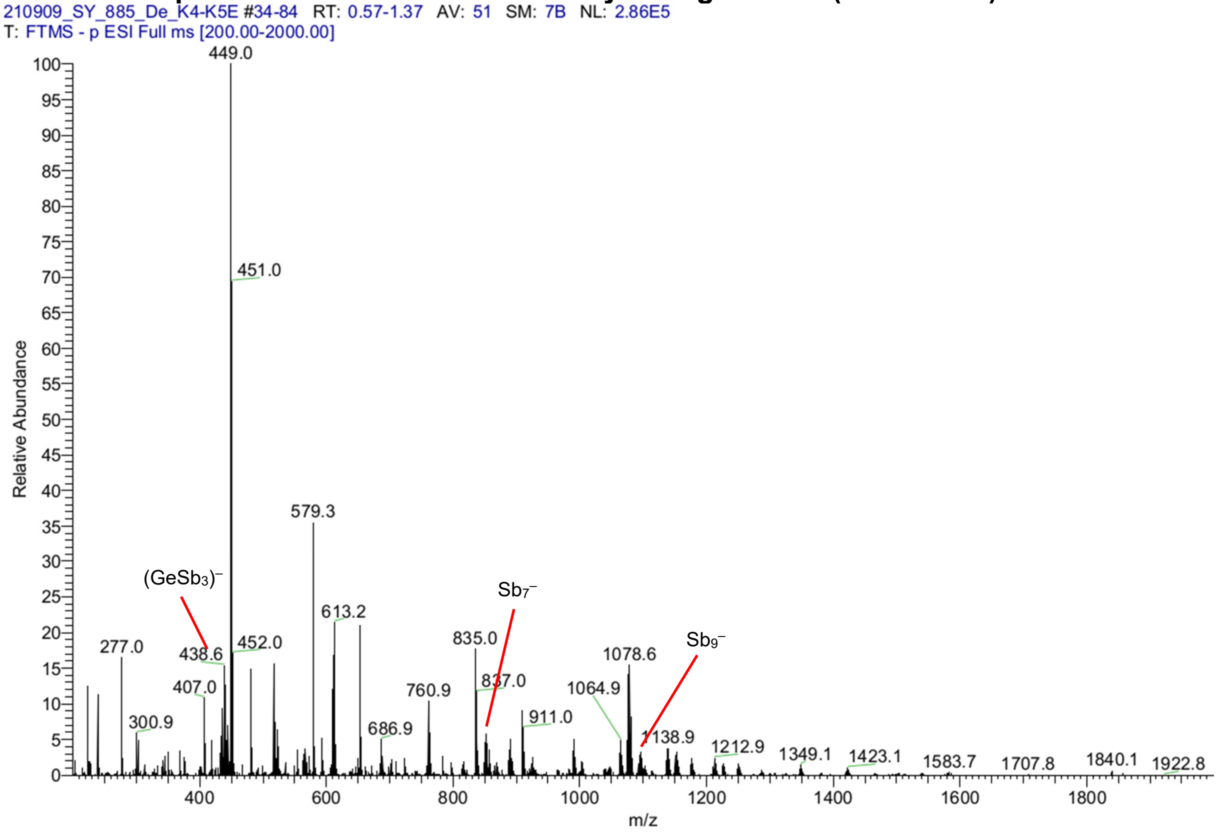

Figure S20. Overview of ESI(−) mass spectrum recorded immediately upon injection of a fresh reaction solution yielding **2** and **3** in en (1 w).

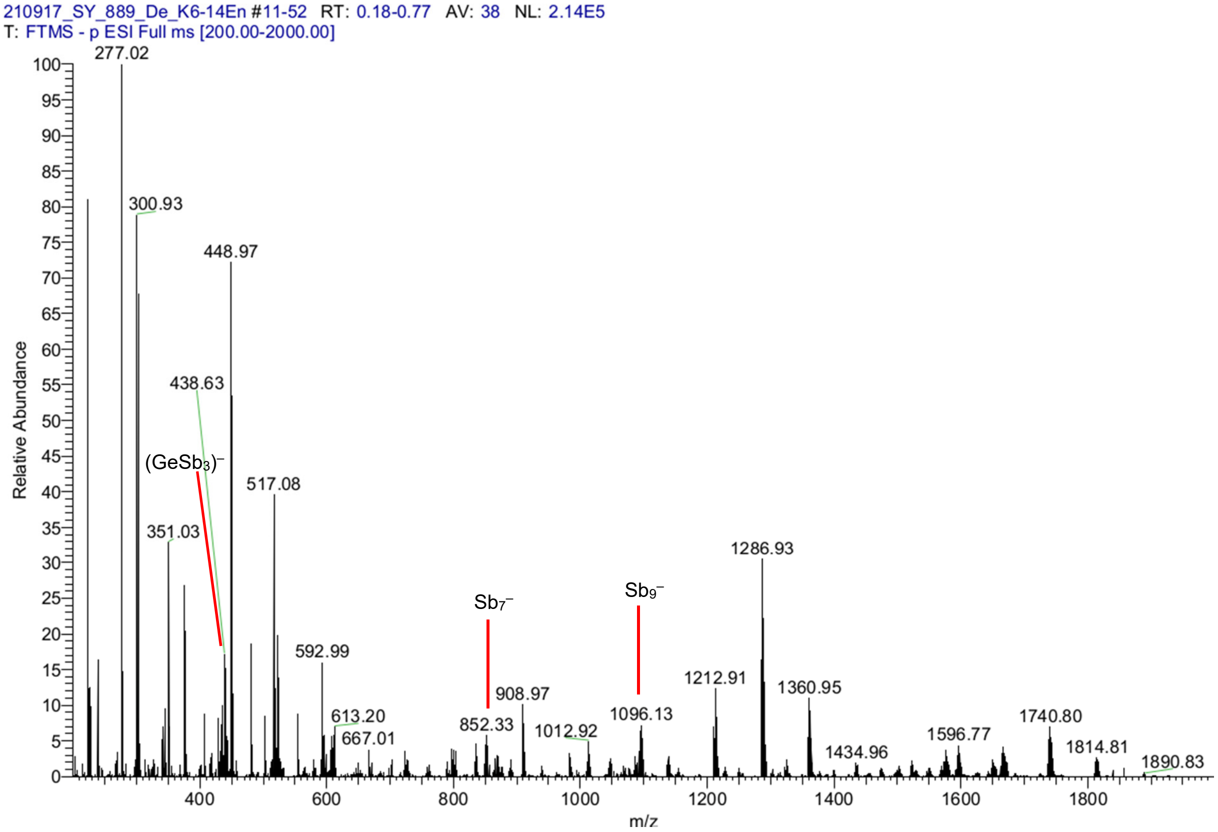

Figure S21. Overview of ESI(−) mass spectrum recorded immediately upon injection of a fresh reaction solution yielding **2** and **3** in en (2 w).

6. Quantum Chemical Investigations

6.1 Methods of the DFT Studies of the Solid State Compound 1

Structural optimization based on the crystallographic data was conducted using the *Vienna Ab initio Simulation Package* (VASP)[5] with a 1×1×2 supercell to model the unusual distribution of the sub-occupied Ge2 and Ge3 atoms along the *c*-axis. Subsequent bond analysis was conducted with the help of the Local Orbital Basis Suite Towards Electronic Structure Reconstruction (LOBSTER)[6] package by projecting the PAW-based[7] wavefunctions onto a local orbital basis. This allowed for the calculation of Löwdin charges[8] and bond orders as expressed in the crystal orbital bond index (COBI).[9]

6.2 Methods of the DFT Studies of Molecular Anions

Simultaneous optimization of the geometric and electronic structures of the anions in **1** – **4** were undertaken using the program system Turbomole V7.5.1.[10-11] Density functional theory (DFT)[12] methods were employed throughout these studies. We employed the TPSS functional[13] and basis sets of quality dhf-TZVP[14] with additional use of auxiliary basis sets[15] and effective core potentials[16] at the Sb atoms. Negative charges were compensated with the conductor-like screening model (COSMO).[17] Partial charges were calculated by means of Mulliken[18] and natural population analyses (NPA).[19] Localized molecular orbitals were obtained according to Boys' method.[20]

The anions in **1** – **4** were initially optimized without any symmetry restrictions and later re-optimized with higher symmetry point groups, where appropriate (**1**: D_{3h}, **2**: C_{2v}, **4**: C_{2h}). Calculation of force constants with the module NumForce verified all structures to be minima on the potential energy hypersurface.

6.3 Details of the Quantum Chemical Investigations of Compound 1

The chosen convergence criteria for the VASP structure optimization were 10^{-8} eV for electronic and $5 \cdot 10^{-3}$ eV/Å for ionic iteration steps whereas the kinetic energy cutoff was 500 eV. The VASP-recommended PAW pseudopotentials were chosen for these calculations using a **k**-point mesh of 0.02 to 0.04 Å^{-1} with Blöchl's tetrahedron integration method[21] and a smearing factor of 0.05 eV. In addition, the solid-state-optimized generalized gradient approximation (GGA)[22] and the Becke-Johnson dampened D3-Method to approximate the van-der-Waals interactions[23] were chosen.

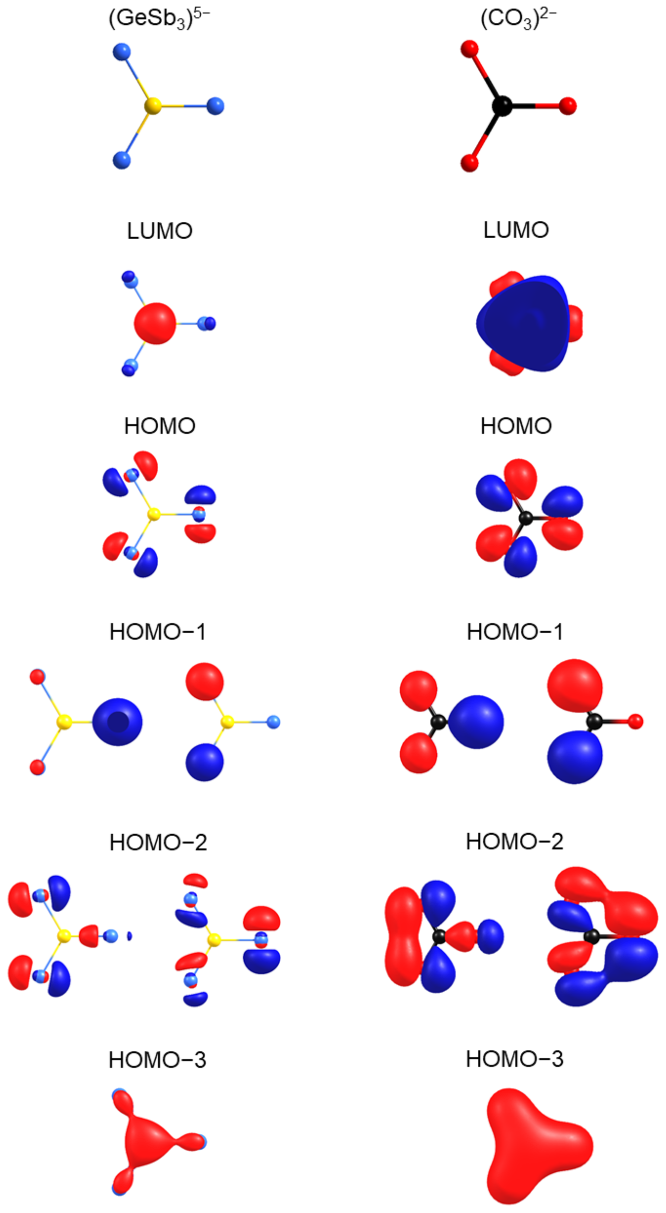

Figure S22. Comparison of the canonical MOs of the anion in **1** with $(CO_3)^{2-}$. Geometry optimizations were performed in point group D_{3h}. Contour values are drawn at ±0.05 a.u.

6.4 Details of the Quantum Chemical Investigations of Compound 2

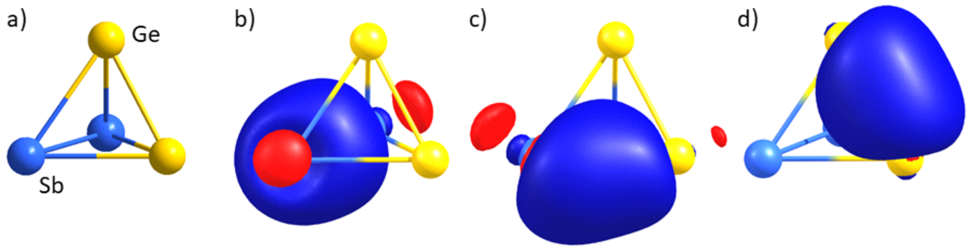

Figure S23. Computationally optimized structure and representative localized molecular orbitals (LMOs) of the anion in **2**. Contour values are drawn at ±0.05 a.u.

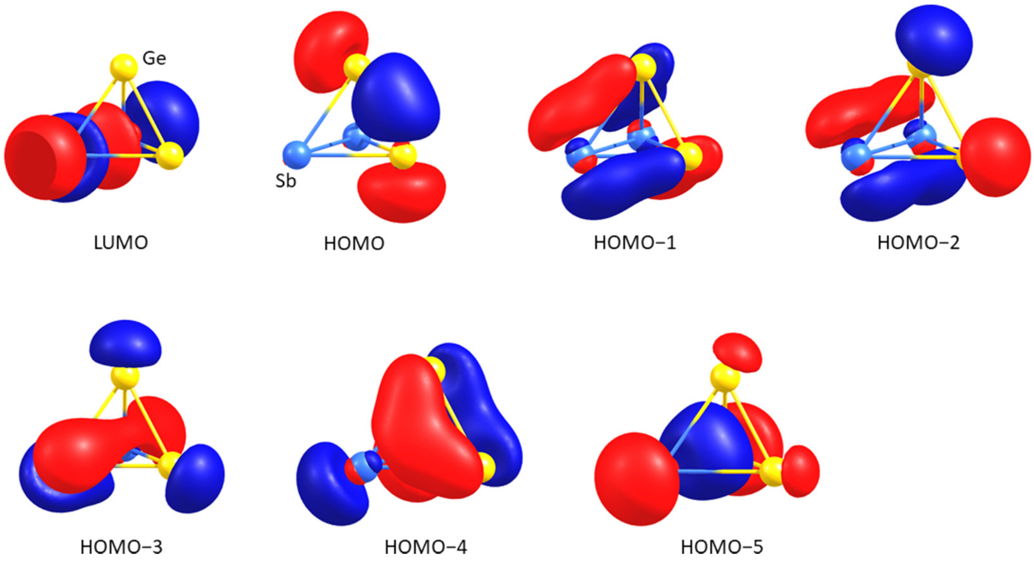

Figure S24. Canonical molecular orbitals of the anion in **2**. Contour values are drawn at ±0.05 a.u.

6.5 Details of the Quantum Chemical Investigations of the Anion in 3

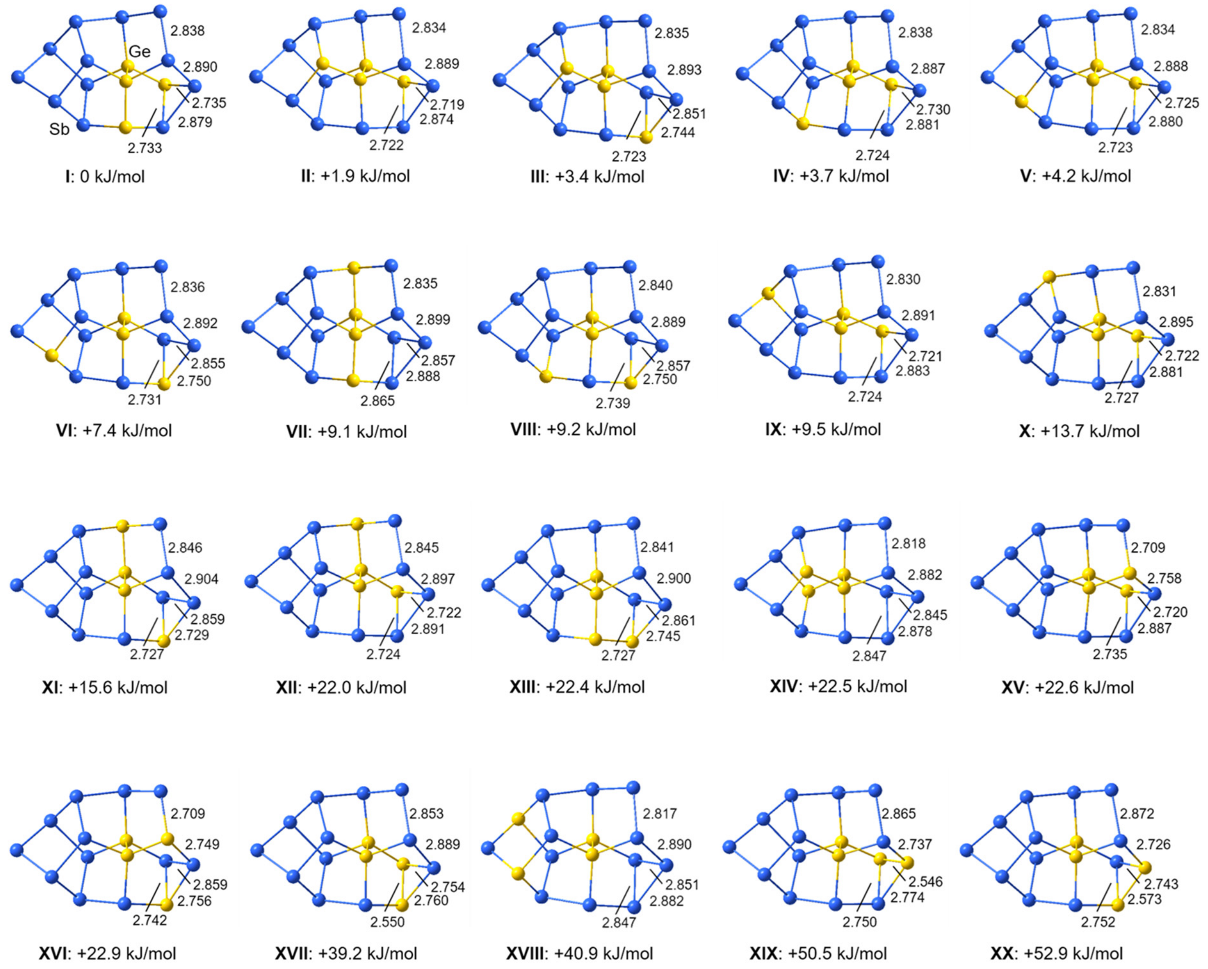

Figure S25. Overview of the 20 computationally obtained isomers of the anion in **3**.

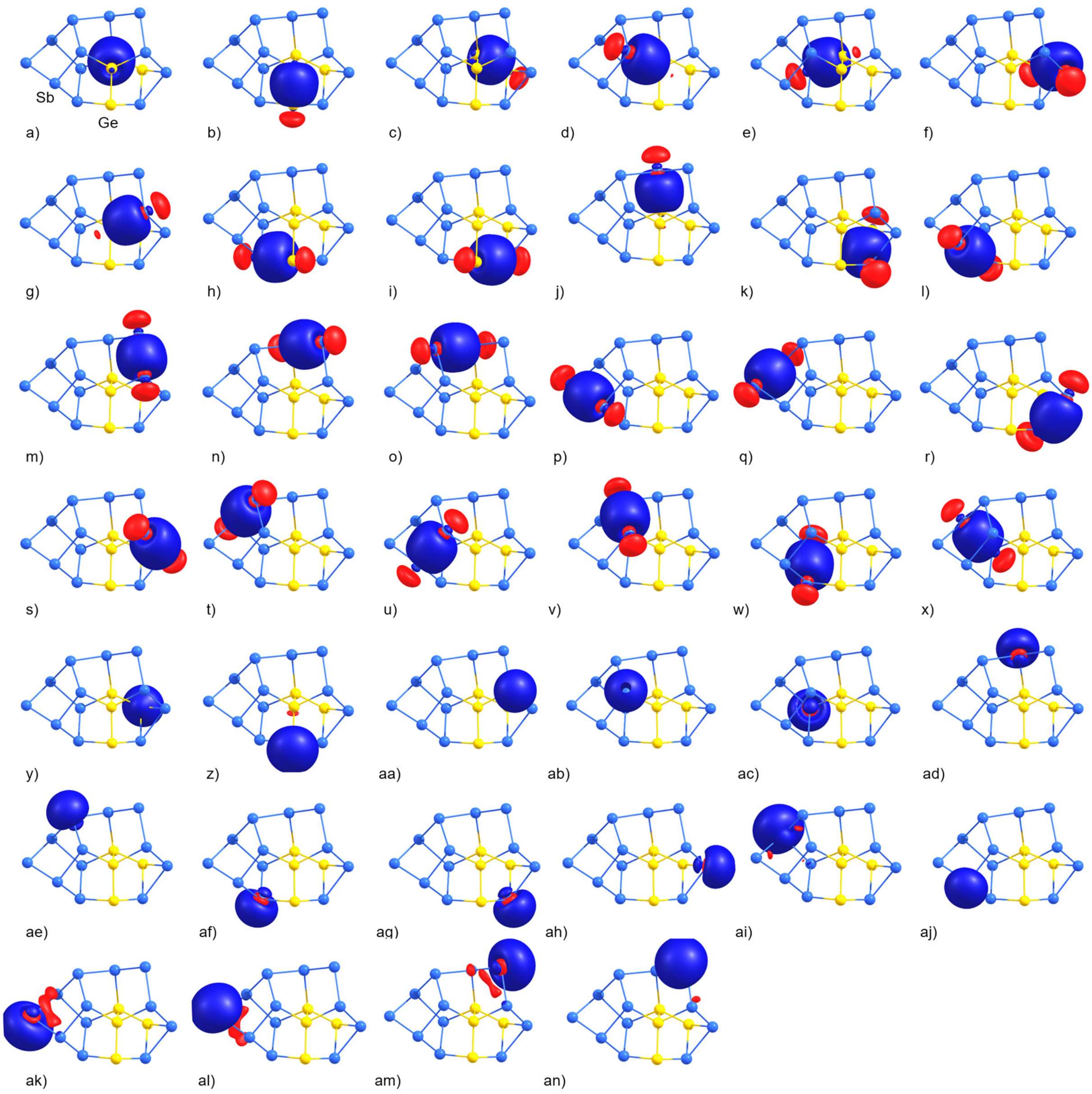

Figure S26. Localized molecular orbitals (LMOs) of the most stable isomer of the anion in **3** (isomer **I** in Figure S25), representing different two-center (a-v) and one-center (w-ak) interactions: Ge–Ge bonds (a–c), Ge–Sb bonds (d–k), Sb–Sb bonds (l–v), lone-pairs at a Ge atom (w, x) and at Sb atoms (y-ak). Contour values are drawn at ±0.05 a.u.

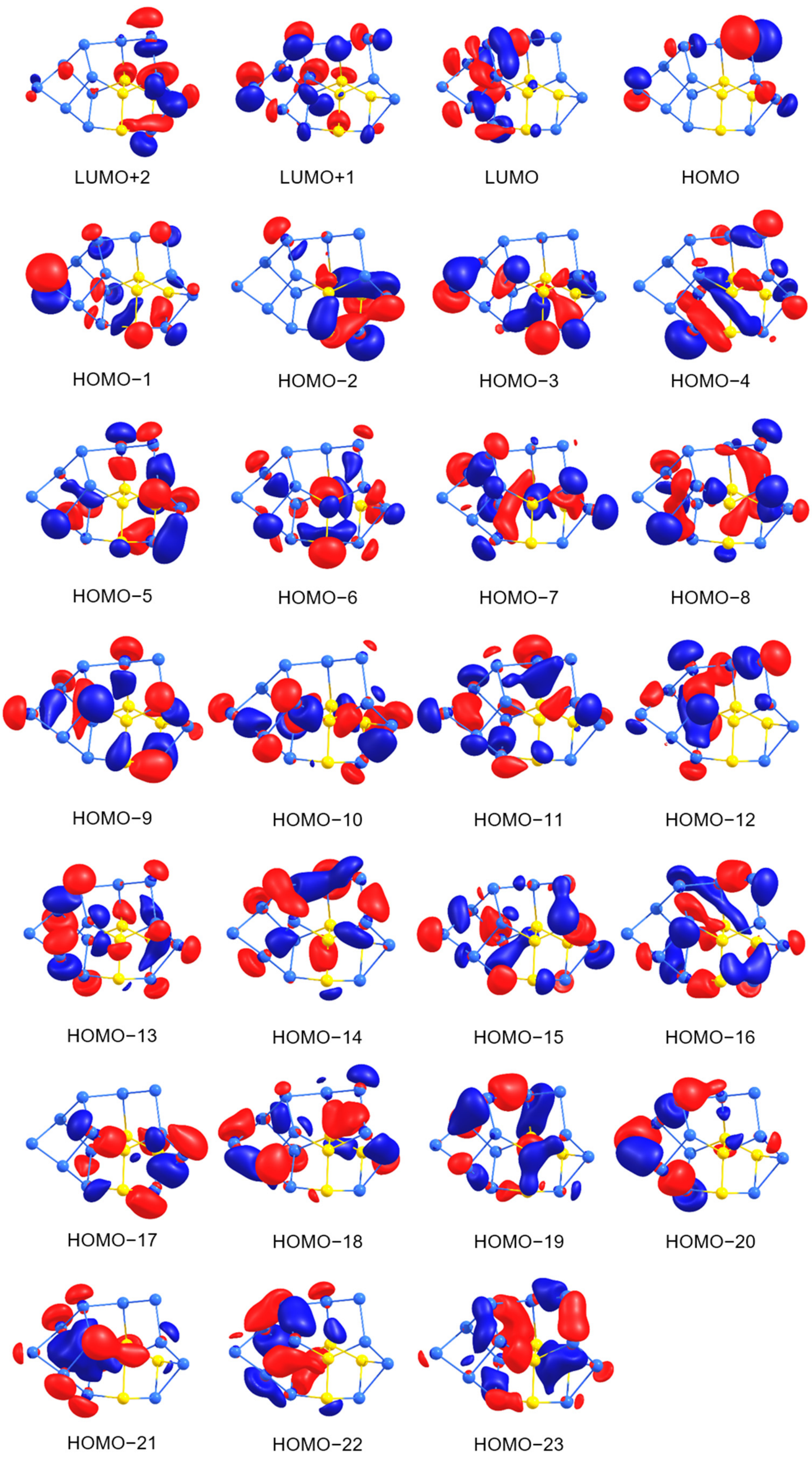

Figure S27. Canonical molecular orbitals of the most stable isomer of the anion in **3** (isomer **I** in Figure S25). Contour values are drawn at ±0.03 a.u.

6.6 Details of the Quantum Chemical Investigations of the Anion in 4

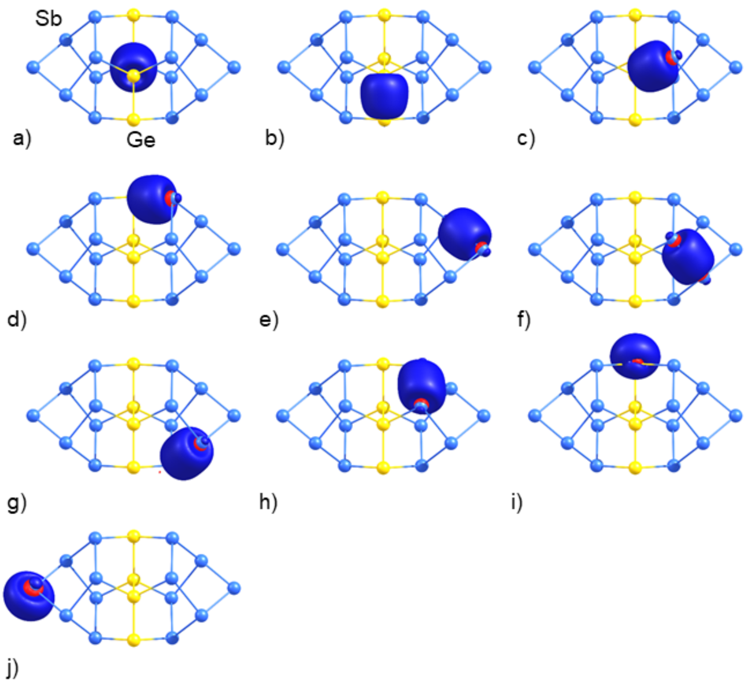

Figure S28. Representative localized molecular orbitals (LMOs) of the most stable isomer of the anion in **4**, representing different two-center (a-h) and one-center (i, j) interactions: Ge–Ge bonds (a–b), Ge–Sb bonds (c–d), Sb–Sb bonds (e–h), and of the lone-pair at Ge atoms (i) and at μ-bridging Sb atoms (j). Contour values are drawn at ±0.05 a.u.

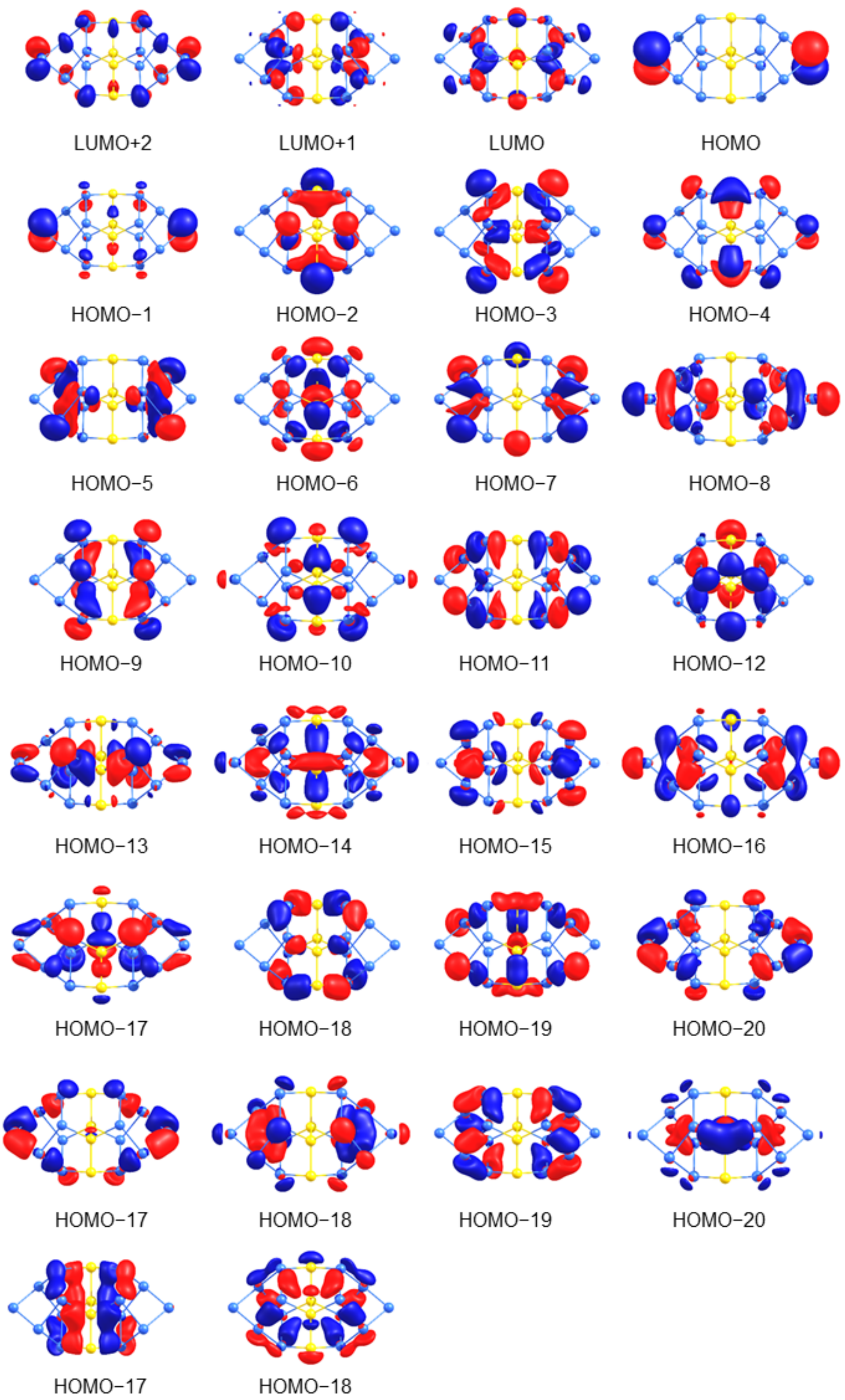

Figure S29. Canonical molecular orbitals of the most stable isomer of the anion in compound **4**. Contour values are drawn at ±0.03 a.u.

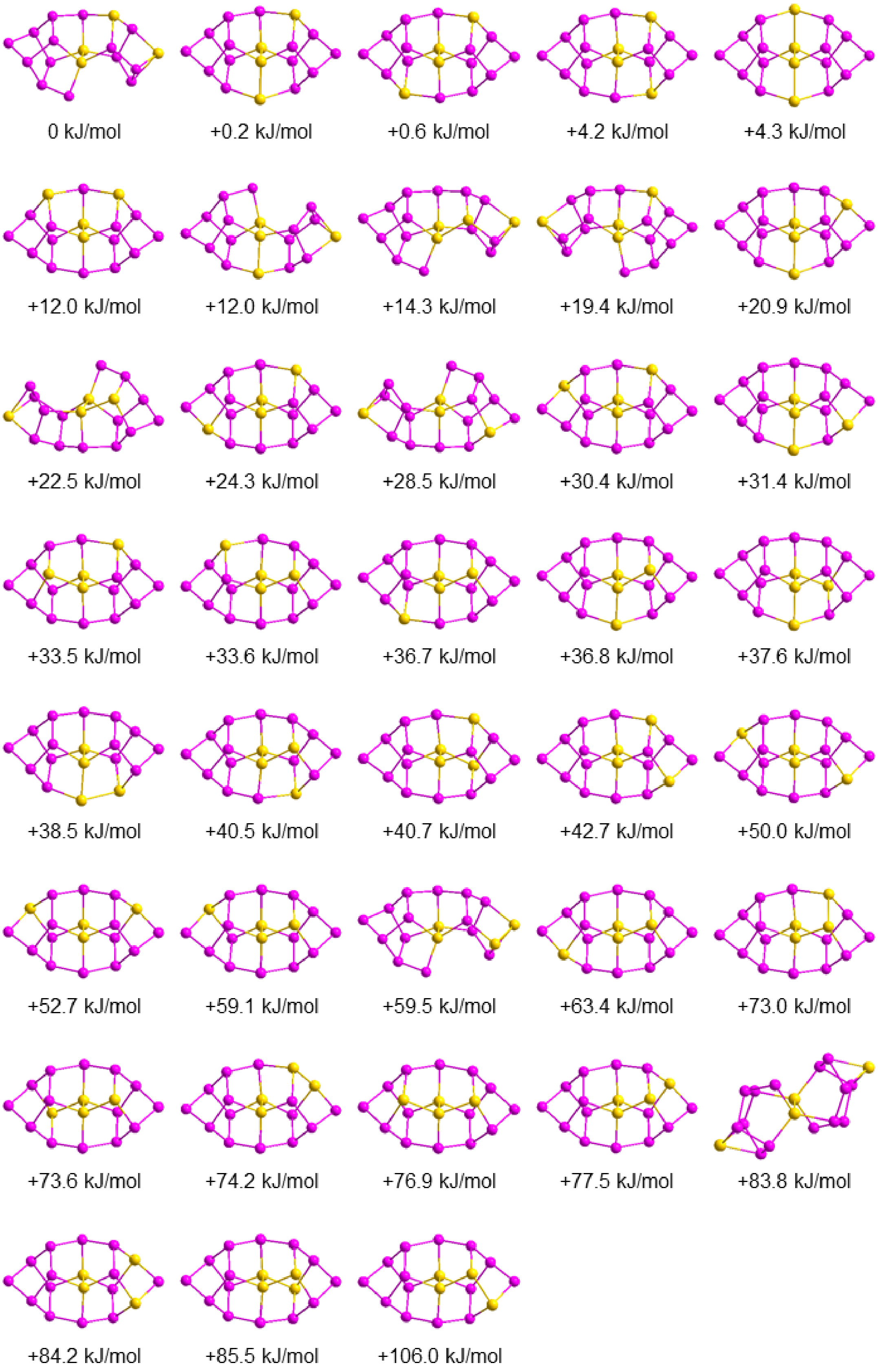

Figure S30. Calculated isomers of $(Ge_4P_{14})^{4-}$ and relative energies ΔE with respect to the global minimum structure.

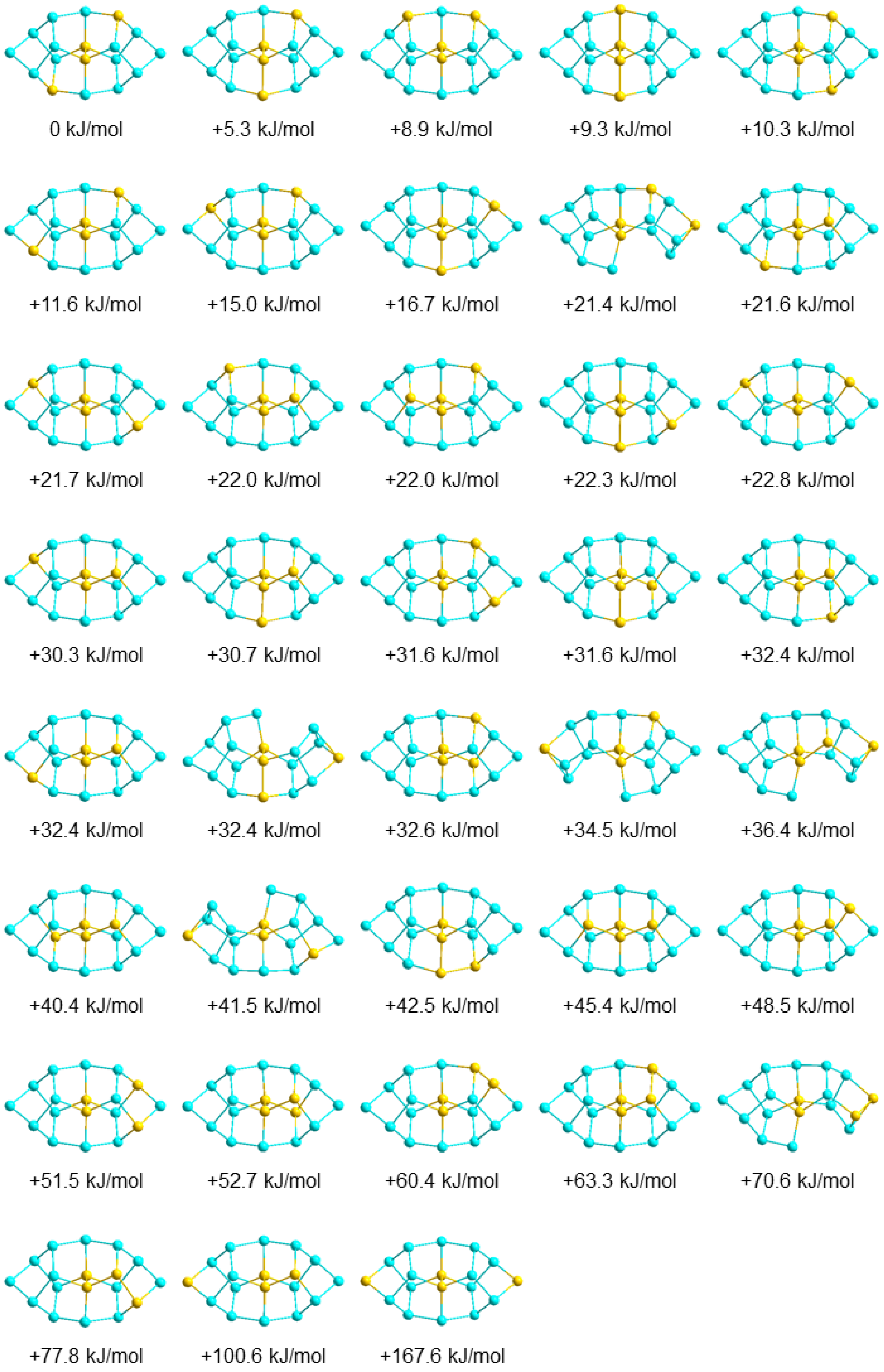

Figure S31. Calculated isomers of $(Ge_4As_{14})^{4-}$ and relative energies ΔE with respect to the global minimum structure.

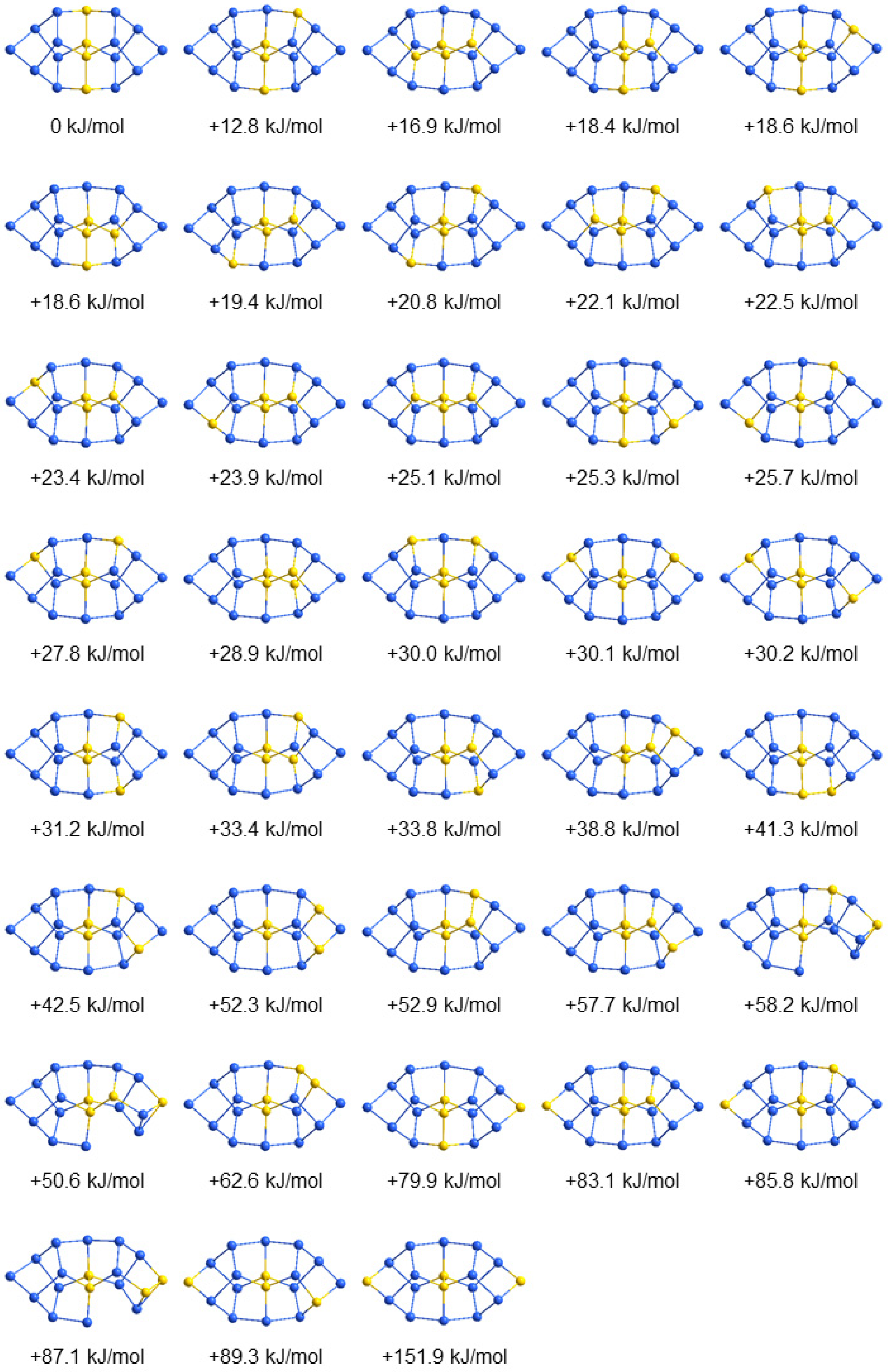

Figure S32. Calculated isomers of $(Ge_4Sb_{14})^{4-}$ (anion in **4**) and relative energies Δ*E* with respect to the global minimum structure.

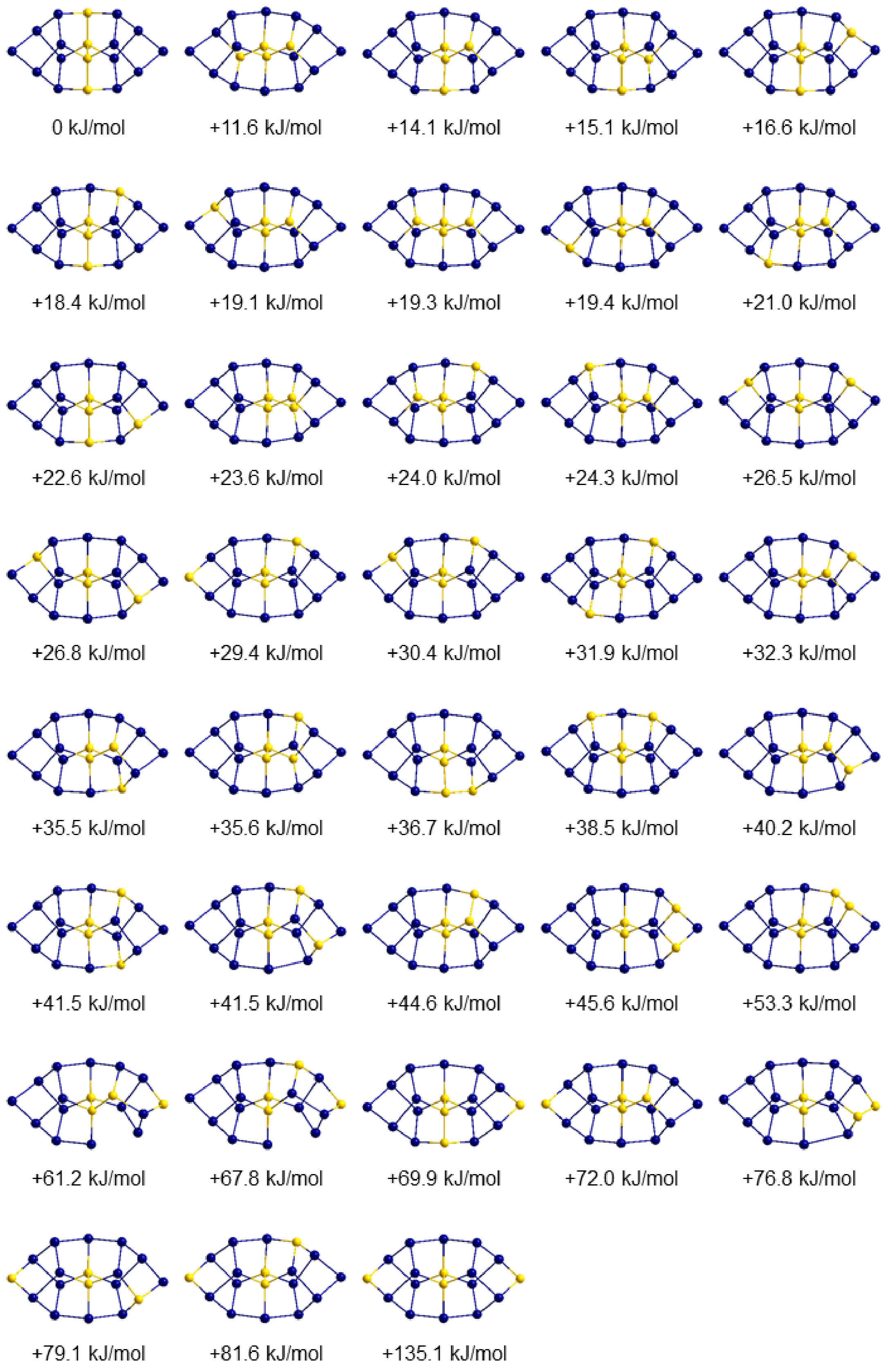

Figure S33. Calculated isomers of $(Ge_4Bi_{14})^{4-}$ and relative energies ΔE with respect to the global minimum structure.

7. References for the Supporting Information

[1] 4, 13,16,21,24-Hexaoxa-1,10-diazabicyclo[8.8.8]hexacosane.

[2] G. Sheldrick, *Acta Crystallogr. A* **2015**, *71*, 3–8.

[3] G. Sheldrick, *Acta Crystallogr. C* **2015**, *71*, 3–8.

[4] Diamond –Crystal and Molecular Structure Visualization, Crystal Impact – Dr. H. Putz & Dr. K. Brandenburg GbR, Kreuzherrenstr. 102, 53227 Bonn, Germany, http://www.crystalimpact.com/diamond.

[5] S. Parsons, H. D. Flack, T. Wagner, *Acta Crystallogr. B* **2013**, *69*, 249–259

[6] a) G. Kresse, J. Hafner, *Phys. Rev. B* **1993**, *47*, 558–561; b) G. Kresse, J. Furthmüller, *Comp. Mater. Sci.* **1996**, *6*, 15–50; c) G. Kresse, J. Furthmüller, *Phys. Rev. B* **1996**, *54*, 11169–11186; d) G. Kresse, D. Joubert, *Phys. Rev. B* **1999**, *59*, 1758–1775.

[7] a) S. Maintz, V. L. Deringer, A. L. Tchougréeff, R. Dronskowski, *J. Comput. Chem.* **2013**, *34*, 2557–2567; b) S. Maintz, V. L. Deringer, A. L. Tchougréeff, R. Dronskowski, *J. Comput. Chem.* **2016**, *37*, 1030–1035; c) R. Nelson, C. Ertural, J. George, V. L. Deringer, G. Hautier, R. Dronskowski, *J. Comput. Chem.* **2020**, *41*, 1931–1940.

[8] P. E. Blöchl, *Phys. Rev. B* **1994**, *50*, 17953–17979.

[9] C. Ertural, S. Steinberg, R. Dronskowski, *RSC Adv.* **2019**, *9*, 29821–29830.

[10] P. C. Müller, C. Ertural, J. Hempelmann, R. Dronskowski, *J. Chem. Phys. C* **2021**, *125*, 7959–7970.

[11] TURBOMOLE V7.5.1 (2020), a development of University of Karlsruhe and Forschungszentrum Karlsruhe GmbH, 1989–2007, TURBOMOLE GmbH, since 2007; available from http://www.turbomole.com.

[12] S. G. Balasubramani, G. P. Chen, S. Coriani, M. Diedenhofen, M. S. Frank, Y. J. Franzke, F. Furche, R. Grotjahn, M. E. Harding, C. Hättig, A. Hellweg, B. Helmich-Paris, C. Holzer, U. Huniar, M. Kaupp, A. Marefat Khah, S. Karbalaei Khani, T. Müller, F. Mack, B. D. Nguyen, S. M. Parker, E. Perlt, D. Rappoport, K. Reiter, S. Roy, M. Rückert, G. Schmitz, M. Sierka, E. Tapavicza, D. P. Tew, C. van Wüllen, V. K. Voora, F. Weigend, A. Wodyński, J. M. Yu, *J. Chem. Phys.* **2020**, *152*, 184107.

[13] K. Eichkorn, F. Weigend, O. Treutler, R. Ahlrichs, *Theor. Chem. Acc.* **1997**, *97*, 119–124.

[14] J. Tao, J. P. Perdew, V. N. Staroverov, G. E. Scuseria, *Phys. Rev. Lett.* **2003**, *91*, 146401.

[15] a) F. Weigend, C. Schrodt, *Chem. Eur. J.* **2005**, *11*, 3559–3564; b) F. Weigend, A. Baldes, *J. Chem. Phys.* **2010**, *133*, 174102.

[16] F. Weigend, *Phys. Chem. Chem. Phys.* **2006**, *8*, 1057–1065.

[17] a) B. Metz, H. Stoll, M. Dolg, *J. Chem. Phys.* **2000**, *113*, 2563–2569; b) D. Figgen, G. Rauhut, M. Dolg, H. Stoll, *Chem. Phys.* **2005**, *311*, 227–244.

[18] A. Klamt, G. Schüürmann, *J. Chem. Soc., Perkin Trans. 2* **1993**, 799–805.

[19] R. S. Mulliken, *J. Chem. Phys.* **1955**, *23*, 2338–2342.

[20] A. E. Reed, R. B. Weinstock, F. Weinhold, *J. Chem. Phys.* **1985**, *83*, 735–746.

[21] S. F. Boys, in *Quantum Theory of Atoms, Molecules and the Solid State* (Ed.: P.-O. Löwdin), Academic Press, New York **1966**, pp. 253–262.

[22] P. E. Blöchl, O. Jepsen, O. K. Andersen, *Phys. Rev. B* **1994**, *49*, 16223–16233.

[23] G. I. Csonka, J. P. Perdew, A. Ruzsinszky, P. H. T. Philipsen, S. Lebègue, J. Paier, O. A. Vydrov, J. G. Ángyán, *Phys. Rev. B* **2009**, *79*, 155107.

[24] a) S. Grimme, J. Antony, S. Ehrlich, H. Krieg, *J. Chem. Phys.* **2010**, *132*, 154104; b) S. Grimme, S. Ehrlich, L. Goerigk, *J. Comput. Chem.* **2011**, *32*, 1456–1465.
