# Supplementary material for: Between Elemental Match and Mismatch: From K12Ge3.5Sb6 to Salts of (Ge2Sb2)2−, (Ge4Sb12)4−, and (Ge4Sb14)4−
Source: Angew Chem Int Ed Engl. 2022 Jul 28;61(41):e202207232. doi: 10.1002/anie.202207232 (PMC9796001; doi:10.1002/anie.202207232)

checkCIF/PLATON report

Structure factors have been supplied for datablock(s) K12Ge4Sb6

THIS REPORT IS FOR GUIDANCE ONLY. IF USED AS PART OF A REVIEW PROCEDURE FOR PUBLICATION, IT SHOULD NOT REPLACE THE EXPERTISE OF AN EXPERIENCED CRYSTALLOGRAPHIC REFEREE.

No syntax errors found.   CIF dictionary   Interpreting this report

Datablock: K12Ge4Sb6

```
Bond precision:   Sb-Ge = 0.0009 A         Wavelength=0.71073

Cell:             a=13.3366(19)     b=13.3366(19)     c=5.3594(11)
                  alpha=90          beta=90           gamma=120
Temperature:      100 K

                 Calculated                   Reported
Volume           825.5(3)                     825.5(3)
Space group      P 63/m                       P 63/m
Hall group       -P 6c                        -P 6c
Moiety formula   Ge1.50, 2(Ge Sb3), 12(K)     ?
Sum formula      Ge3.50 K12 Sb6               Ge0.29 K Sb0.50
Mr               1453.89                      121.15
Dx,g cm-3        2.925                        2.924
Z                1                            12
Mu (mm-1)        9.467                        9.467
F000             646.0                        646.0
F000'            645.60
h,k,lmax         16,16,6                      16,16,6
Nref             648                          650
Tmin,Tmax        0.572,0.685
Tmin'            0.464

Correction method= Not given

Data completeness= 1.003            Theta(max)= 26.703

                                                      wR2(reflections)=
R(reflections)= 0.0381( 514)                          0.0952( 650)

S = 1.073                   Npar= 25
```

The following ALERTS were generated. Each ALERT has the format
test-name_ALERT_alert-type_alert-level.
Click on the hyperlinks for more details of the test.

Alert level B

```
PLAT213_ALERT_2_B Atom Ge3             has ADP max/min Ratio .....        4.5 prolat
```

Author Response: This is due to a situation of statistical disorder and partial occupation involving Ge3.

Alert level C

```
CRYSC01_ALERT_1_C No recognised colour has been given for crystal colour.
RINTA01_ALERT_3_C  The value of Rint is greater than 0.12
            Rint given   0.134
PLAT057_ALERT_3_C Correction for Absorption Required   RT(exp) ...       1.20 Do !
PLAT077_ALERT_4_C Unitcell Contains Non-integer Number of Atoms ..     Please Check
PLAT723_ALERT_1_C Torsion Calc       90.00, Rep        0.00 Dev...      90.00 Sigma
              GE2 -GE2 -GE3 -GE3   4_554  1_555  1_555  7_556      #    1 Check
PLAT723_ALERT_1_C Torsion Calc   -35.78(5), Rep        0.00 Dev...      35.78 Sigma
              K2  -GE2 -GE3 -GE3   1_555  1_555  1_555  7_556      #    2 Check
PLAT723_ALERT_1_C Torsion Calc    24.22(7), Rep        0.00 Dev...      24.22 Sigma
              K2  -GE2 -GE3 -GE3   9_556  1_555  1_555  7_556      #    3 Check
PLAT723_ALERT_1_C Torsion Calc  -155.78(7), Rep        0.00 Dev...     155.78 Sigma
              K2  -GE2 -GE3 -GE3   3_555  1_555  1_555  7_556      #    4 Check
PLAT723_ALERT_1_C Torsion Calc   -95.78(6), Rep        0.00 Dev...      95.78 Sigma
              K2  -GE2 -GE3 -GE3   8_556  1_555  1_555  7_556      #    5 Check
PLAT723_ALERT_1_C Torsion Calc    84.22(6), Rep        0.00 Dev...      84.22 Sigma
              K2  -GE2 -GE3 -GE3   2_555  1_555  1_555  7_556      #    6 Check
PLAT723_ALERT_1_C Torsion Calc   144.22(5), Rep        0.00 Dev...     144.22 Sigma
              K2  -GE2 -GE3 -GE3   7_556  1_555  1_555  7_556      #    7 Check
PLAT723_ALERT_1_C Torsion Calc       90.00, Rep        0.00 Dev...      90.00 Sigma
              GE2 -GE2 -GE3 -K2    4_554  1_555  1_555  1_555      #    8 Check
PLAT723_ALERT_1_C Torsion Calc       90.00, Rep        0.00 Dev...      90.00 Sigma
              GE2 -GE2 -GE3 -K2    4_554  1_555  1_555  3_555      #   14 Check
PLAT723_ALERT_1_C Torsion Calc       90.00, Rep        0.00 Dev...      90.00 Sigma
              GE2 -GE2 -GE3 -K2    4_554  1_555  1_555  2_555      #   20 Check
```

Alert level G

```
PLAT004_ALERT_5_G Polymeric Structure Found with Maximum Dimension          1 Info
PLAT020_ALERT_3_G The Value of Rint is Greater Than 0.12 .........      0.134 Report
PLAT045_ALERT_1_G Calculated and Reported Z Differ by a Factor ...      0.083 Check
PLAT300_ALERT_4_G Atom Site Occupancy of Ge2        Constrained at        0.5 Check
PLAT300_ALERT_4_G Atom Site Occupancy of Ge3        Constrained at       0.25 Check
PLAT301_ALERT_3_G Main Residue  Disorder .............(Resd  1  )       100% Note
PLAT767_ALERT_4_G INS Embedded LIST 6 Instruction Should be LIST 4     Please Check
PLAT883_ALERT_1_G No Info/Value for _atom_sites_solution_primary .     Please Do !
```

0 **ALERT level A** = Most likely a serious problem - resolve or explain
1 **ALERT level B** = A potentially serious problem, consider carefully

```
14 ALERT level C = Check. Ensure it is not caused by an omission or oversight
 8 ALERT level G = General information/check it is not something unexpected

13 ALERT type 1 CIF construction/syntax error, inconsistent or missing data
 1 ALERT type 2 Indicator that the structure model may be wrong or deficient
 4 ALERT type 3 Indicator that the structure quality may be low
 4 ALERT type 4 Improvement, methodology, query or suggestion
 1 ALERT type 5 Informative message, check
```

It is advisable to attempt to resolve as many as possible of the alerts in all categories. Often the minor alerts point to easily fixed oversights, errors and omissions in your CIF or refinement strategy, so attention to these fine details can be worthwhile. In order to resolve some of the more serious problems it may be necessary to carry out additional measurements or structure refinements. However, the purpose of your study may justify the reported deviations and the more serious of these should normally be commented upon in the discussion or experimental section of a paper or in the "special_details" fields of the CIF. checkCIF was carefully designed to identify outliers and unusual parameters, but every test has its limitations and alerts that are not important in a particular case may appear. Conversely, the absence of alerts does not guarantee there are no aspects of the results needing attention. It is up to the individual to critically assess their own results and, if necessary, seek expert advice.

Publication of your CIF in IUCr journals

A basic structural check has been run on your CIF. These basic checks will be run on all CIFs submitted for publication in IUCr journals (*Acta Crystallographica*, *Journal of Applied Crystallography*, *Journal of Synchrotron Radiation*); however, if you intend to submit to *Acta Crystallographica Section C* or *E* or *IUCrData*, you should make sure that full publication checks are run on the final version of your CIF prior to submission.

Publication of your CIF in other journals

Please refer to the *Notes for Authors* of the relevant journal for any special instructions relating to CIF submission.

PLATON version of 19/02/2022; check.def file version of 19/02/2022

Datablock K12Ge4Sb6 - ellipsoid plot

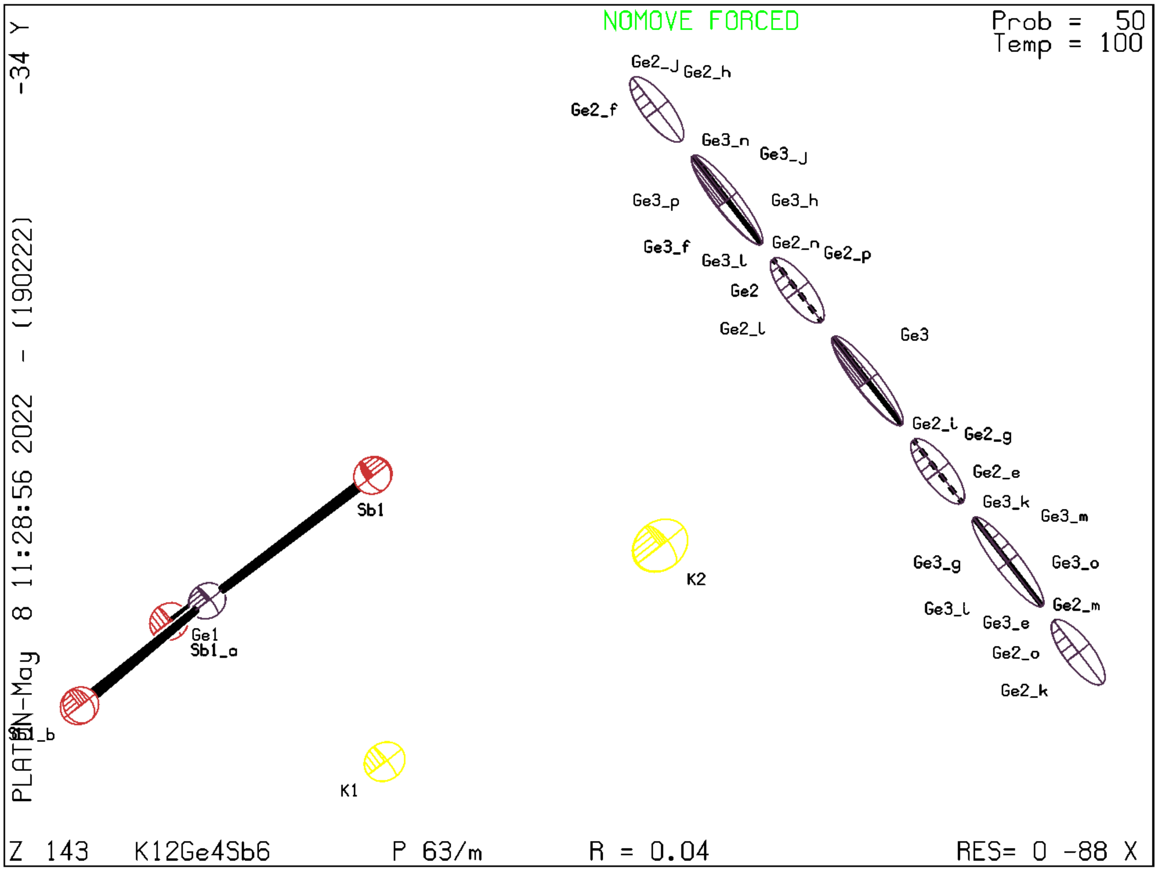

Supplement: Supplementary file 7 — Supporting Information [file ANIE-61-0-s006.pdf]
